# Efficacy of antiseptics and chemomechanical methods for dentin caries lesions: A systematic review with GRADE approach

**DOI:** 10.3389/froh.2023.1110634

**Published:** 2023-02-22

**Authors:** Luiza de Almeida Queiroz Ferreira, Ivana Márcia Alves Diniz, Rogéli Tibúrcio Ribeiro da Cunha Peixoto, Natália Aparecida Gomes, Camila de Sousa Caneschi, Loukia Maria Spineli, Carolina Castro Martins

**Affiliations:** ^1^ Department of Restorative Dentistry, School of Dentistry, Universidade Federal de Minas Gerais, Belo Horizonte, Brazil; ^2^ Midwifery Research and Education Unit, Hannover Medical School, Hannover, Germany; ^3^ Department of Pediatric Dentistry, School of Dentistry, Universidade Federal de Minas Gerais, Belo Horizonte, Brazil

**Keywords:** anti-infecting agents, dental caries, dental disinfectants, systematic review, dentin caries

## Abstract

**Objectives:**

Selective caries removal aims to remove carious tissue in deep dentin lesions. However, a discussion stands on the value of antiseptics and chemomechanical adjuvant methods to reduce the bacterial load on residual caries lesions. This systematic review has addressed two main clinical questions to compare the antimicrobial efficacy of available methods using (1) antiseptic or (2) chemomechanical agents before restoring dentin carious lesions.

**Methods:**

We included randomized and non-randomized controlled trials (RCTs/ NRCTs). We searched eight databases from inception to October 2021. Paired reviewers independently screened studies, extracted data, and assessed the risk of bias. The primary outcome was the reduction in the number of total bacterial in dentin, whereas secondary outcomes were reduction in the number of *Lactobacillus* and *Streptococcus*. We used the ratio of ratio of post-treatment to baseline means between two interventions in the logarithmic scale as a proper effect measure. Certainty of evidence was assessed with the Grading of Recommendations, Assessment, Development and Evaluation approach.

**Results:**

We included 14 RCTs and 9 NRCTs, with nine interventions. Regardless the method, the number of bacteria at baseline was similar or exceeded that after the intervention, particularly in NRCTs. The evidence was inconclusive for most comparisons. Among antiseptic agents, chlorhexidine (CHX) resulted in an average of 1.14 times [95% confidence interval (CI): 1.08–1.21] more total bacterial than photodynamic therapy in RCTs. Among NRCTS, the natural agents resulted in five times more total bacterial than CHX (95% CI: 2–11). For chemomechanical methods, the control resulted in eight times (95% CI: 4–17) more total bacterial than Carisolv (SHAA).

**Conclusions:**

The certainty of the evidence was very low for all comparisons showing uncertainty whether one treatment could be more effective than another for dentin disinfection. So far, exclusively removing soft carious dentin would be enough to reduce the bacterial count.

## 1. Introduction

Aiming to conserve dental hard tissues and avoid pulp exposure, selective carious tissue removal is recommended in dealing with deep carious lesions (1, 2). Meanwhile, there is still professional resistance regarding this technique. Previous studies have shown that clinicians strongly agree that complete caries removal is necessary for dentin caries treatment, thus avoiding the selective removal approach (3, 4). The primary specific reason given by the clinicians to perform complete caries removal is that cariogenic microorganisms must be eliminated or the bacteria left in the dentin may lead to caries lesion progression (4). A set of procedures would then minimize secondary lesions concerns regarding carious tissue removal before restoring the dental cavity, such as dentin antisepsis (2). Although with limited evidence (1),, the use of antisepsis techniques before dental fillings may encourage the selective removal of carious tissue and the maintenance of the dentin still prone to remineralization.

Even though multiple antisepsis techniques and products are commercially available to be used before dental fillings, their effectiveness is questionable, such as ozone and naturally based antiseptic agents (5). Chemomechanical methods, such as Carisolv and papain gel, can also facilitate carious removal and have sodium hypochlorite (NaOCl) or enzyme-based agents in their composition, enabling the antiseptic property. Notwithstanding, doubts regarding its several properties persist, such as the control of the amount of carious tissue removal, time-consuming, and the antiseptic effect (6–8).

Although several systematic reviews have tried to determine the reasonability of using adjunctive antimicrobial therapies as part of the minimally invasive treatment, the studies' substantial methodological and statistical heterogeneity have impaired a more robust conclusion. Those reviews analyzed only single treatments [papain gel or Photodynamic Therapy (PDT)] in both Randomized Clinical Trials (RCTs) and Non-Randomized Clinical Trials (NRCTs) (9–11). One review also included *in vitro* and *in situ* studies (9).

Heretofore, multiple antiseptics' and chemomechanical agents' activity in reducing the bacterial load were never synthesized in only one systematic review of randomized or non-randomized controlled trials (RCTs/NRCTs). Moreover, no previous study has evaluated the effectiveness of antiseptic agents and chemomechanical methods addressed to carious lesions considering the certainty of the evidence. A prior study including only RCTs found very low certainty for the antimicrobial outcome when ozone therapy was evaluated (12). When identified low certainty of evidence from RCTs, relevant NRCTs can be used to complement RCTs' results to allow drawing conclusions through more robust analyses (13). Accordingly, this systematic review of RCTs and NRCTs aimed to answer two clinical questions: what is the efficacy of several (1) antiseptic agents and (2) chemomechanical methods in reducing the number of bacteria in deep carious lesions before dental fillings? Furthermore, we interpreted the results of both clinical questions following the Grading of Recommendations Assessment, Development, and Evaluation (GRADE).

## 2. Material and methods

This systematic review was registered *a priori* at the PROSPERO database (#CRD42020168101) and had one change from the original proposal of a possible subgroup analysis that was not feasible in the end. The original protocol did not plan to divide the interventions into two groups. These changes were performed later, and the protocol was updated at the PROSPERO database as a systematic review aiming to answer two clinical questions (1) antiseptics agents and (2) chemomechanical methods). We report the review according to the Preferred Reporting Items for Systematic Reviews and Meta-Analyses (14).

### 2.1. Eligibility criteria

The PICO questions are:

• Population (P): patients (adults or children) with dentin carious lesion;
• Intervention (I): antiseptic agents [ozone, chlorhexidine (CHX) and photodynamic therapy (PDT)] (question 1); or chemomechanical methods [papain gel and sodium hypochlorite and amino acids (Carisolv—SHAA)] (question 2);
• Comparison (C): negative controls (no use of an antimicrobial treatment or placebo) or comparison with other treatment;
• Outcome (O): reduction of the number of bacteria in dentin before and after treatment.
• Design (D): RCTs and NRCTs, once the first provides the best source of evidence, and the second addresses the effects of interventions when not using randomization to allocate units (individuals or clusters of individuals) to comparison groups. Moreover, NRCTs are ideal for detecting health interventions' potential harms and adverse events (15). We also considered as NRCTs when authors named them as prospective “cohorts” or “case-control studies”, and the intervention groups were allocated during the course of the usual treatment (not randomized), according to the definitions of the ROBINS-I (16). For a proper definition according to ROBINS-I and the GRADE approach, we called these designs as NRCTs (17, 17).

The inclusion criteria comprised RCTs and NRCTs conducted with patients at any age; with deep carious lesions, carious lesions compromising dentin or needing restorative treatment; testing any antiseptic agent or chemomechanical method before dental filling; and measuring the bacterial count before and after the treatment. We excluded studies with a single treatment arm, studies evaluating antimicrobial therapies efficacy in reducing dental biofilm or microorganisms in saliva; studies that evaluated the performance of antimicrobial treatments in preventing dental caries, or as a treatment for enamel carious lesions and periodontal diseases; studies evaluating mouthwashes or substances not directly applied in the dental cavity. We also excluded observational studies with one time-point of evaluation of the outcome.

### 2.2. Information sources

We searched MedLine through Ovid, Embase through Ovid, Cochrane Central Register of Controlled Trials (CENTRAL), Cochrane Database of Systematic Reviews, Web of Science and SCOPUS from inception to October 2021, with no restrictions regarding language and date. We also searched ongoing trials in the WHO International Clinical Trials Registry Platform (ICTRP) and the grey literature in ProQuest Dissertation & Theses Database. The search strategies are presented in Supplementary Table A in S1 File. We organized the references list in Endnote Software (Version X9; Clarivate Analytics).

### 2.3. Study selection

Paired reviewers (LAQF, IMAD, RTRCP, NAG, CSC) independently select studies based on titles and abstracts, and later by reading the full texts using the Rayyan platform (18). Before each screening stage, the reviewers underwent two calibration and training exercises. For screening of titles and abstracts, the reviewers trained with 100 studies. For full-text screening, the reviewers trained with five studies. Disagreements during the calibration and screening were solved by discussion and consensus.

### 2.4. Data extraction and risk of bias assessment

Paired reviewers independently extracted data and assessed the risk of bias of included studies, using a standardized data abstraction form, previously created, and tested. We collected author, year, the language of publication, funding, conflict of interests, country, setting, design of the study, percentage of males and females, initial and final sample, number of drop-outs, number of intervention arms, number of follow-ups, age, type of dentition, type of carious lesion (Black's Classification) (19), complementary exams (x-Ray, Vitality Tests), depth of the lesion, method of carious tissue removal, isolation, anesthesia, number of dentin samples, and bacterial data. We extracted the formulation, concentration, type of carious removal, and dentin limit for every treatment when reported by studies.

For each outcome, we assessed the risk of bias using the Cochrane Risk of Bias Tools for Randomized Trials (RoB 2.0) and The Risk of Bias in Non-Randomized Studies of Interventions (ROBINS I). For ongoing studies, we contacted the three authors regarding the stage of their clinical trials and if they already had data published. Only one author responded that the study was not complete. We also contacted two authors asking for full-text papers when the study was not available, with no response. Nine authors were contacted to provide means and standard deviations (SD) that were not available in the manuscript. Four authors replied by sending the requested data.

### 2.5. Outcomes

The primary outcome was the reduction of total bacterial in deep carious lesions assessed by Real-Time Polymerase Chain Reaction (RTq-PCR) or Colony Forming Unit (CFU) assays. The secondary outcomes were reduction of counts of total *Lactobacillus* and *Streptococcus mutans*. We collected mean, standard deviation (SD), standard error (SE), 95% CI—whether reported—for baseline and after treatment for each microorganism; and pain measurement; and side effects data, when reported.

### 2.6. Data synthesis and statistical analysis

We did not perform a pairwise meta-analysis for the few comparisons with two or three trials due to substantial clinical and methodological heterogeneity in the included trials. Alternatively, for each outcome, we estimated the treatment effect and variance for every comparison in each trial. This analysis corresponds to the fixed-effects model that considers the underlying treatment effects as unrelated and independent for the same comparison (20). We used the ratio of ratio of means (RoRoM) in the logarithmic scale as a proper effect measure. That is the ratio of ratio of post-treatment to baseline means between two compared interventions (21). Herein, a positive log RoRoM (or RoRoM > 1) favors the second intervention in the comparison, a negative log RoRoM (or RoRoM < 1) favors the first intervention in the comparison, and log RoRoM equal zero (or RoRoM = 1) indicates no association between the compared interventions and the investigated outcome. We created a panel of forest plots on the within-trial estimated log RoRoMs for each observed pairwise comparison in the investigated outcomes. We used different line colors, line types and point shapes to depict the trial design (RCT vs. NRCT), method of bacterial counting (CFU vs. q-PCR), and the risk of bias (some concerns vs. high risk), respectively. In the Supplementary S1 File under Statistical methods (*p*. 11), we provide detailed information on the analysis performed. We used descriptive statistics to summarize frequencies of collected study characteristics using the SPSS software version 25 (SPSS, Inc). We used the R-package ggplot2 to obtain all figures (22) and the R-package pcnetmeta (23) to create the network plots.

### 2.7. Certainty of evidence

For each comparison, we assessed the certainty of the evidence through the Grading of Recommendations, Assessment, Development, and Evaluation (GRADE) approach. The certainty of evidence starts with high for RCTs, and NRCTS when using ROBINS-I (17). We assessed the certainty of the evidence for each effect measure generated by each comparison. For effect measures from RCTs, we rated down the certainty if the evidence if there were problems due to risk of bias, inconsistency, indirectness, imprecision, and publication bias. For NRCTs, we started rating down the certainty of the evidence if there were problems due to risk of bias, inconsistency, indirectness, imprecision, and publication bias. Also, we could rate up the evidence for large effect, dose-response, and magnitude of the effects (15, 17).

## 3. Results

### 3.1. Studies included in the systematic review

**Figure 1:**
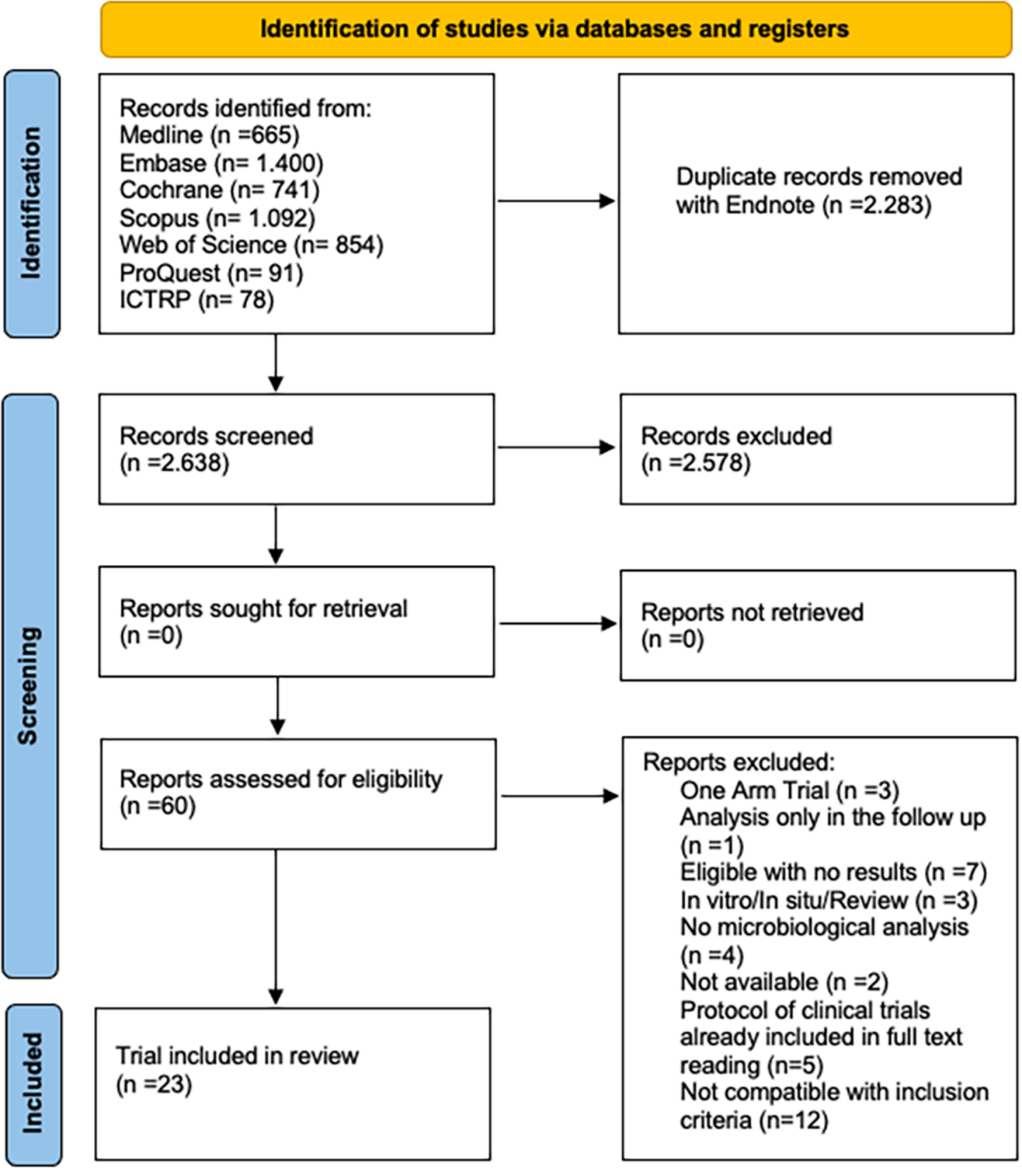
Preferred reporting items for systematic reviews and meta-analyses (PRISMA) flowchart of study screening selection.

Seven RCTs and 5 NRCTs were included for antiseptics agents and 7 RCTs and 4 NRCTs for chemomechanical methods (Figure 1; Supplementary Table B in S1 File shows reasons for exclusion of studies). All studies for both PICO questions were published in English (100%), conducted in Europe (41.7% and 18.2%), Asia (25% and 45.5%), and South America (33.3% and 27.3%), for antiseptics agents and chemomechanical methods, respectively. The majority of trials were published between 2011 and 2020, 75% for antiseptic agents and 81.8% for chemomechanical methods (Table 1).

**Table 1:** Summary of study characteristics.

Characteristics	Antiseptic agents (question 1)	Chemomechanical methods (question 2)
RCTs	7 (58.3%)	7 (63.6%)
NRCTs	5 (41.7%)	4 (36.4%)
**Continents (authors from)**		
Europe^a^	5 (41.7%)	2 (18.2%)
Asia^b^	3 (25.0%)	5 (45.5%)
South America^c^	4 (33.3%)	3 (27.3%)
Africa^d^	0	1 (9.1%)
**Language**		
English	12 (100%)	11 (100%)
**Year of publication**		
2000–2010	2 (16.7%)	1 (9.1%)
2011–2020	9 (75.0%)	9 (81.8%)
2021	1 (8.3%)	1 (9.1%)
**Setting**		
University	12 (100%)	11 (100%)
**Funding**		
Government grant	7 (58.3%)	3 (27.3%)
Not Reported	3 (25.0%)	4 (36.4%)
None	2 (16.7%)	4 (36.4%)
**Conflict of interests**		
No	9 (75.0%)	5 (45.5%)
Not reported	3 (25.0%)	4 (36.4%)
Unclear^e^	0	2 (18.2%)
**Number of intervention arms**		
2	7 (58.3%)	9 (81.8%)
3	3 (25.0%)	2 (18.2%)
4	2 (16.7%)	0
**Dropouts**		
0 Dropouts	6 (50.0%)	8 (72.7%)
1–10 Dropouts	3 (25.0%)	2 (18.2%)
>10	2 (16.7%)	1 (9.1%)
Not reported	1 (8.3%)	0
Median	0.00	0.00
Minimum (*n*)	0	0
Maximum (*n*)	26	31
Total	57	51
**Age**		
Minimum (*n*)^f^	5	4
Maximum (*n*)^f^	48	68
Not reported	2 (18.2%)	0
**% of Women**	182 (51.3%)	155 (49.2%)
Median	18.00	14.50
Minimum (*n*)	4	6
Maximum (*n*)	59	44
Not reported (n)	4 (33.3%)	3 (27.3%)
**Dentition**		
Primary	0	7 (63.6%)
Permanent	5 (41.7%)	3 (27.3%)
Both	6 (50%)	1 (9.1%)
Not reported	1 (8.3%)	0
**Anesthesia**		
Local	7 (58.3%)	2 (18.2%)
Topical	0	2 (18.2%)
None	0	1 (9.1%)
Unclear	0	2 (18.2%)
Not reported	5 (41.7%)	4 (36.4%)
**Isolation**		
Rubber-dam	11 (91.7%)	6 (54.5%)
Cotton	0	4 (36.4%)
Not reported	1 (9.1%)	1 (9.1%)
**x-Ray**		
Yes	10 (83.3%)	9 (81.1%)
Unclear	1 (8.3%)	0
Not reported	1 (8.3)	2 (18.2%)
**Vitality test**		
Eletric	0	1 (9.1%)
Eletric and thermal	4 (33.3%)	1 (9.1%)
Not reported	8 (66.6%)	9 (81.8%)
**Black classification**		
I or II	3 (25.0%)	0
I or IV	0	1 (9.1%)
I	5 (41.7%)	7 (63.6%)
Not reported	4 (33.3%)	3 (27.3%)
**Depth of lesion**		
= or > 2/3 of dentin	6 (50.0%)	3 (27.3%)
<2/3 of dentin	0	2 (18.2%)
Unclear	6 (50.0%)	6 (54.5%)
**Caries removal**		
Soft dentin	5 (41.7%)	6 (54.5%)^g^
Soft and hard dentin	0	2 (18.2%)^g^
Not reported	7 (58.3%)	3 (27.3%)^g^
**Method of removal**		
Bur	7 (58.3)	(2) 18.18
Manual instrument	5 (41.7)	(7) 63.63
Both	0	(2) 18.18^h^
**Number of dentin samples**		
2	7 (58.3%)	11 (100%)
3	3 (25.0%)	0
4	2 (16.7%)	0
**Follow-up (appointments)**		
0	7 (58.3%)	8 (72.7%)
1	1 (8.3%)	3 (27.3%)
2	1 (8.3%)	0
3	3 (25.0%)	0
**Bacterial counting**		
q-PCR	2 (16.7%)	1 (9.1%)
CFU/TVC	10 (83.3%)	10 (90.9%)

RCT, randomized controlled trials; NRCT, non-randomized controlled trials; q-PCR, quantitative *p*olymerase chain reaction; CFU, colony forming units; TVC, total viable count.

^a^ Bosnia and Herzegovina, England, Switzerland, Sweden and Turkey.

^b^ India and Iraq.

^c^ Brazil.

^d^ Egypt.

^e^ Declared no conflict of interests, but dental companies provided the material for research.

^f^ Considering the studies that informed age of patients.

^g^ Considering only control group for PICO question 2 (Chemomechanical methods). For intervention group the method of carious removal was chemomechanical—*n* = 10 (100%).

^h^ Bur in control group and manual instruments for chemomechanical method.

**Figure 2:**
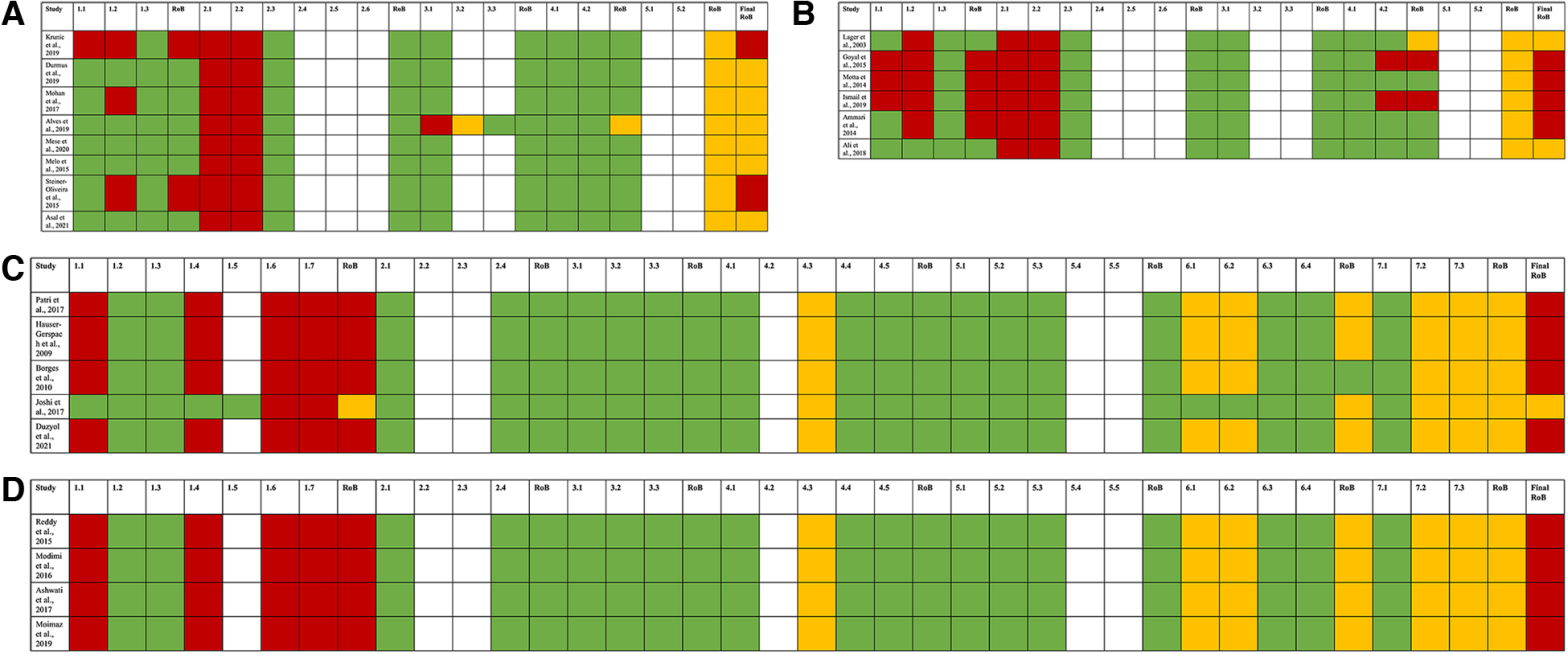
Risk of bias of 7 randomized controlled trials (RCTs) of antiseptic agents assessed through RoB 2.0 (**A**); risk of bias of 7 randomized controlled trials (RCTs) of chemomechanical methods assessed through RoB 2.0 (**B**); risk of bias of 5 non-randomized controlled trials (NRCTs) of antiseptic agents assessed through ROBINS-I (**C**); risk of bias of 4 non-randomized controlled trials (NRCTs) of chemomechanical methods assessed through ROBINS-I (**D**). For RoB 2.0, high risk of bias is represented in red; some concerns are represented in yellow; low risk of bias is represented in green. For ROBINS-I, serious risk of bias is represented in red; moderate is represented in yellow; low risk of bias is represented in green. There was not any study classified as critical risk of bias.

RCTs lacked blinding for the outcome accessor, and 88.8% of NRCTs had a serious risk of bias, presenting potential cofounding (Figure 2).

### 3.2. Primary outcome: total bacterial across the interventions

#### 3.2.1. Distribution of the outcome across NRCTs and RCTs

The average number of total bacterial (before and after intervention) was considerably variable, particularly across the NRCTs (range: 3.10–7.82 × 10^7^ in NRCTs, and 1.20 × 10^3^–5.86 × 10^8^ in RCTs). Even for the same intervention, the average number of total bacterial was substantially variable across the corresponding trials (Supplementary S1 Figure in S1 File). Overall, the average number of total bacterial at baseline for both antiseptic agents and chemomechanical methods was similar or exceeded that after receiving the intervention. The difference in the average number of total bacterial before and after the intervention was profound in the NRCTs. Similar observations were made concerning the SD of total bacterial (range: 1.20–4.10 × 10^6^ in NRCTs, and 7.00 × 10^2^–6.70 × 10^7^ in RCTs) (Supplementary S2 Figure in S1 File). The coefficient of variation was consistently below one in all NRCTs, indicating a lower variability of the total bacterial relative to the mean. However, it exceeded one in three RCTs investigating the control, ozone and SHAA at baseline, and one RCT investigating CHX before and after the intervention (Supplementary S3 Figure in S1 File).

#### 3.2.2. Panel of forest plots: antiseptic agents

Of the 9 observed comparisons investigated in RCTs, only two provided conclusive evidence about the average reduction in the total bacterial: CHX vs. PDT (1.14, 95% CI: 1.08–1.21) and natural agents vs. control (0.14, 95% CI: 0.02–0.93). Hence, compared to natural agents, the control resulted on average seven (i.e., 1/0.14) times more total bacterial, while CHX yielded slightly more total bacterial than PDT (Table 2).

**Table 2:** Clinical interpretation for each comparison considering three outcomes (total bacterial, *Lactobacillus,* and *Streptococcus mutans*).

Question	Study	Caries removal	Clinical interpretation		
			Total bacterial	*Lactobacillus*	*Streptococcus mutans*
1	Patri and Sahu, 2017^3^ (24)	Not informed	Control presents an average of 20 × more bacteria than CHX *	* *	* *
			Control presents an average of 4.5 × more bacteria than Natural Agents*	* *	* *
			Natural Agents presents an average of 4.77 × more bacteria than CHX*	* *	* *
1	Uday Mohan et al., 2016^4^ (25)	Selective	Control presents an average of 4 × more bacteria than CHX*	Control presents an average of 14e-02 × more *Lactobacillus* than CHX*	Control presents an average of 12.5 × more *Streptococcus mutans* than CHX*
			CHX presents an average of 5e + 00 × more bacteria than Laser*	CHX presents an average of 2.9e-03 × more *Lactobacillus* than Laser*	CHX presents an average of 4 × more *Streptococcus mutans* than Laser*
			CHX presents an average of 1.6 × more bacteria than Natural Agents*	CHX presents an average of 1.7e-02 × more *Lactobacillus* than Natural Agents*	CHX presents an average of 2.8 × more *Streptococcus mutans* than Natural Agents*
			Control presents an average of 7.1 × more bacteria than Natural Agents*	Control presents an average of 25e-02 × more *Lactobacillus* than Natural Agents*	Control presents an average of 33.3 × more *Streptococcus mutans* than Natural Agents*
			Laser presents an average of 3.09e + 01 × more bacteria than Natural Agents*	Laser presents an average of 1.68e + 05 × more *Lactobacillus* than Natural Agents*	Laser presents an average of 1.39 × more *Streptococcus mutans* than Natural Agents*
1	Joshi et al., 2017^3^ (26)	Selective	CHX presents an average of 1 × more bacteria than control*	Control presents an average of 1 × more *Lactobacillus* than CHX*	Control presents an average of 1.12 × more *Streptococcus mutans* than CHX*
1	Krunic et al., 2019^4^ (27)	Not informed	Ozone presents an average of 2.05 × more bacteria than CHX*	Ozone presents an average of 2.04 × more *Lactobacillus* than CHX*	
1	Hauser-gerspach et al., 2009^3^ (28)	Not Informed	CHX presents an average of 1× more bacteria than Ozone*		
1	Steiner-Oliveira et al., 2015^4^ (29)	Not informed	PDT presents an average of 1.14 × more bacteria than CHX*		PDT presents an average of 1.19 × more *Streptococcus mutans* than CHX*
1	Melo et al. 2015^4^ (30)	Not informed	Control presents an average of 1.1 × more bacteria than PDT*	Control presents an average of 1.20 × more *Lactobacillus* than PDT*	Control presents an average of 1.51 × more *Streptococcus mutans* than PDT*
1	Düzyol et al., 2021^3^ (31)	Selective		Ozone presents an average of 1.05 × more *Streptococcus mutans* the CHX*	Ozone presents an average of 1 × more *Streptococcus mutans* the CHX*
2	Moimaz et al., 2019^3^ (32)	Selective		Control presents an average of 1,61e-01 × more *Lactobacillus* than papain gel*	Control presents an average of 1.39 × more *Streptococcus mutans* than papain gel*
				Papain Gel presents an average of 1.08 more *Lactobacillus* than SHAA*	SHAA presents 2.26 more *Streptococcus mutans* than papain gel*
				Control presents an average of 1.72 × more *Lactobacillus* than SHAA*	Control presents an average of 1.61 × more *Streptococcus mutans* than SHAA*
2	Goyal et al., 2015^4^ (33)	Not informed	Control presents an average of 1.01 × more bacteria than Papain Gel*	Papain Gel presents an average of 1.36 × more *Lactobacillus* than control*	
2	Savitha et al. 2017^3^ (34)	Not informed	Papain Gel presents an average of 1.01 × more bacteria than control*		
2	Modimi et al. 2016^3^ (35)	Not informed		Control presents an average of 1.1 × more *Lactobacillus* than Papain Gel*	Control presents an average of 1.6 × more *Streptococcus mutans* than Papain Gel*
2	Ali et al. 2018^4^ (36)	Selective	SHHA presents 7.6 × more bacteria than control*		
2	Reddy et al. 2015^3^ (37)	Selective	Papain Gel presents an average of 1.03 × more bacteria than SHAA group*	Papain Gel presents an average of 1.03 × more *Lactobacillus* than SHAA*	
2	Asal et al. 2021^4^ (38)	Complete	Control presents an average of 1.40 × more bacteria than SHAA group*		

Full Summary of Findings (SoF) for certainty of evidence is presented at Supplementary Material; (1) Antiseptic agents; (2) Chemomechanical methods; (*) All comparisons had very low certainty of evidence; (3) NRCT; (4) RCT. Light gray cells indicate conclusive results according to the analysis for NRCTs. Dark gray cells indicate conclusive results for RCTs. White cells indicate inconclusive results with the 95%CI crossing the line of null effect.

Three in five comparisons investigated in NRCTs yielded conclusive results, with CHX vs. control having the most significant treatment effect (0.05, 95% CI: 0.02–0.11), followed by CHX vs. natural agents (0.21, 95% CI: 0.09–0.51)—both comparisons favored CHX. The control and natural agents resulted on average in 20 (i.e., 1/0.05) and five (i.e., 1/0.21) times more total bacterial than CHX (Supplementary Table C in S1 File). Reducing the correlation to 0.6 increased the SE of log RoRoM, as expected, and led to inconclusive results only for CHX vs. ozone (28) (Supplementary S4 and S5 Figure in S1 File).

**Figure 3:**
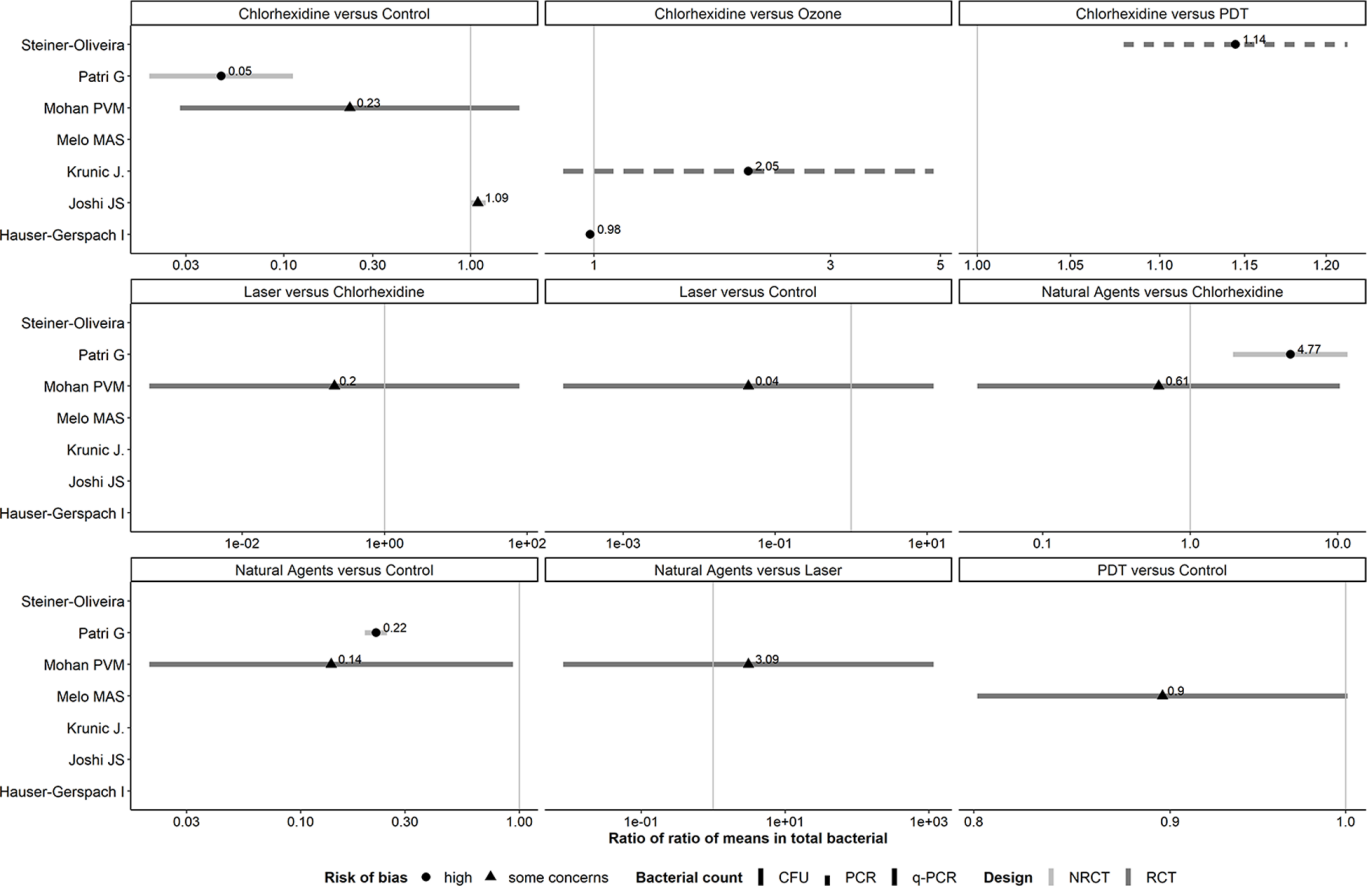
Forest plots showing roRoMs for total bacterial across the antiseptics agents.

Comparisons investigated in both trial designs were associated with inconsistent evidence in magnitude and conclusiveness (Figure 3).

#### 3.2.3. Panel of forest plots: chemomechanical methods

**Figure 4:**
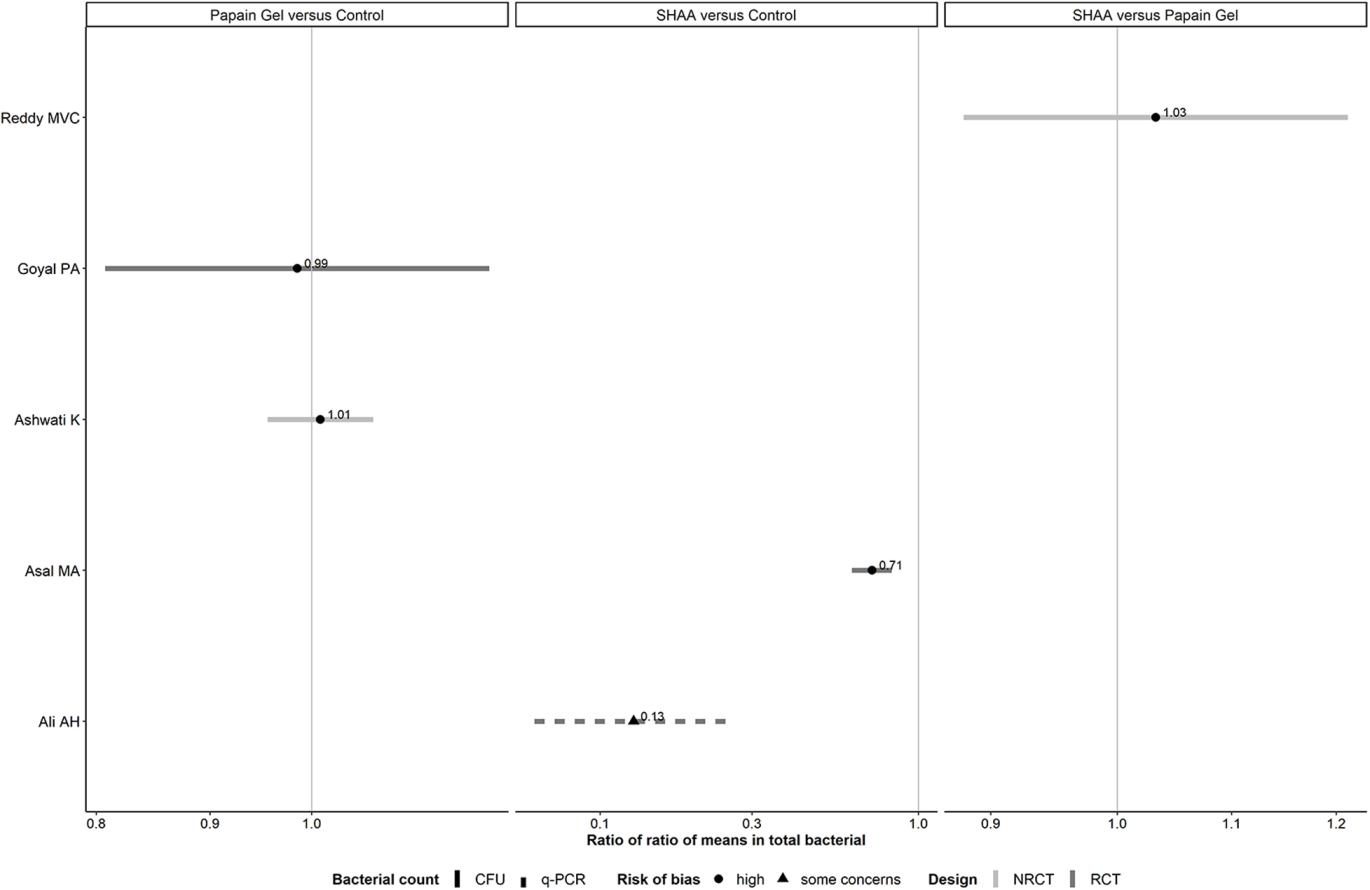
Forest plots showing roRoMs for total bacterial across chemomechanical methods.

Two RCTs investigating chemomechanical methods (39, 40) did not report the SD in either arm and were excluded from the analysis. Comparisons investigated in both trial designs were associated with inconsistent evidence in magnitude and conclusiveness (Figure 4). Overall, RoRoMs were estimated with greater precision in NRCTs than RCTs, as indicated by the range of values on the x-axis.

Of the two observed comparisons investigated in RCTs, only one provided conclusive evidence about the average reduction in the total bacteria: SHAA vs. control (0.13, 95% CI: 0.06–0.26). Hence, compared to SHAA, the control resulted on average, in eight (i.e., 1/0.13) times more total bacterial (Table 2).

### 3.3. Secondary outcomes: Lactobacillus and Streptococcus mutans

#### 3.3.1. Distribution of the outcome across NRCTs and RCTs

The average number of total *Lactobacillus* (before and after intervention) was considerably variable within and across interventions but to a lesser extent than in the primary outcome (Supplementary S6 Figure in S1 File). Like the primary outcome, NRCTs exerted greater variability in the number of *Lactobacillus* (range: 29.6–1.92 × 10^5^ in NRCTs, and 2.0 × 10^2^–6.94 × 10^4^ in RCTs). Overall, the average number of total *Lactobacillus* at baseline was similar or exceeded that after receiving the intervention, and the difference between baseline and post-intervention average number of total *Lactobacillus* was more profound in the NRCTs. Similar observations were made concerning the SD of total *Lactobacillus* (range: 52.0–4.72 × 10^4^ in NRCTs, and 3.00 × 10^2^–1.50 × 10^5^ in RCTs) (Supplementary S7 Figure in S1 File). Opposing the primary outcome, the coefficient of variation was below one in 67% of the trial-arms in NRCTs both at baseline and post-intervention, indicating a lower variability of the total *Lactobacillus* relative to the mean. A similar percentage of trial-arms in RCTs yielded a coefficient of variation below one. Specifically, this was the case for two trials investigating the control and the unique trial comparing natural agents (at baseline). Also, for PDT, papain gel and the trial investigating the control at both time-points (Supplementary S8 Figure in S1 File).

Regardless the method, overall, data on the distribution of the average and SD of *S. mutans* were in line with that of the *Lactobacillus* (range of average: 1.04 × 10^2^–1.67 × 10^5^ in NRCTs, and 2.0 × 10^2^–2.37 × 10^8^ in RCTs; range of standard deviation: 8.76 × 10–4.80 × 10^4^ in NRCTs, and 5.00 × 10^2^–5.00 × 10^7^ in RCTs) (Supplementary S9–11 Figures in S1 File).

#### 3.3.2. Panel of forest plots for *Lactobacillus*: antiseptic agents

**Figure 5:**
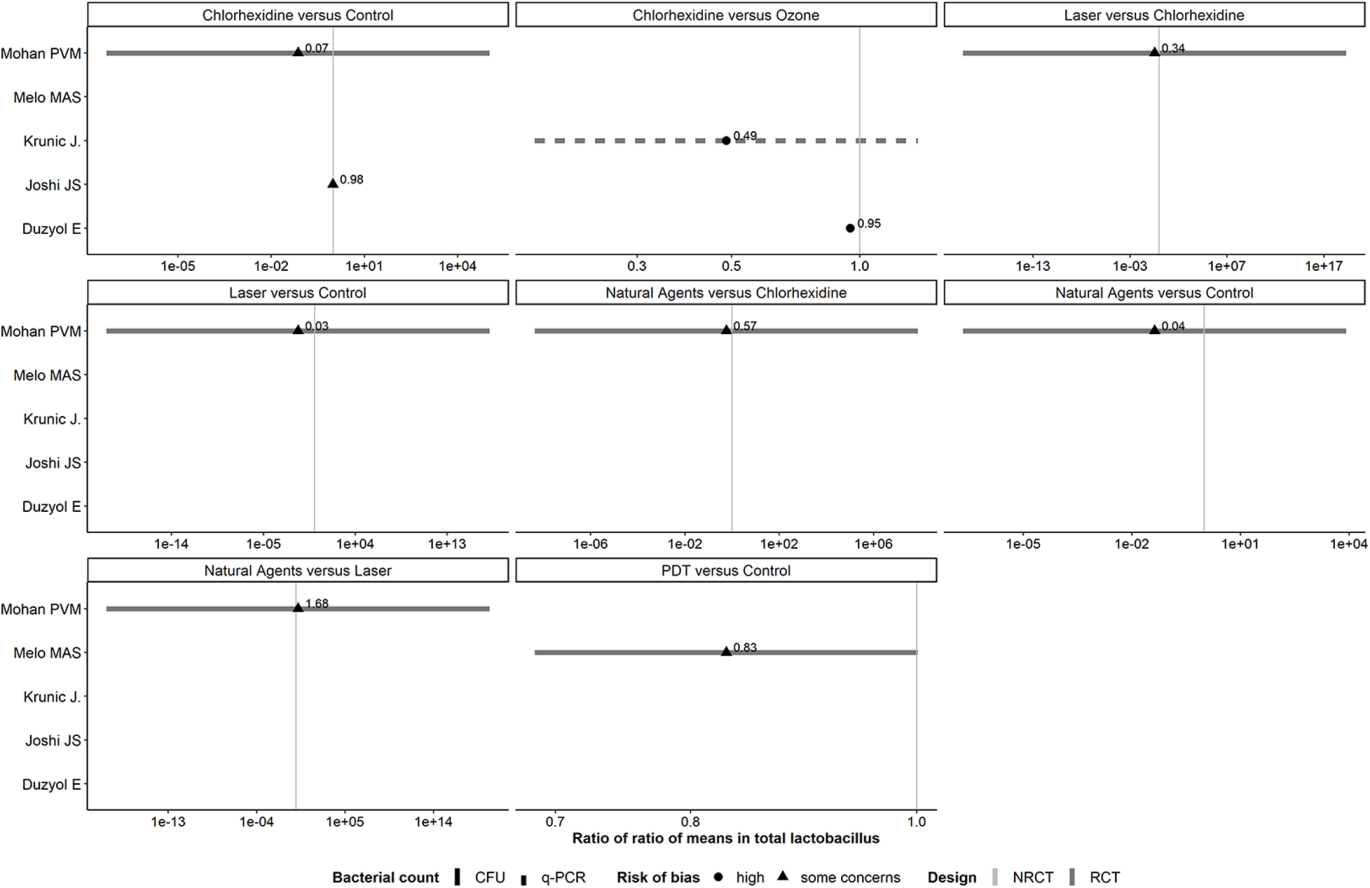
Forest plots showing roRoMs for *Lactobacillus* across the antiseptics agents.

One comparison investigated in both trial designs (CHX vs. control) was associated with inconsistent evidence in magnitude and conclusiveness (Figure 5). One RCT (25) was responsible for this wide range of SE (range: 6.24–23.32) that lowered our confidence in the credibility of the corresponding 95% CIs. This trial had substantial SD in either arm at post-treatment, which exceeded the overall average count.

Regardless the method, overall, RoRoMs were estimated with more precision in NRCTs than RCTs (range of standard error of log RoRoM: 0.04–4.19 in NRCTs, and 0.10–23.32 in RCTs), as indicated by the range of values on the x-axis. None of the observed comparisons provided conclusive evidence, as all 95% CIs crossed the vertical line of no difference (Supplementary Table D in S1 File). Reducing the correlation to 0.6 increased slightly the SE of log RoRoM, as expected (range of SE of log RoRoM: 0.05–4.31 in NRCTs, and 0.12–23.39 in RCTs) (Supplementary S12 and S13 Figures in S1 File).

#### 3.3.3. Panel of forest plots for *Lactobacillus*: chemomechanical methods

**Figure 6:**
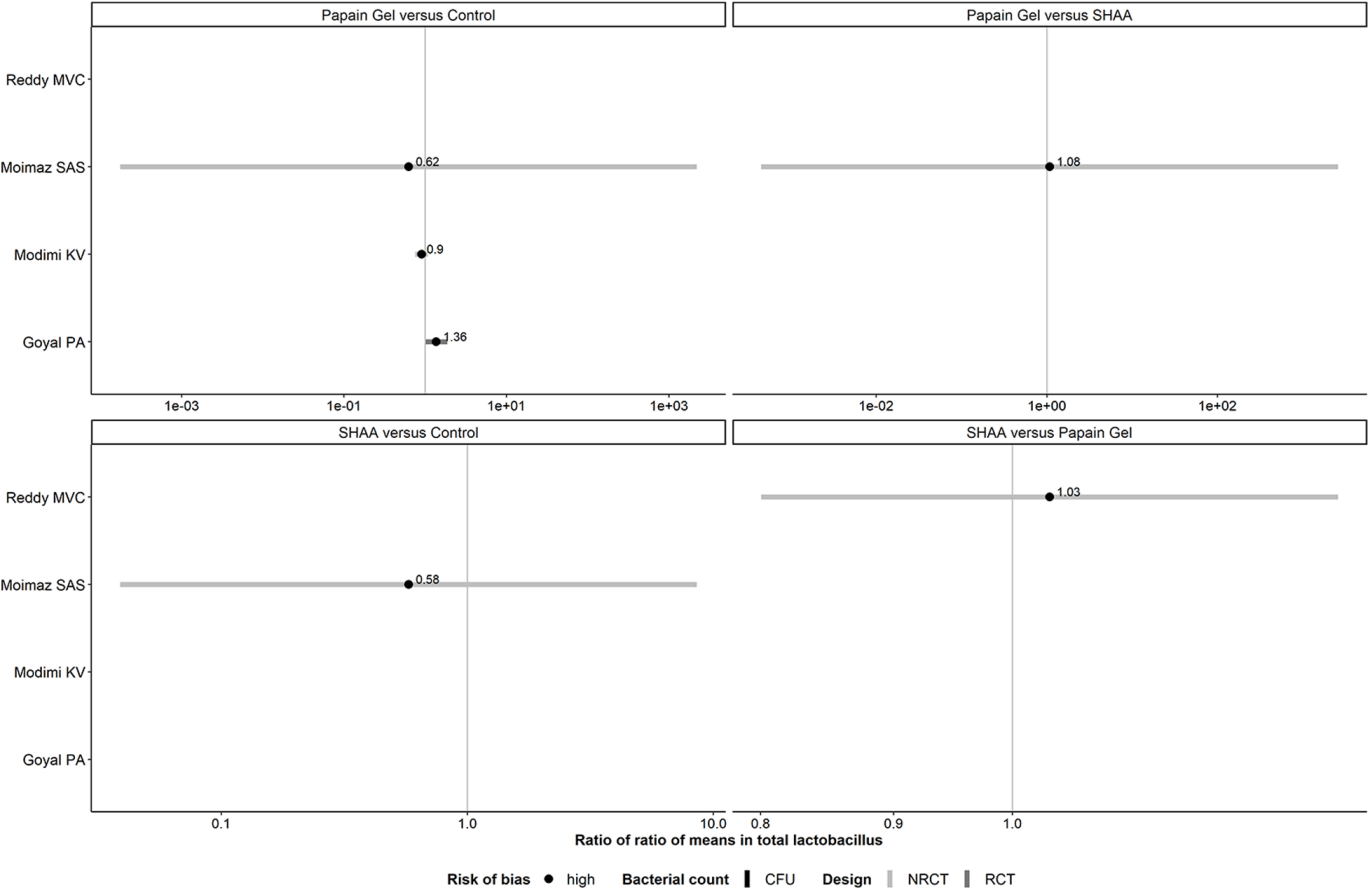
Forest plots showing roRoMs for *Lactobacillus* across chemomechanical methods.

For *Lactobacillus*, one RCT (39) did not report the SD in either arm and was excluded from the analysis. One comparison investigated in both trial designs (papain gel vs. control) was associated with inconsistent evidence in magnitude and conclusiveness (Figure 6).

#### 3.3.4. Panel of forest plots for *Streptococcus mutans*: antiseptic agents

**Figure 7:**
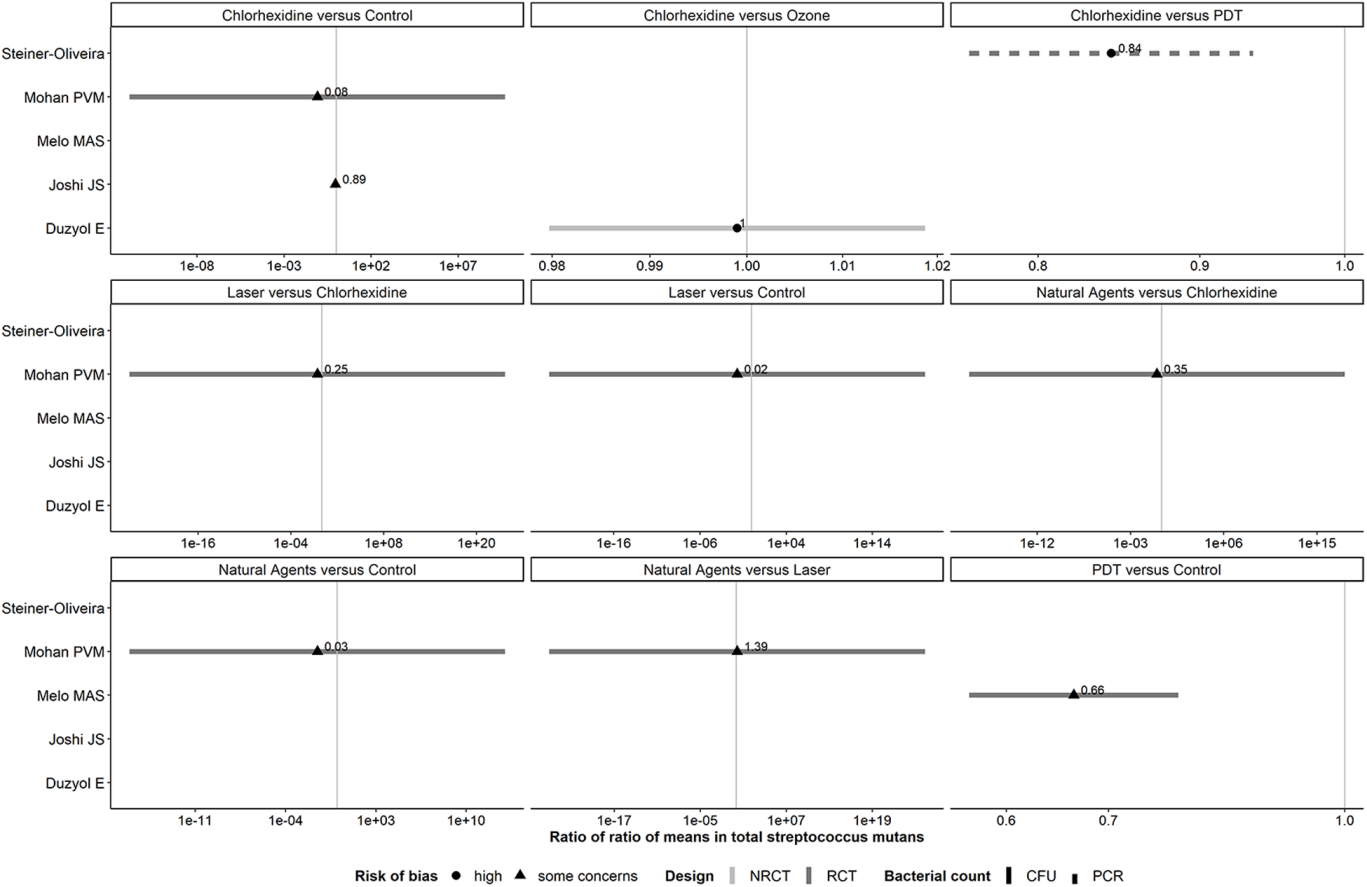
Forest plots showing roRoMs for *Streptococcus mutans* across the antiseptics agents.

For *S. mutans*, one RCT by Mohan PVM (25) yielded the particularly large SE of log RoRoM for all comparisons (range: 12.74–30.92) that lower our confidence in the credibility of the corresponding 95% CIs. This trial had substantial SD in either arm at post-treatment that exceeded the overall average count at baseline that greatly exceed the baseline SD in all arms. (Figure 7).

PDT vs. control, as well as CHX vs. PDT and control yielded conclusive results in favor of the former intervention in each comparison (0.66, 95% CI: 0.57–0.78; 0.84, 95% CI: 0.76–0.93 and 0.89, 95% CI: 0.81–0.98 respectively) (Supplementary Table E in S1 File). Reducing the correlation to 0.6 increased the SE of log RoRoM, as expected (Supplementary S14 and S15 Figures in S1 File).

#### 3.3.5. Panel of forest plots for *Streptococcus mutans*: chemomechanical methods

**Figure 8:**
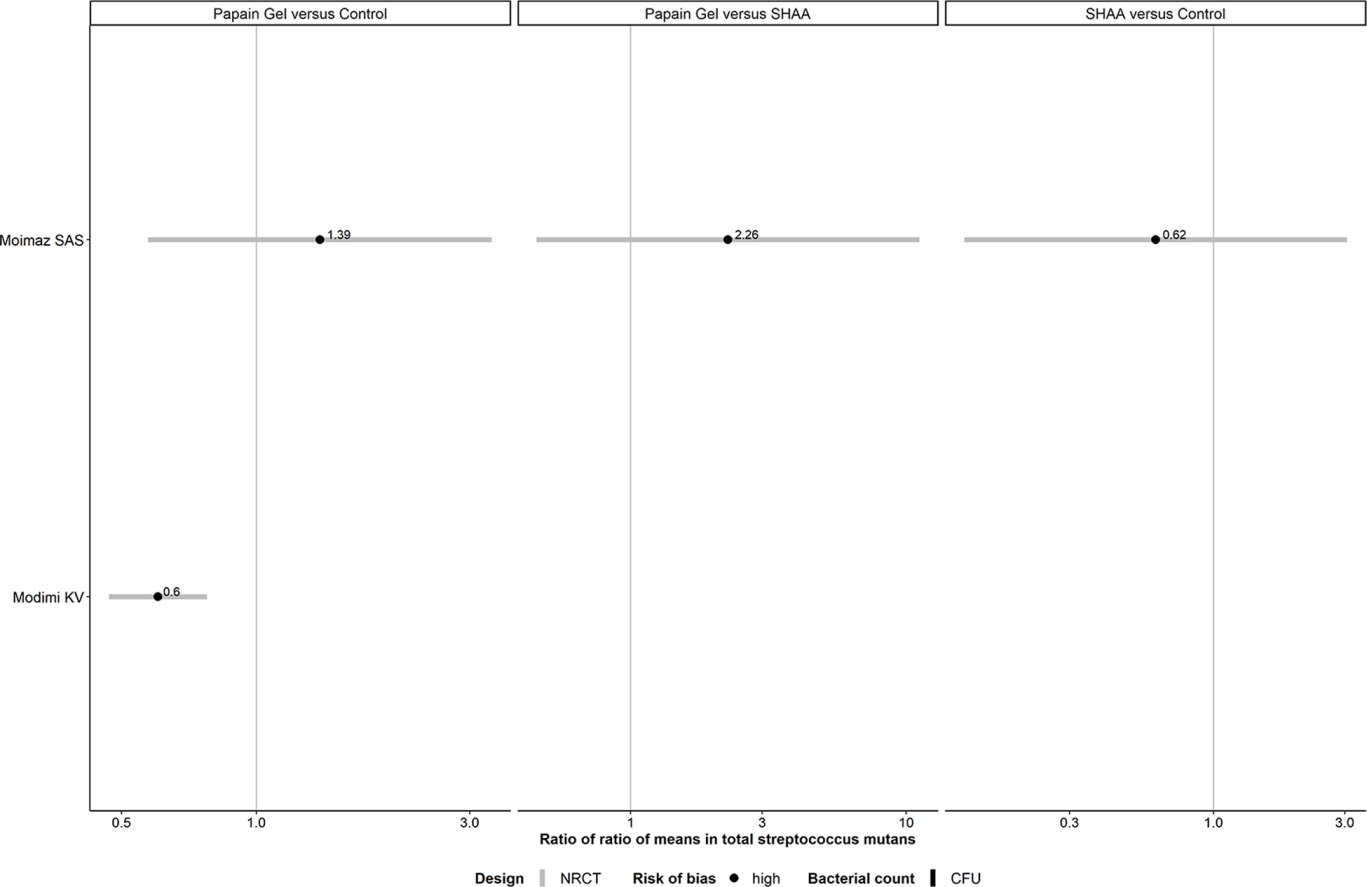
Forest plots showing roRoMs for *Streptococcus mutans* across chemomechanical methods.

Overall, RoRoMs were estimated with more precision in RCTs than NRCTs (range of SE of log RoRoM: 0.06–1.00 in NRCTs, and 0.07–0.11 in RCTs), as indicated by the range of values on the x-axis (Figure 8).

Papain gel vs. control yielded conclusive results in favor of the former intervention (0.60, 95% CI: 0.47–0.78) (Supplementary Table E in S1 File). Reducing the correlation to 0.6 increased the SE of log RoRoM, as expected (Supplementary S14 and S15 Figures in S1 File).

### 3.4. Pain

Only studies for chemomechanical methods analyzed the outcome pain. One study (33) measured pain and mean pulse rate/minute during the treatments. The control resulted in less pain compared to papain gel during and after procedures (Supplementary S16, 1S7 Figures in S1 File, Supplementary Table F in S1 File). No study reported side effects of treatments.

### 3.5. Grade approach and clinical interpretation

Summary of findings (SoF) table shows the reasons for a rating down the certainty of the evidence, that was very low for all antiseptic agents (Supplementary Tables G–I in S1 File), chemomechanical methods (Supplementary Tables J–L in S1 File), and pain outcomes (Supplementary Table M in S1 File. The certainty was rated down due to risk of bias, indirectness, imprecision, and publication bias. Table 2 shows the clinical interpretation for total bacterial, *Lactobacillus*, and *Streptococcus mutans* regarding antiseptic agents and chemomechanical methods.

## 4. Discussion

The present systematic review of multiple agents did not include an NMA. Therefore, we cannot offer a hierarchy of interventions from the best to the worst for each outcome. Furthermore, the evidence supporting direct comparisons was very low. Most of the time, the antiseptic agents/chemomechanical methods or even the non-disinfectant control reduced the count of bacterial in accordance with other studies (5, 9–11).

The reference group consisted only in removing carious tissue, i.e., neither antiseptic nor a chemomechanical method was applied. Our analysis demonstrated that this treatment alone would be enough to reduce the bacterial load. Even relying on subjective parameters, the selective removal of carious tissue is recommended for managing deep lesions. This procedure is based on removing peripheral soft carious dentin, which presents heavy bacterial contamination (1, 41, 42). By removing exclusively the dental tissue not capable of remineralization (soft dentin) the chances of pulp recovery is increased and the preserved firm dentin guarantees the survival of fillings (42).

Concerning NRCTs, our data favored CHX as the most effective treatment compared to the non-antiseptic control and natural agents. Nevertheless, we obtained inconclusive results comparing CHX vs. ozone. In this regard, previous reports also showed some discrepancies when comparing CHX and ozone treatment, where conflicting results were narratively synthesized from the analyzed literature. Altogether, the results of two studies in a meta-analysis showed that CHX was significantly better than ozone in reducing bacterial load in the dentin tissue, although no comparison was made concerning a non-disinfected group (12).

Regarding the analysis of RCTs, our data showed that PDT presented a slight reduction of total bacterial in carious lesions compared to CHX. Results of this comparison is similar to the narrative synthesis presented by Cieplik et al. 2017 (9), where the reduction of PDT occurred for total bacterial, *Streptococcus* and *Lactobacillus* compared with 2% CHX. In previous reports, PDT presented controversial results about the total viable bacteria compared to the conventional drilling control group (9, 11). Accordingly, a meta-analysis of four studies—where two were *in vitro*—showed the efficacy of PDT in reducing microorganisms' numbers (11). Otherwise, another descriptive systematic review found five studies where PDT associated with mechanical caries removal reduced cariogenic bacteria in dentin lesions, whereas one study pointed to PDT as an ineffective agent (9).

Concerning chemomechanical methods, bacterial load was greater in the SHAA/natural agents when compared to a reference control without the use of an adjunctive therapy. Different results were found comparing the SHAA and papain gel with the control group with a rotatory instrument. No differences were found between any treatment regarding the reference control (8). In our review, concerning *Lactobacillus,* two studies found that the control presented more bacteria than the papain gel or SHAA, according to other studies (32, 35). However, all comparisons for *Lactobacillus* were inconclusive when comparing both active treatments, SHAA, and papain gel. The efficacy of these treatments was similar in reducing the dentin bacterial load. For *S. mutans*, our results were conclusive and pointed to a reduction of *S. mutans* in the papain gel group vs. the reference control. These results align with previous studies (32, 37). Systematic data from the comparison of the Papacarie—a brand of Papain Gel—and a non-disinfected control using conventional drilling method concluded that the gel successfully reduced the bacterial load in deciduous teeth. However, the meta-analysis was restricted to two studies (10).

There were serious and very serious problems due to risk of bias. Several RCTs did not present information about the randomization process or blinding of the outcome assessor; the last is critical for the CFU analysis, for example. Eighty-seven percent of NRCTs had serious risk of bias and potential confounding factors. We considered a potential confounding factor when the dentin was not removed in a standardized way (i.e., lack of weighting the removed dentin) in the risk of bias. This factor resulted in a serious risk of bias and consequently impaired the certainty of evidence. There were very serious problems due to indirectness in all comparisons. Most of the trials included only permanent or deciduous teeth. There was great heterogeneity concerning the methods of carious tissue removal, being either rotatory or manual instruments. Some studies used relative isolation or rubber dam or analyzed only teeth with occlusal cavities. Hence, the applicability is limited to the general dental practice. The inclusion of a single trial in each comparison did not achieve the *Optimal Information Size* (OIS) of at least 400 participants, inputting imprecision (43). Finally, 8.6% of comparisons were rated down due to possible publication bias considering industry-sponsored studies.

We could not perform subgroup or sensitivity analysis, initially aimed in the protocol, as we had one trial per comparison. Notwithstanding, the collected data stemmed from immediate cleansing of the cavities and not a long-term analysis on how these treatments would affect the adjacent dentin or the survival of dental fillings. Only one study included in the review evaluated this outcome in a follow-up of six months, and no differences were found between the antiseptic group (PDT) and the non-antiseptic group. The following parameters were analyzed: retention, marginal adaptation, marginal discoloration, secondary caries, and color (44). Thus, the evidence brought up by this review is limited for immediate restorations and may not be applicable for long-term pulp health and restoration success. This review is strong as we interpret it based on the certainty of evidence and the average bacterial count for the clinical practice. It is noteworthy that the methods for assessing bacterial load before and after treatments (RT-qPCR or CFU) may differ in their ability to determine live and dead microorganisms. Although RT-qPCR contributes to the identification of a diversity of bacterial strains, it does not determine the presence of viable cells (45). Therefore, RT-qPCR should be cautiously interpreted since this method could underestimate the antimicrobial activity results. Conversely, CFU may allow the growth analyses of some microorganisms; in the meantime, the variety of strains is limited to the broth and cell culture conditions.

Non-selective caries removal can expose less contaminated dentin to antiseptic agents. Accordingly, data from Table 2 should be interpreted considering the method of caries tissue removal. Most of the works included in the review used selective removal, eliminating only the soft dentin (41.7% for antiseptics and 54.5% for chemomechanical). Recently, a systematic review comparing multiple factors regarding selective, stepwise, and non-selective removal concluded in a narrative synthesis that selective and non-selective removal effectively reduced the microbial load in dentin lesions, without statistical difference between the techniques (46).

Due to very low certainty, we cannot affirm that there is one better treatment than the other (47, 48). The antiseptic treatments and chemomechanical methods majorly reduced the bacterial load in dentin carious lesions but with similar results for the non-disinfected control. In the opportunity of testing new technologies as adjunctive treatments for stopping carious lesions progression, a complete description of the methodology is required to indicate how the antiseptic treatments should be performed operatively and how they could affect the dentin or pulp tissues. Follow-up trials could bring new data on the long-term survival of restorations and report the occurrence of secondary caries. So far, the antiseptics and chemomechanical methods have a trivial or similar effect when compared to the removal of infected carious dentin to reduce bacterial count.

## Data Availability

The original contributions presented in the study are included in the article/**Supplementary Material**, further inquiries can be directed to the corresponding author/s.
