# Supplementary material for: Efficacy of antiseptics and chemomechanical methods for dentin caries lesions: A systematic review with GRADE approach
Source: Front Oral Health. 2023 Feb 22;4:1110634. doi: 10.3389/froh.2023.1110634 (PMC9992646; doi:10.3389/froh.2023.1110634)
Supplement: Supplementary file 1 [file Datasheet1.docx]

Supplementary Material

Efficacy of antiseptics and chemomechanical methods for dentin caries lesions: a systematic review with GRADE approach

**Luiza de Almeida Queiroz Ferreira^1^, Ivana Márcia Alves Diniz^1*^, Rogéli Tibúrcio Ribeiro da Cunha Peixoto^1^, Natália Aparecida Gomes^1^, Camila de Sousa Caneschi^1^, Loukia Maria Spineli^2^, Carolina Castro Martins^3*^**

^1^Department of Restorative Dentistry, School of Dentistry, Universidade Federal de Minas Gerais, Belo Horizonte, Minas Gerais, Brazil.

^2^Midwifery Research and Education Unit, Hannover Medical School, Hannover, Germany.

^3^Department of Pediatric Dentistry, School of Dentistry, Universidade Federal de Minas Gerais, Belo Horizonte, Minas Gerais, Brazil.

*** Correspondence:**

Ivana Márcia Alves Diniz

[ivanadiniz@ymail.com](mailto:ivanadiniz@ymail.com)

Carolina Castro Martins

[carolcm10@hotmail.com](mailto:carolcm10@hotmail.com)

**Table A.** Search strategies used according to electronic databases (date: from inception to January 2019, updated on October 2021).

| **Medline through Ovid**  1 chlorhexidine.mp.  2 chlorhexidine bigluconate.mp.  3 chlorhexidine digluconate.mp.  4 chlorhexamed.mp.  5 hexidine.mp.  6 papain.mp.  7 papacarie.mp.  8 antimicrobial photodynamic therapy.mp.  9 photochemotherap*.mp.  10 photodynamic therap*.mp.  11 ozone.mp.  12 ozone therap*.mp.  13 carisolv.mp.  14 leucine.mp.  15 lysine.mp.  16 "glutamic acid".mp.  17 photostimulation.mp.  18 photoirradiation.mp.  19 photoactivation.mp.  20 photobiomodulation.mp.  21 PBMT.mp.  22 PBM.mp.  23 LLLT.mp.  24 diode laser.mp.  25 laser.mp.  26 light emitting diode.mp.  27 red laser therap*.mp.  28 low level laser therap*.mp.  29 phototherapy.mp.  30 low-level light therap*.mp.  31 low power laser irradiation.mp.  32 low-power laser irradiation.mp.  33 photobiomodulation therap*.mp.  34 laser biostimulation.mp.  35 laser phototherap*.mp.  36 1 or 2 or 3 or 4 or 5 or 6 or 7 or 8 or 9 or 10 or 11 or 12 or 17 or 18 or 19 or 20 or 21 or 22 or 23 or 24 or 25 or 26 or 27 or 28 or 29 or 30 or 31 or 32 or 33 or 34 or 35 (281403)  37 dental caries.mp.  38 caries.mp.  39 dental decay.mp.  40 caries, dental.mp.  41 decay, dental.mp.  42 carious dentin*.mp.  43 cavitated teeth.mp.  44 cavitated tooth.mp.  45 37 or 38 or 39 or 40 or 41 or 42 or 43 or 44 (54404)  46 randomized controlled trial.mp.  47 clinical trial.mp.  48 non-randomized trial.mp.  49 random*.mp.  50 placebo.mp.  51 46 or 47 or 48 or 49 or 50  52 36 and 45 and 51 |
| --- |
| **Embase through Ovid**   \| 1. chlorhexidine.mp. or exp chlorhexidine/ or exp 2 propanol plus chlorhexidine gluconate/ or exp chlorhexidine gluconate/ \|  \| \| --- \| --- \| \| 2. chlorhexidine bigluconate.mp. \|  \| \| 3. chlorhexidine digluconate.mp. \|  \| \| 4. chlorhexamed.mp. \|  \| \| 5. xexidine.mp. \|  \| \| 6. papain.mp. or exp papain/ \|  \| \| 7. papacarie.mp. \|  \| \| 8. exp photodynamic therapy/ or antimicrobial photodynamic therapy.mp. or exp photochemotherapy/ \|  \| \| 9. photochemotherap*.mp. \|  \| \| 10. photodynamic Therap*.mp. \|  \| \| 11. ozone.mp. or exp ozone/ or exp ozone therapy/ \|  \| \| 12. ozone therap*.mp. \|  \| \| 13. photostimulation.mp. or exp photostimulation/ \|  \| \| 14. photoirradiation.mp. \|  \| \| 15. photoactivation.mp. or exp photoactivation/ \|  \| \| 16. photobiomodulation.mp. \|  \| \| 17. PBMT.mp. \|  \| \| 18. PBM.mp. \|  \| \| 19. LLLT.mp. \|  \| \| 20. exp diode laser/ or laser.mp. or exp laser/ \|  \| \| 21. light emitting diode.mp. or exp light emitting diode/ \|  \| \| 22. red laser therap*.mp. \|  \| \| 23. exp low level laser therapy/ or low level laser therap*.mp. \|  \| \| 24. exp phototherapy/ or low-level light therap*.mp. \|  \| \| 25. exp laser/ or low power laser irradiation.mp. or exp low level laser therapy/ \|  \| \| 26. low-power laser irradiation.mp. \|  \| \| 27. photobiomodulation therap*.mp. \|  \| \| 28. laser biostimulation.mp. \|  \| \| 29. laser phototherap*.mp. \|  \| \| 30. dental Caries.mp. or exp dental caries/ \|  \| \| 31. caries.mp. \|  \| \| 32. dental decay.mp. \|  \| \| 33. caries, dental.mp. \|  \| \| 34. decay, dental.mp. \|  \| \| 35. carious dentin*.mp. \|  \| \| 36. cavitated teeth.mp. \|  \| \| 37. cavitated tooth.mp. \|  \| \| 38. 30 or 31 or 32 or 33 or 34 or 35 or 36 or 37 \|  \| \| 39. randomized controlled trial.mp. or exp randomized controlled trial/ \|  \| \| 40. exp clinical trial/ or exp "clinical trial (topic)"/ or clinical trial*.mp. or exp controlled study/ \|  \| \| 41. non-randomized trial.mp. \|  \| \| 42. random*.mp. \|  \| \| 43. exp placebo/ or placebo.mp. \|  \| \| 44. carisolv.mp. or exp glutamic acid/ \|  \| \| 45. glutamic acid.mp. \|  \| \| 46. exp leucine/ or leucine.mp. \|  \| \| 47. lysine.mp. or exp lysine/ \|  \| \| 48. 1 or 2 or 3 or 4 or 5 or 6 or 7 or 8 or 9 or 10 or 11 or 12 or 13 or 14 or 15 or 16 or 17 or 18 or 19 or 20 or 21 or 22 or 23 or 24 or 25 or 26 or 27 or 28 or 29 or 44 or 45 or 46 or 47 \|  \| \| 49. 39 or 40 or 41 or 42 or 43 \|  \| \| 50. 38 and 48 and 49 \|  \| \| 51. carie-care.mp. \|  \| \| 52. brix 3000.mp. \|  \| \| 53. 51 or 52 \|  \| \| 54. 1 or 2 or 3 or 4 or 5 or 6 or 7 or 8 or 9 or 10 or 11 or 12 or 13 or 14 or 15 or 16 or 17 or 18 or 19 or 20 or 21 or 22 or 23 or 24 or 25 or 26 or 27 or 28 or 29 or 44 or 45 or 46 or 47 or 51 or 52 \|  \| \| 55. 38 and 49 and 54 \|  \| |
| **Cochrane (for Cochrane Reviews and CENTRAL)**  #1 chlorhexidine  #2 “chlorhexidine digluconate”  #3 chlorhexamed  #4 “corsodyl ICI”  #5 “curasept ADS 220”  #6 eludril  #7 hexidine  #8 hibiclens  #9 hibident  #10 hibiscrub  #11 hibisol  #12 hibitane  #13 peridex  #14 “Perio Chip”  #15 avagard  #16 papain  #17 papacarie  #18 “antimicrobial photodynamic therapy”  #19 photochemotherap*  #20 “photodynamic therap*”  #21 ozone  #22 “ozone therapy”  #23 carisolv  #24 leucine  #25 lysine  #26 "glutamic acid"  #27 photostimulation  #28 photoirradiation  #29 photoactivation  #30 photobiomodulation  #31 PBMT  #32 PBM  #33 LLLT  #34 laser  #35 “light emitting diode”  #36 “low power laser irradiation”  #37 “laser biostimulation”  #38 #1 OR #2 OR #3 OR #4 OR #5 OR #6 OR #7 OR #8 OR #9 OR #10 OR #11 OR #12 OR #13 OR #14 OR #15 OR #16 OR #17 OR #18 OR #19 OR #20 OR #21 OR #22 OR #23 OR #24 OR #25 OR #26 OR #27 OR #28 OR #29 OR #30 OR #31 OR #32 OR #33 OR #34 OR #35 OR #36 OR #37  #39 "dental caries"  #40 caries  #41 "dental decay"  #42 "caries, dental"  #43 "decay, dental"  #44 "carious dentin*"  #45 "cavitated teeth"  #46 "cavitated tooth"  #47 #39 OR #40 OR #41 OR #42 OR #43 OR #44 OR #45 O4 #46  #48 "randomized controlled trial"  #49 "clinical trial"  #50 "non-randomized trial"  #51 random*  #52 placebo  #53 #48 OR #49 OR #50 OR #51 OR #52  #54 #38 AND #47 AND #53 |
| **Web of Science**  TS=((chlorhexidine OR chlorhexidine bigluconate OR chlorhexidine digluconate OR 1,1’-HBCB OR Chlorhexamed OR Hexidine OR papain OR papacarie OR antimicrobial photodynamic therapy OR photochemotherap* OR photodynamic therap* OR ozone OR ozone therap* OR carisolv OR leucine OR lysine OR glutamic acid OR photostimulation OR photoirradiation OR photoactivation OR photobiomodulation OR PBMT OR PBM OR LLLT OR laser OR light emitting diode OR red laser therap* OR infra-red laser therap* OR low intensity laser therap* OR light emitting diode OR red laser therap* OR infra-red laser therap* OR low intensity laser therap* OR low-intensity laser therap* OR low level laser therap* OR low-level laser therap* OR low level light therap* OR low-level light therap* OR low power laser therap* OR low-power laser therap* OR low power laser irradiation OR low-power laser irradiation OR photobiomodulation therap* OR laser biostimulation OR laser phototherapy*) AND (Dental Caries OR Caries OR Dental Decay OR Caries, Dental OR Decay, Dental or Carious Dentin* OR Cavitated Teeth OR Cavitated Tooth) AND (randomized controlled trial OR clinical trial OR non-randomized trial OR random* OR placebo)) |
| **SCOPUS**  TITLE-ABS-KEY (("chlorhexidine"  OR  "chlorhexidine bigluconate"  OR  "chlorhexidine digluconate" OR "1,1' HBCB"  OR  "chlorhexamed"  OR  "hexidine"  OR  "papain"  OR  "papacarie"  OR  "antimicrobial photodynamic therapy"  OR  "photochemotherap*"  OR  "photodynamic therap*"  OR  "photodynamic therap*"  OR  "ozone"  OR  "ozone therap*"  OR  "carisolv"  OR  "leucine"  OR  "lysine"  OR  "glutamic acid"  OR  "photostimulation"  OR  "photoirradiation"  OR  "photoactivation"  OR  "photobiomodulation"  OR  "PBMT"  OR  "PBM"  OR  "LLLT"  OR  "laser"  OR  "light emitting diode"  OR  "red laser therap*"  OR  "infra-red laser therap*"  OR  "low level light therap*"  OR  "low power laser therap*"  OR  "low power laser irradiation"  OR  "photobiomodulation therap*"  OR  "laser biostimulation"  OR  "laser phototherap*" ) AND  ("dental caries"  OR  "caries"  OR  "dental decay"  OR  "caries, dental"  OR  "decay, dental"  OR  "carious dentin*"  OR  "cavitated teeth"  OR  "cavitated tooth")  AND  ("randomized controlled trial"  OR  "clinical trial"  OR  "non-randomized trial"  OR  "random*"  OR  "placebo")) |
| **Dissertation Database (ProQuest Dissertation and Theses Database)**  (Dental Caries) AND (Ozone or Chlorhexidine or Photodynamic Therapy or Papacarie or Papain or Laser or Carisolv or Leucine or Lysine or Glutamic Acid) |
| **The WHO International Clinical Trials Registry Plataform**  Dental Caries and Ozone  Dental Caries and Chlorhexidine  Dental Caries and Photodynamic Therapy  Dental Caries and Papacarie  Dental Caries and Papain  Dental Caries and Laser  Dental Caries and Carisolv |

**Table B.** Reasons of studies excluded in full text screening.

| **Reasons** | **Studies** |
| --- | --- |
| Analysis only in the follow-up | (1) |
| Eligible with no results | (2-7) |
| In vitro/In situ or Review | (8-10) |
| No microbiological analysis | (11-14) |
| Not Available | (15, 16) |
| Protocols of clinical trials already included | (17-21) |
| Not compatible with our inclusion criteria | (22-32) |
| Studies with single treatment arm | (33-35) |

**Statistical Methods**

***Effect measure***

The primary and secondary outcomes are measured as the mean and standard deviation of counts out of the total randomized participants in each arm of every trial. As an effect measure, we considered the ratio of ratio of means (RoRoM), that is the ratio of ratio of post-treatment to baseline means between two compared interventions (36). Using the natural logarithmic scale, log RoRoM has similar desirable statistical properties with the log RoM and by extent with the log odds ratio (37). RoRoM is a plausible effect measure since the mean value of the comparing arms in each trial is positive by definition, and there are no zero counts observed. Therefore, the logarithm of RoRoM is always defined. In the present study, a positive log RoRoM (or RoRoM > 1) favors the second intervention in the comparison, a negative log RoRoM (or RoRoM < 1) favors the first intervention in the comparison, and log RoRoM equal to zero (or RoRoM = 1) indicates no association between the compared interventions and the investigated outcome. Note that RoRoM is a unitless effect measure (36). With $y_{ik}^{B}$, and $y_{ik}^{F}$, we indicate the average counts at baseline and post-treatment, respectively, in arm $k$ ($k=1,2,\ldots,a_{i}$ with $a_{i}$ being the number of arms in trial $i$) of trial $i$, and with $s_{ik}^{B}$, and $s_{ik}^{F}$, we indicate the corresponding standard deviation of counts at baseline and post-treatment. Then, the RoRoM between arm $k$ and baseline arm (i.e. $k=1$) of trial $i$ is calculated as follows,

$${RoRoM}_{i}=\frac{\frac{y_{ik}^{F}}{y_{ik}^{B}}}{\frac{y_{i1}^{F}}{y_{i1}^{B}}}=\frac{y_{ik}^{F}y_{i1}^{B}}{y_{i1}^{F}y_{ik}^{B}}$$

or

| $ln\left( {RoRoM}_{i} \right)=\left[ ln\left( y_{ik}^{F} \right)-ln\left( y_{i1}^{F} \right) \right]-\left[ ln\left( y_{ik}^{B} \right)-ln\left( y_{i1}^{B} \right) \right]$ | (1) |
| --- | --- |

in the logarithmic scale. The variance of log RoRoM can be obtained straightforwardly using the delta method (limited to the first-order large-sample Taylor series approximation) (15),

$$v\left[ ln\left( {RoRoM}_{i} \right) \right]=\left[ \left( \frac{s_{ik}^{F}}{y_{ik}^{F}} \right)^{2}\frac{1}{n_{ik}^{F}}+\left( \frac{s_{i1}^{F}}{y_{i1}^{F}} \right)^{2}\frac{1}{n_{i1}^{F}} \right]+\left[ \left( \frac{s_{ik}^{B}}{y_{ik}^{B}} \right)^{2}\frac{1}{n_{ik}^{B}}+\left( \frac{s_{i1}^{B}}{y_{i1}^{B}} \right)^{2}\frac{1}{n_{i1}^{B}} \right]$$

$$-2\rho\left( \frac{s_{ik}^{F}s_{ik}^{B}}{y_{ik}^{F}y_{ik}^{B}}\frac{1}{\sqrt{n_{ik}^{F}n_{ik}^{B}}}+\frac{s_{i1}^{F}s_{i1}^{B}}{y_{i1}^{F}y_{i1}^{B}}\frac{1}{\sqrt{n_{i1}^{F}n_{i1}^{B}}} \right)$$

with $\rho$ being the correlation coefficient which is assumed to be fixed within and across trials, and $n_{ik}^{B}$ and $n_{ik}^{F}$ being the number of participants at baseline and post-treatment in arm $k$ of trial $i$. The correlation is necessary since $y_{ik}^{F}$ and $y_{ik}^{B}$ ($k=1,2,\ldots,a_{i}$) are correlated for referring to the same participants receiving arm $k$ in trial $i$. Since we have no information on $\rho$, we make assumptions about a clinically plausible value for $\rho$ above 0.5; otherwise, an analysis using the change from baseline will be less precise than the post-intervention analysis (38). We assumed $\rho$ equal 0.8 to be a clinically plausible value for the present study. As a sensitivity analysis, we assumed $\rho$ equal to 0.6. Then, the 95% confidence interval (CI) of the log RoRoM between arm $k$ and baseline arm of trial $i$ is calculated as follows,

$$95\% CI=ln\left( \mathrm{Ro}{RoM}_{i} \right)\pm1.96\sqrt{v\left[ ln\left( \mathrm{Ro}{RoM}_{i} \right) \right]}$$

or

| $95\% CI=exp\left\{ ln\left( {\mathrm{Ro}RoM}_{i} \right)\pm1.96\sqrt{v\left[ ln\left( {\mathrm{Ro}RoM}_{i} \right) \right]} \right\}$ | (2) |
| --- | --- |

at the exponential scale (back-transformation). A 95% CI that includes the null value (i.e., zero at the log scale, one at the original scale) implies weak evidence for the effectiveness of the compared interventions.

***Analysis performed***

We created a panel of forest plots on the within-trial estimated log RoRoMs for each observed pairwise comparison in the investigated outcomes. We used different line-type and line-colours to depict the trial-design (RCT versus NRCT), method of bacterial counting (CFU versus q-PCR) and the risk of bias (some concerns versus high risk) as judged using the Cochrane RoB-2 and ROBINS-I tools for RCT and NRCT, respectively. We used the equations (1) and (2) to calculate the trial-specific log RoRoMs and 95% CIs for all possible pairwise comparisons in a multi-arm trial. We used the R-package *ggplot2* to obtain all figures (39) and the R-package *pcnetmeta* (40) to create the network plots.

***Addressing missing participant outcome data***

Some trials had participant losses as the number of participants who completed the trial was smaller than the number of randomized participants at baseline. We used the pattern-mixture model to simultaneously analyze aggregate observed and missing outcome data at post-treatment in each trial's arm. This model distinguishes the participants to those completing and those leaving the trial prematurely,

| $E\left( y_{ik}^{F} \right)=\theta_{ik}^{F}=\theta_{ik}^{o,F}\left( 1-q_{ik} \right)+\theta_{ik}^{m,F}q_{ik}$ | (3) |
| --- | --- |

where $\theta_{ik}^{F}$ is the underlying mean of the outcome at post-treatment given the randomized participants in arm $k$ in trial $i$, $\theta_{ik}^{o,F}$ and $\theta_{ik}^{m,F}$ are the underlying means of the outcome at post-treatment given the completers and missing participants, respectively, and $q_{ik}$ is the probability of missing participants. We have information on the mean and standard deviation of the outcome at post-treatment among the completers only, namely, $y_{ik}^{o,F}$ and $s_{ik}^{o,F}$. For those participants who left the trial early, we need to make a clinically plausible assumption about their outcome relative to the completers' outcome. We use the informative missingness ratio of means (IMRoM) parameter (41) to quantify the departure from the Missing At Random (MAR) assumption, which is the recommended starting point. The IMRoM is intuitively related to the RoRoM, and it is defined for each arm of every trial as follows (41),

$$e^{\delta_{ik}}=\frac{\theta_{ik}^{m,F}}{\theta_{ik}^{o,F}}$$

or

$$\delta_{ik}=ln\left( \theta_{ik}^{m,F} \right)-ln\left( \theta_{ik}^{o,F} \right)$$

on the logarithmic scale. Since $\delta_{ik}$ cannot be estimated directly from the data, we may assign a normal distribution on $\delta_{ik}$ with a plausible value for the mean and the variance to reflect our corresponding prior belief and uncertainty about the missingness mechanism on average in arm $k$ of trial $i$ (41),

$$\delta_{ik}\sim N\left( \Delta_{ik},\sigma_{ik}^{2} \right).$$

Then, a positive $\Delta_{ik}$ indicates a larger outcome on average given the missing participants as compared to the completers, a negative $\Delta_{ik}$ indicates the opposite, and $\Delta_{ik}$ equal zero reflects the MAR assumption. Following Mavridis (41), we consider $\sigma_{ik}^{2}={0.2}^{2}$ to be a plausible value that reflects a moderate uncertainty about log IMRoM. By replacing $\theta_{ik}^{m,F}=e^{\delta_{ik}}\theta_{ik}^{o,F}$ in equation (3), we obtain,

| $\theta_{ik}^{F}=\theta_{ik}^{o,F}\left[ 1-q_{ik}\left( 1-e^{\delta_{ik}} \right) \right]$ | (4) |
| --- | --- |

Furthermore, by replacing $\delta_{ik}=\Delta_{ik}$, $\hat{q}_{ik}=\frac{m_{ik}}{n_{ik}}$ and $\hat{\theta}_{ik}^{o,F}=y_{ik}^{o,F}$ in equation (4) and then, incorporating in equation (1), we obtain the log RoRoM adjusted for missing participants,

$$ln\left( \hat{RoRoM}_{i} \right)=\left[ ln\left( \hat{\theta}_{ik}^{F} \right)-ln\left( \hat{\theta}_{i1}^{F} \right) \right]-\left[ ln\left( y_{ik}^{B} \right)-ln\left( y_{i1}^{B} \right) \right]$$

The variance of $ln\left( \hat{RoRoM}_{i} \right)$ is the combination of the variance arising from the observed data and the variance arising from the log IMRoM – both estimated using Taylor approximation as described in Mavridis et al. (41).

**Results Report**

***Appendix figures Primary Outcome: Total Number of Bacteria***


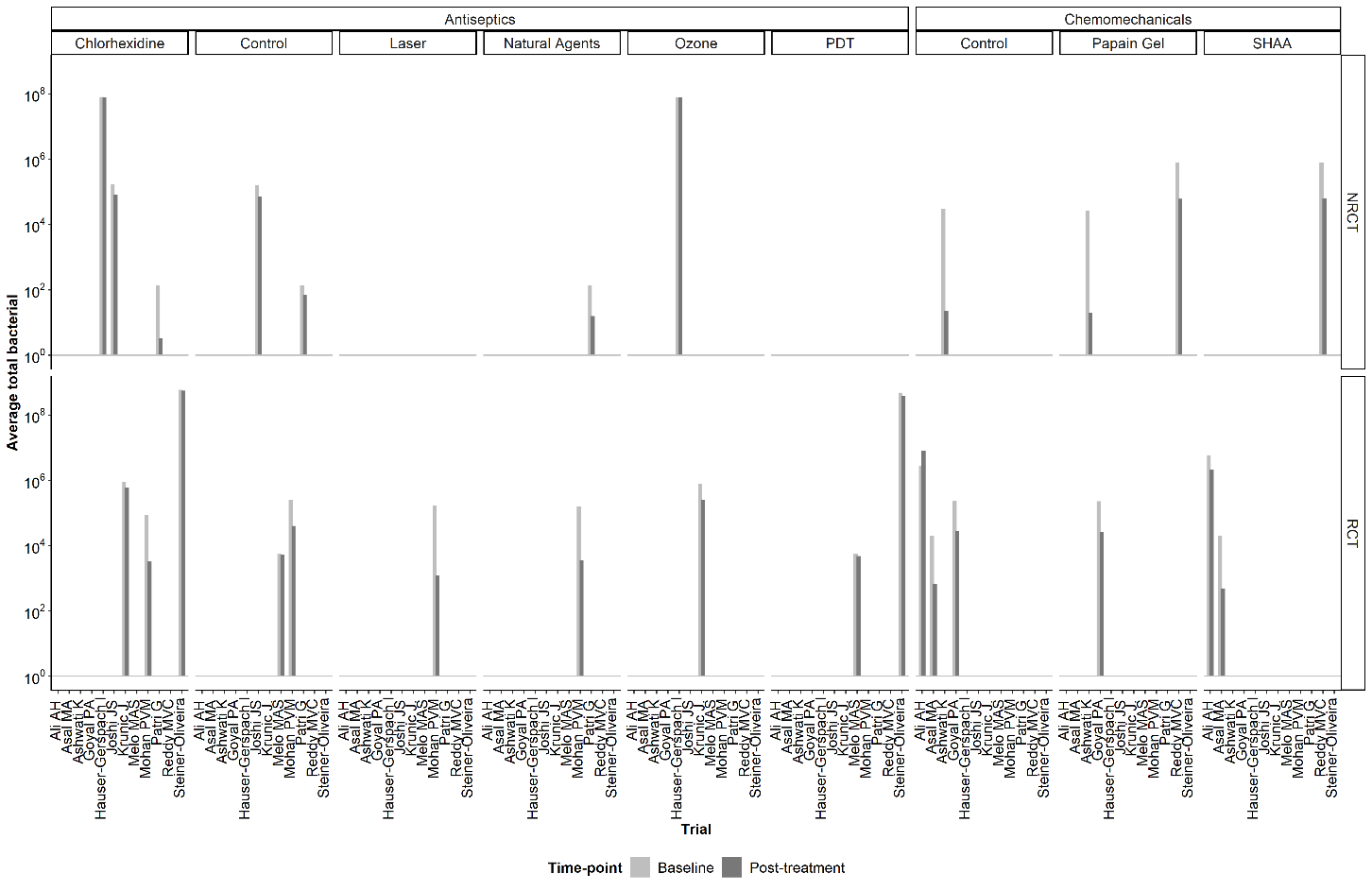


**S1 Figure.** A panel bar plots on the average total bacterial of each intervention arm of every trial at baseline and post-intervention, separately for RCT and NRCT.


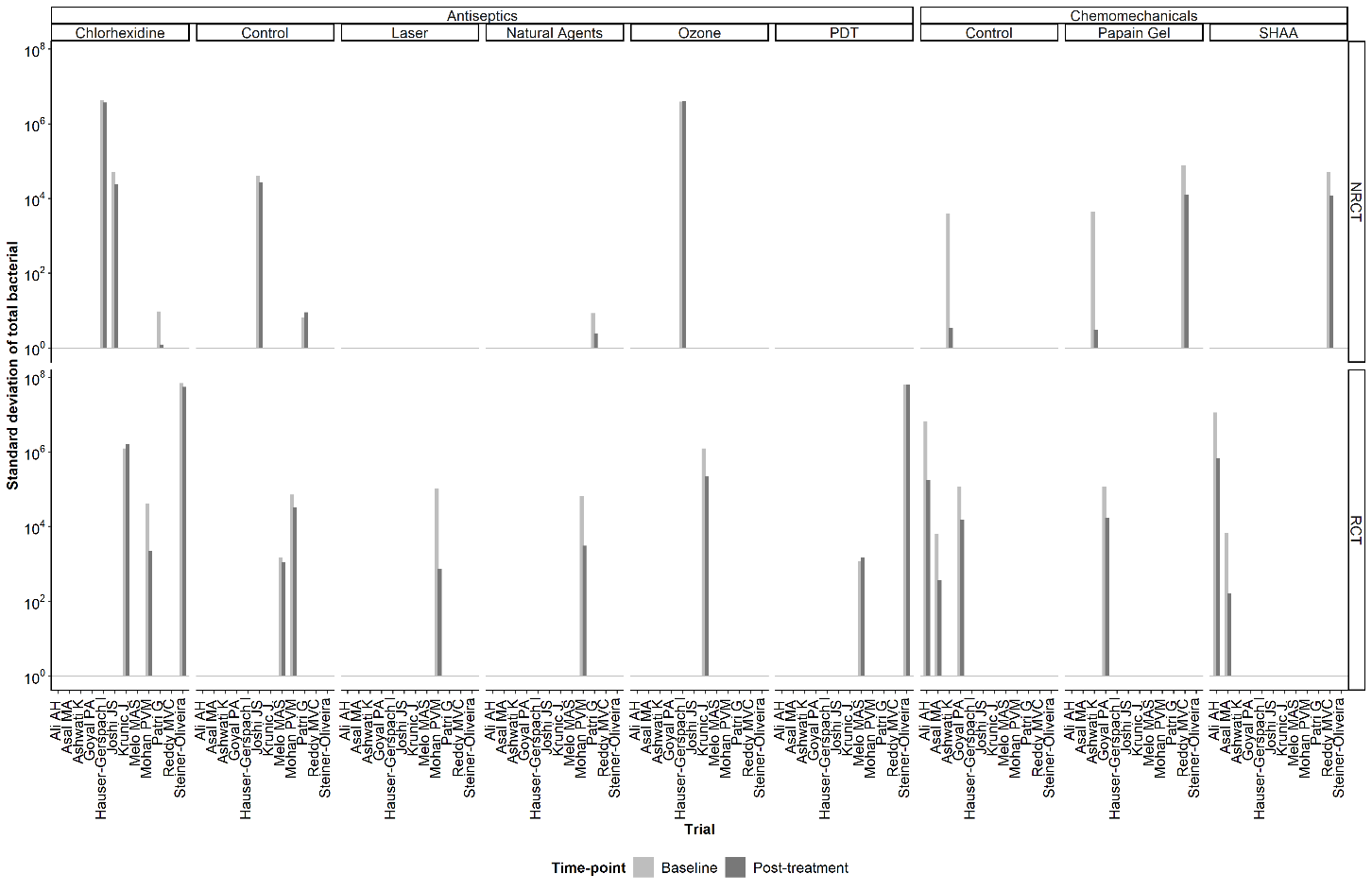


**S2 Figure.** A panel bar plots on the standard deviation of total bacterial of each intervention arm of every trial at baseline and post-intervention, separately for RCT and NRCT.


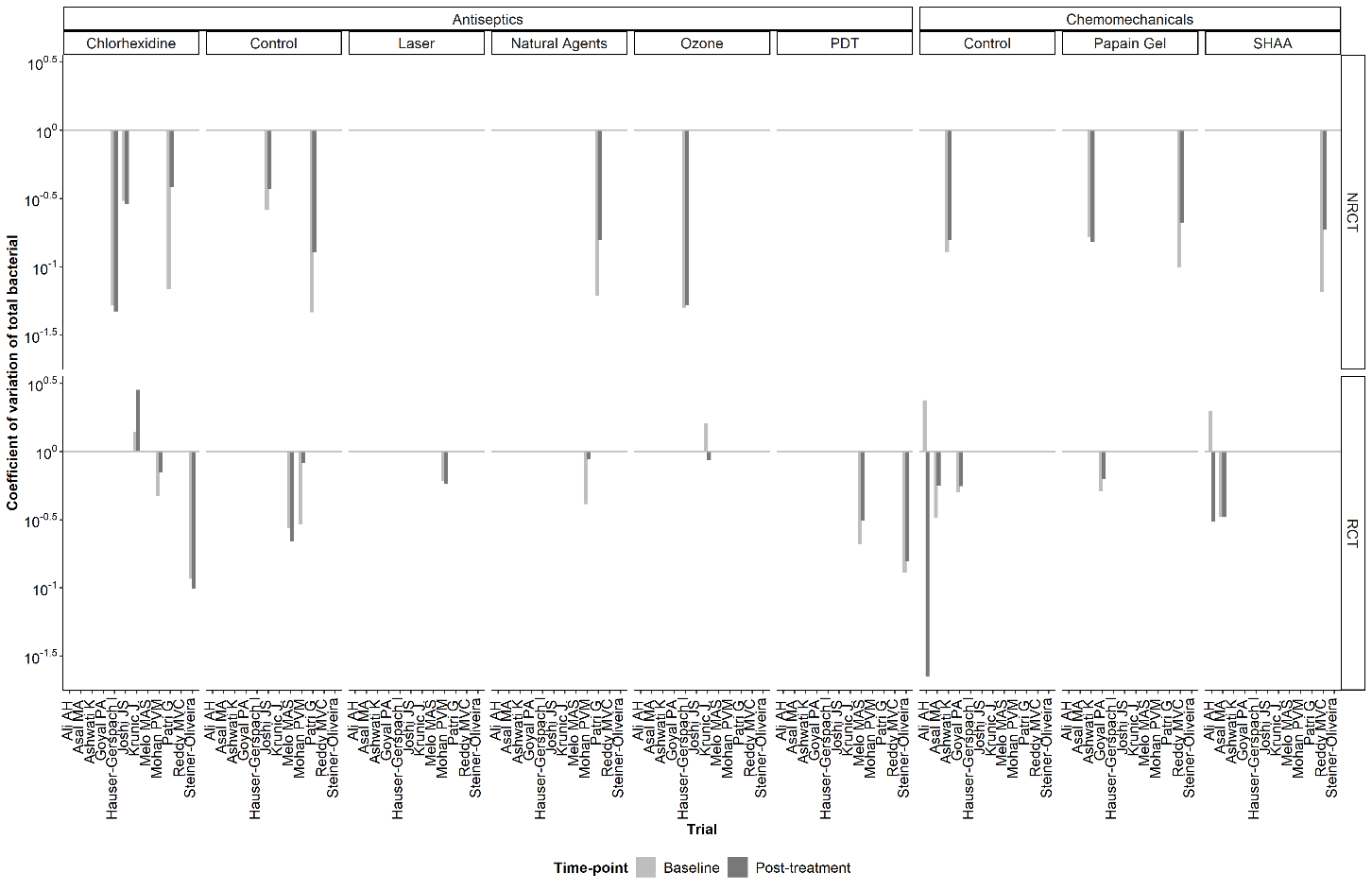


**S3 Figure.** A panel bar plots on the coefficient of variation (i.e., the ratio of the standard deviation to mean) of each intervention arm of every trial at baseline and post-intervention, separately for RCT and NRCT.

**Table C.** Ratio of ratio of means and 95% confidence interval of total bacterial count.

| **ID** | **Trial** | **Comparison** | **RoRoM (SE)** | **95% CI** |
| --- | --- | --- | --- | --- |
| 1 | (42) | Chlorhexidine vs Ozone | 2.05 (0.44) | 0.87 – 4.84 |
| 2 | (43) | Papain Gel vs Control | 0.98 (0.10) | 0.81 – 1.20 |
| 3 | (44) | Chlorhexidine vs Control | 0.23 (1.07) | 0.03 – 1.83 |
| 3 | (44) | Laser vs Chlorhexidine | 0.20 (3.07) | 0.001 – 78.30 |
| 3 | (44) | Natural Agents vs Chlorhexidine | 0.61 (1.45) | 0.04 – 10.35 |
| 3 | (44) | Laser vs Control | 0.04 (2.88) | 0.0002 – 12.15 |
| 3 | (44) | Natural Agents vs Control | 0.14 (0.98) | *0.02 – 0.93*^1^ |
| 3 | (44) | Natural Agents vs Laser | 3.09 (3.04) | Not plausible^2^ |
| 4 | (45) | PDT vs Control | 0.89 (0.06) | 0.80 – 1.00 |
| 5 | (46) | Chlorhexidine vs PDT | 1.14 (0.03) | *1.08 – 1.21* |
| 6 | (47) | SHAA vs Control | 0.13 (0.37) | *0.06 – 0.26* |
| 7 | (48) | Chlorhexidine vs Control | 0.05 (0.45) | *0.02 – 0.11* |
| 7 | (48) | Natural Agents vs Chlorhexidine | 4.77 (0.46) | *1.95 – 11.66* |
| 7 | (48) | Natural Agents vs Control | 0.22 (0.06) | *0.20 – 0.25* |
| 8 | (49) | Chlorhexidine vs Ozone | 0.98 (0.01) | 0.97 – 1.00 |
| 9 | (50) | SHAA vs Papain Gel | 1.03 (0.08) | 0.88 – 1.21 |
| 10 | (51) | Chlorhexidine vs Control | 1.09 (0.05) | 0.99 – 1.20 |
| 11 | (52) | Papain Gel vs Control | 1.01 (0.03) | 0.95 – 1.07 |
| 12 | (53) | SHAA versus Control | 0.71 (0.07) | 0.62 – 0.82 |

CI: confidence interval; PDT: Photodynamic Therapy; RoRoM: ratio of ratio of means; SE: standard error; SHAA: Sodium Hypochlorite and Amino Acids. ^1^Results in italic indicate strong evidence in favour of the first (RoRoM < 1) or second intervention in the comparison (RoRoM> 1). ^2^A considerable standard error results in implausibly wide 95% confidence intervals that are not interpretable.


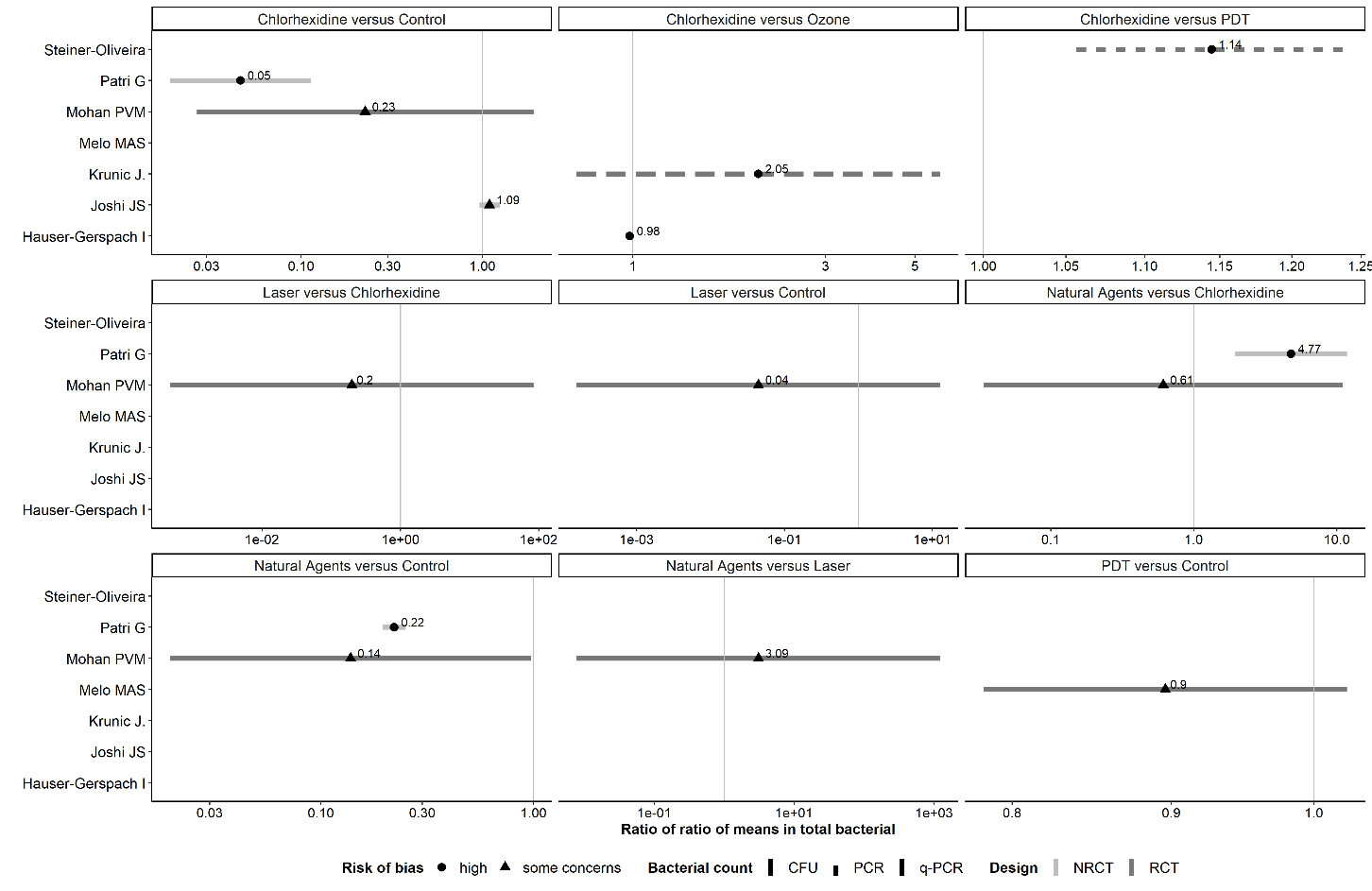


**S4 Figure.** A panel of forest plots on the within-trial ratio of means (RoM) in the change from baseline of total bacterial for antiseptics. Use of correlation coefficient equal to 0.6 as a sensitivity analysis. The vertical grey line implies no difference in the compared interventions. RoM above one favours the second intervention in the comparison, and RoM below one favours the first intervention in the comparison. The trial-design (randomised versus non-randomised-controlled trial) is indicated with different line-colours, the risk of bias (high versus some concerns) is indicated with different point shape, and the method for the bacterial count (CFU versus q-PCR) is indicated with different line-type.

**
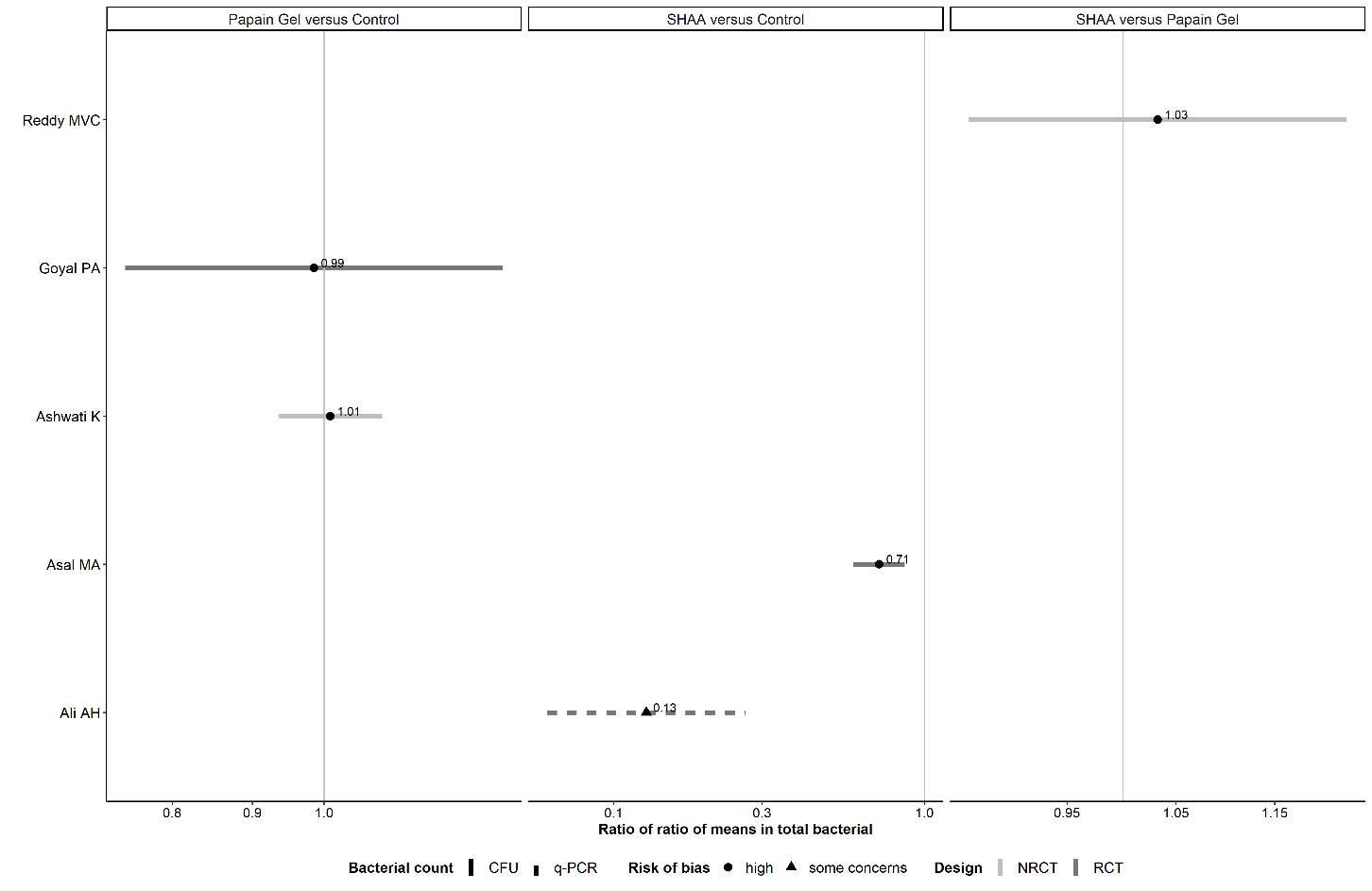
**

**S5 Figure.** A panel of forest plots on the within-trial ratio of means (RoM) in the change from baseline of total bacterial for chemomechanical methods. Use of correlation coefficient equal to 0.6 as a sensitivity analysis. The vertical grey line implies no difference in the compared interventions. RoM above one favours the second intervention in the comparison, and RoM below one favours the first intervention in the comparison. The trial-design (randomised versus non-randomised-controlled trial) is indicated with different line-colours, the risk of bias (high versus some concerns) is indicated with different point shape, and the method for the bacterial count (CFU versus q-PCR) is indicated with different line-type.

***Secondary Outcomes: Total number of Lactobacillus and Streptococcus mutans***


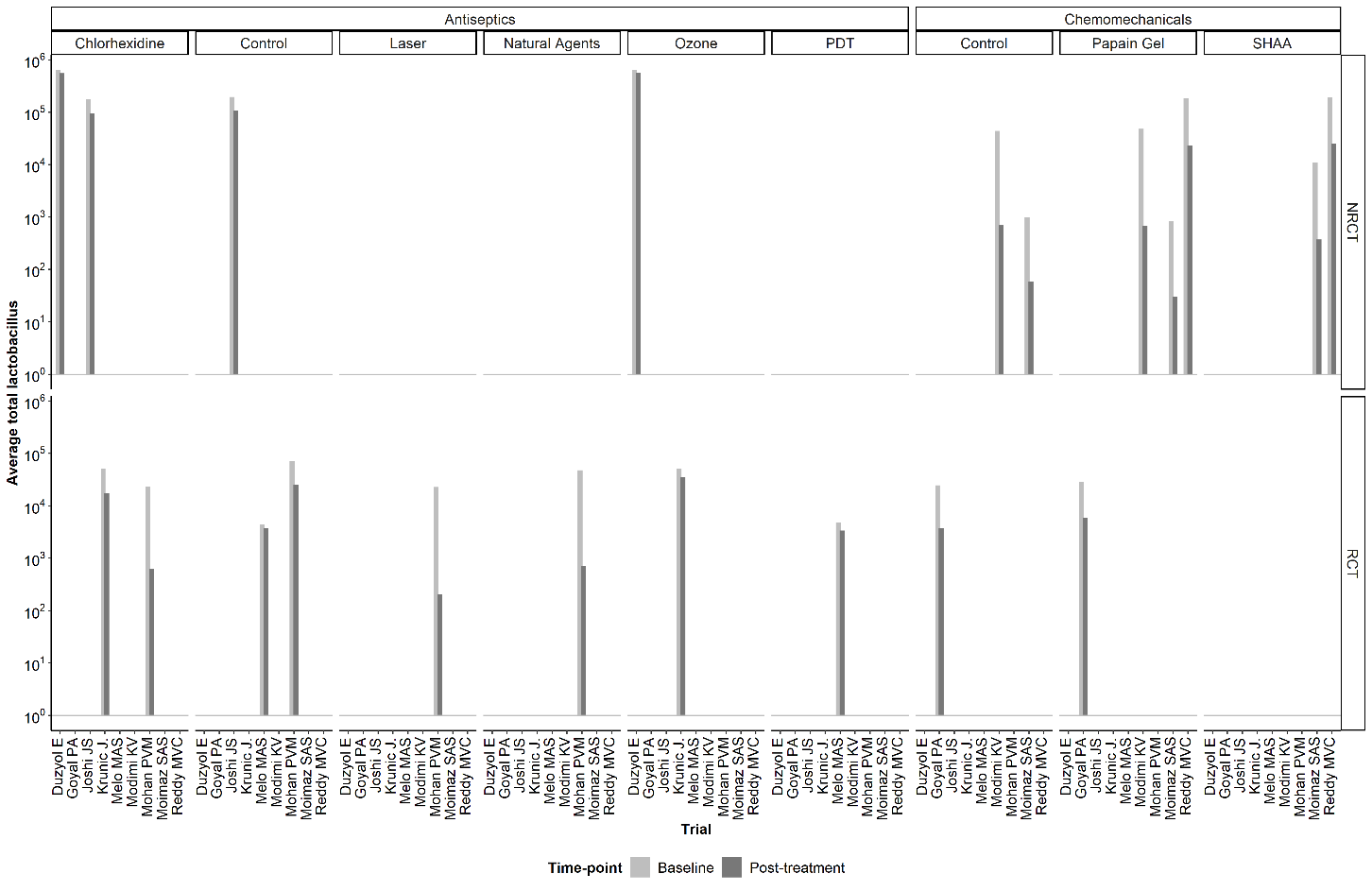


**S6 Figure.** A panel bar plots on the average total *Lactobacillus* of each intervention arm of every trial at baseline and post-intervention, separately for RCT and NRCT.


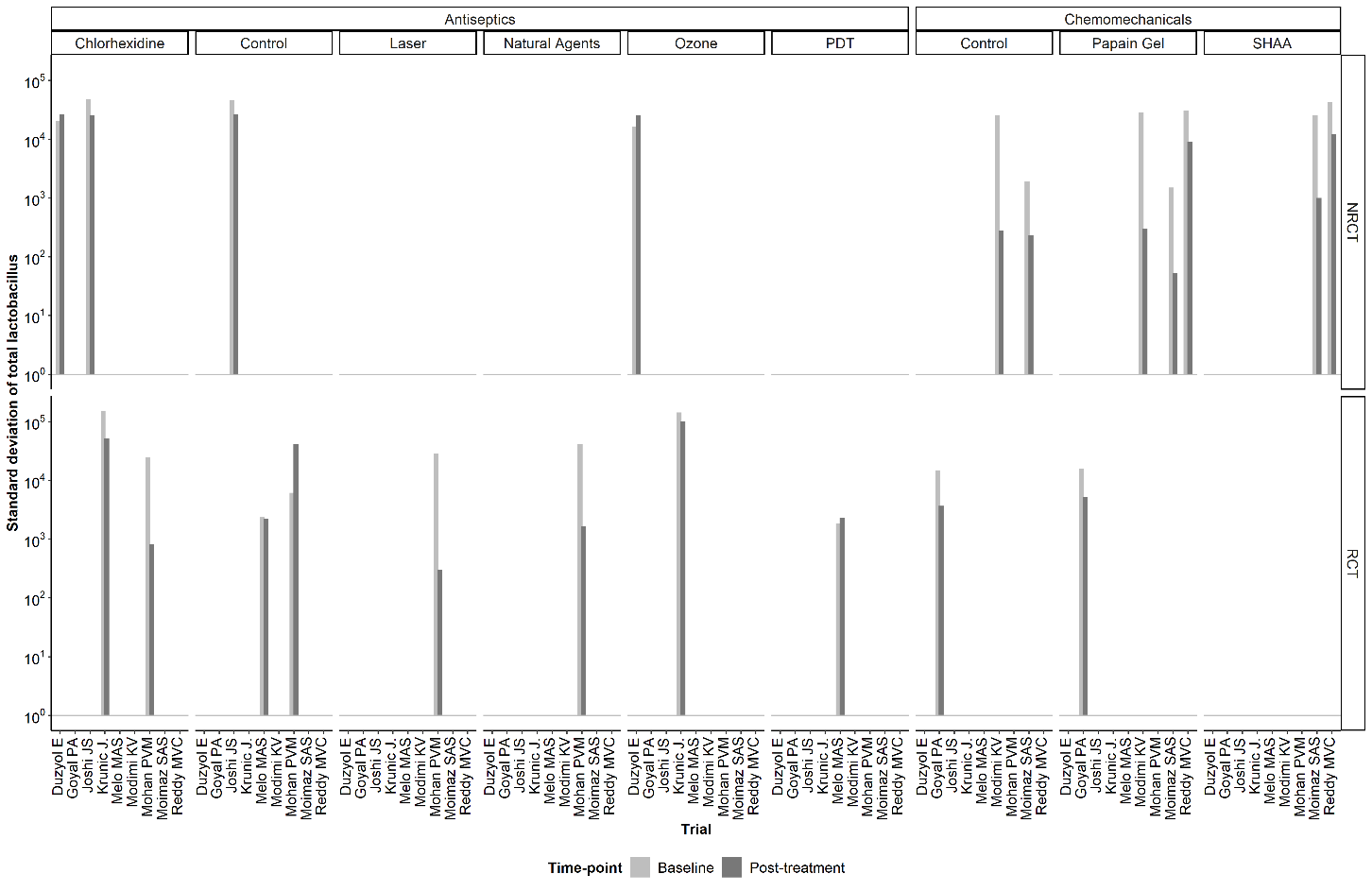


**S7 Figure.** A panel bar plots on the standard deviation of total *Lactobacillus* of each intervention arm of every trial at baseline and post-intervention, separately for RCT and NRCT.


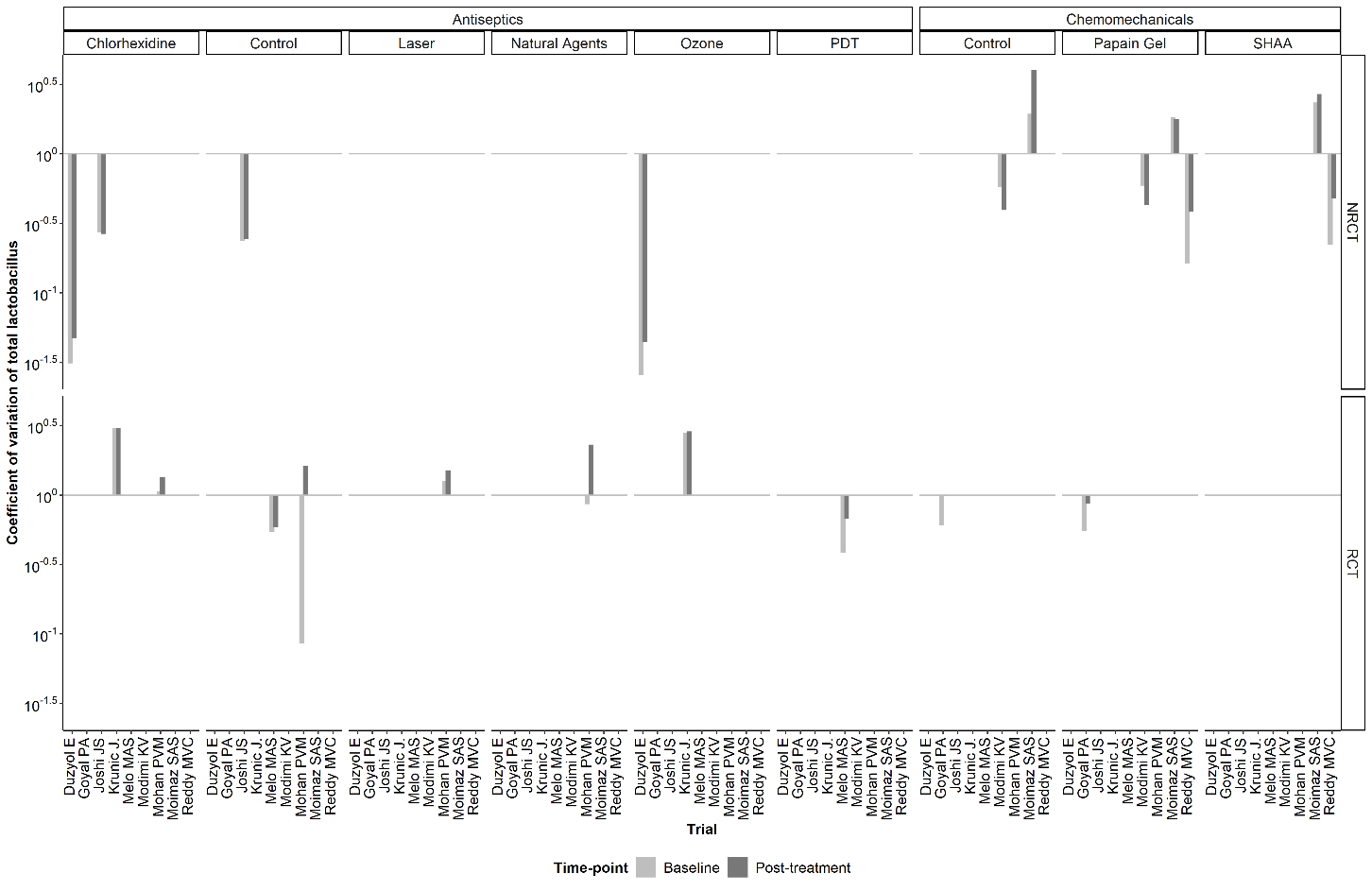


**S8 Figure.** A panel bar plots on the coefficient of variation for *Lactobacillus* (i.e. the ratio of the standard deviation to mean) of each intervention arm of every trial at baseline and post-intervention, separately for RCT and NRCT.


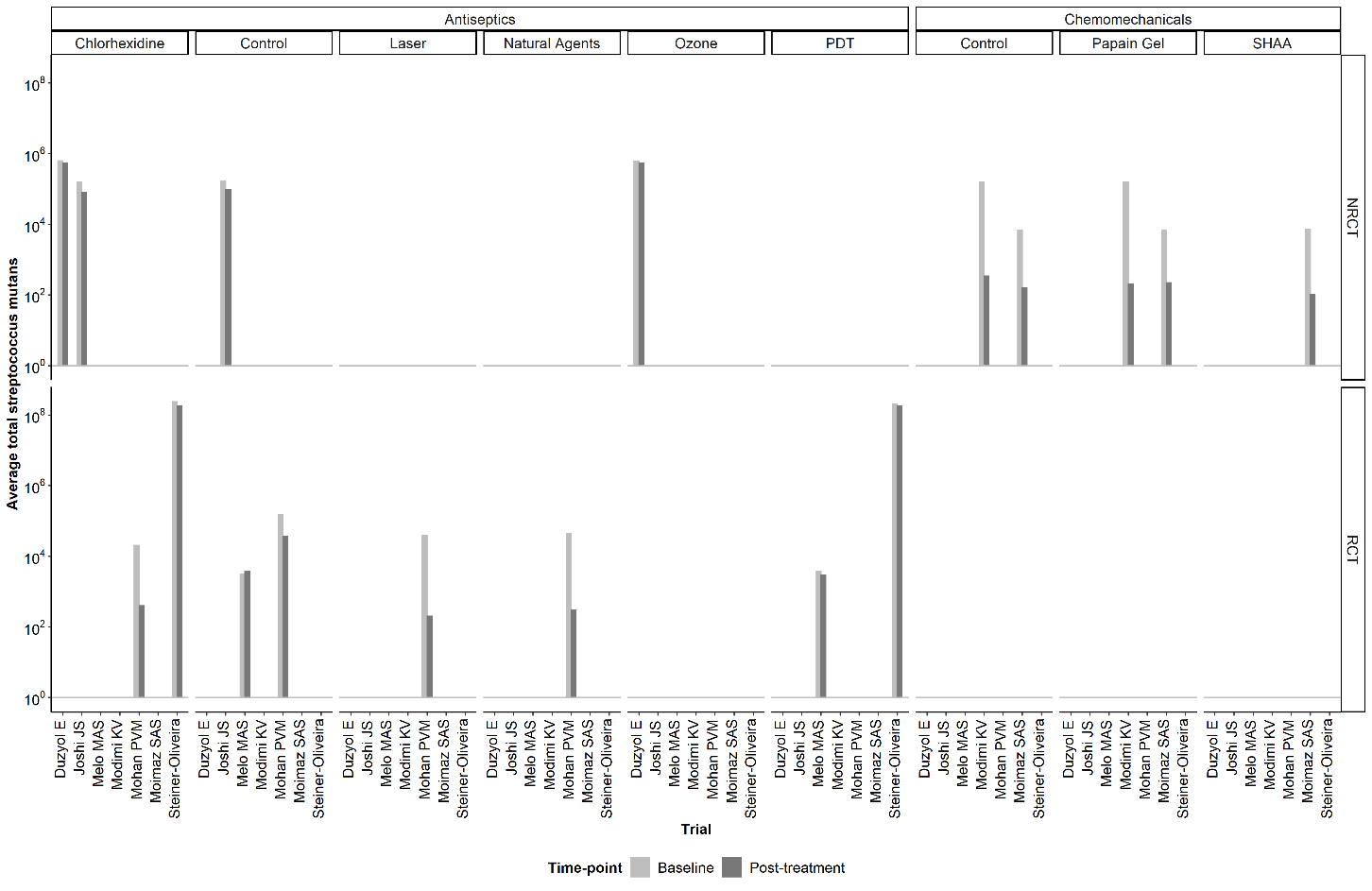


**S9 Figure.** A panel bar plots the average total *Streptococcus mutans* of each intervention arm of every trial at baseline and post-intervention, separately for RCT and NRCT.


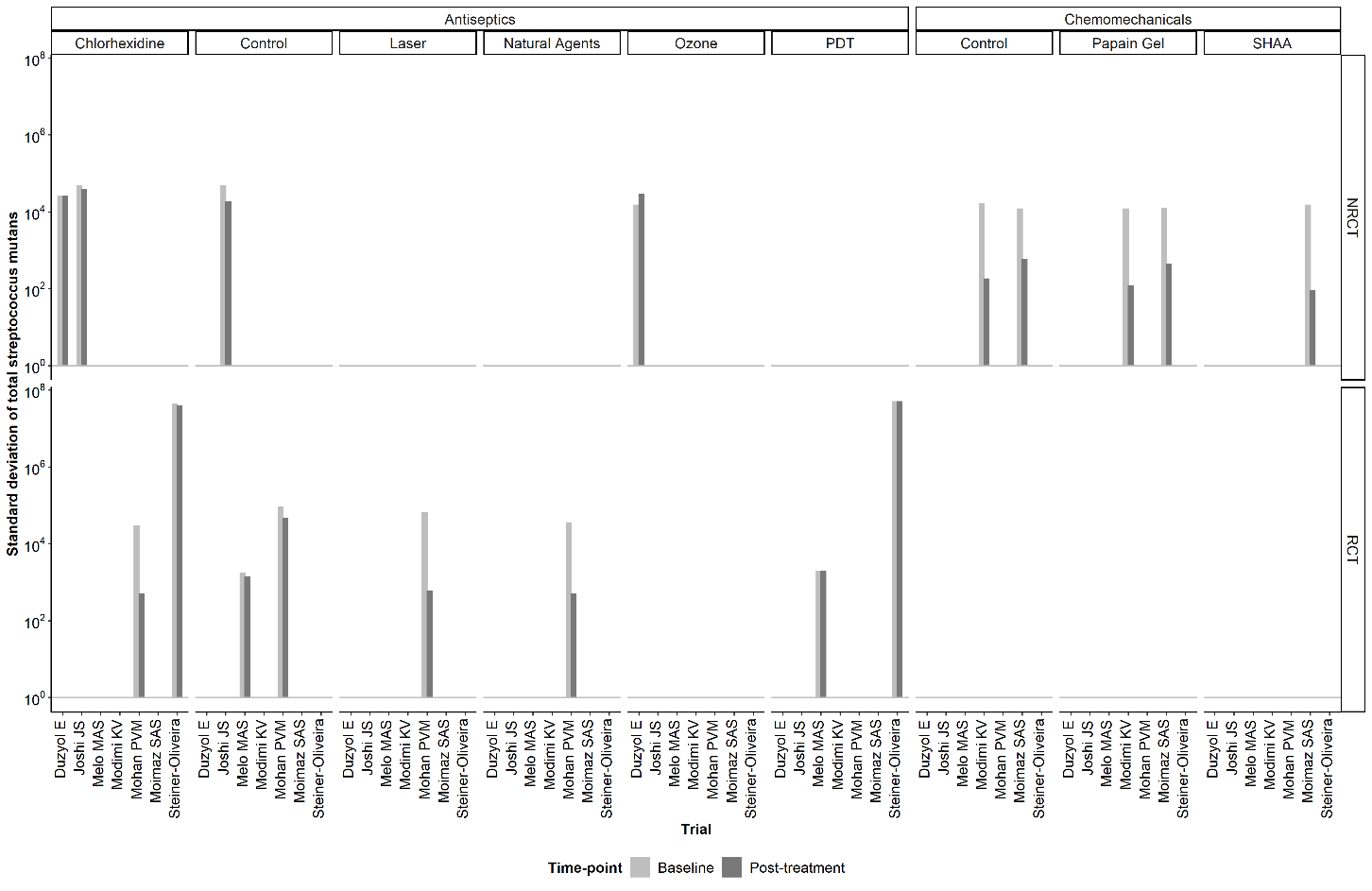


**S10 Figure.** A panel bar plots on the standard deviation of total *Streptococcus mutans* of each intervention arm of every trial at baseline and post-intervention, separately for RCT and NRCT.


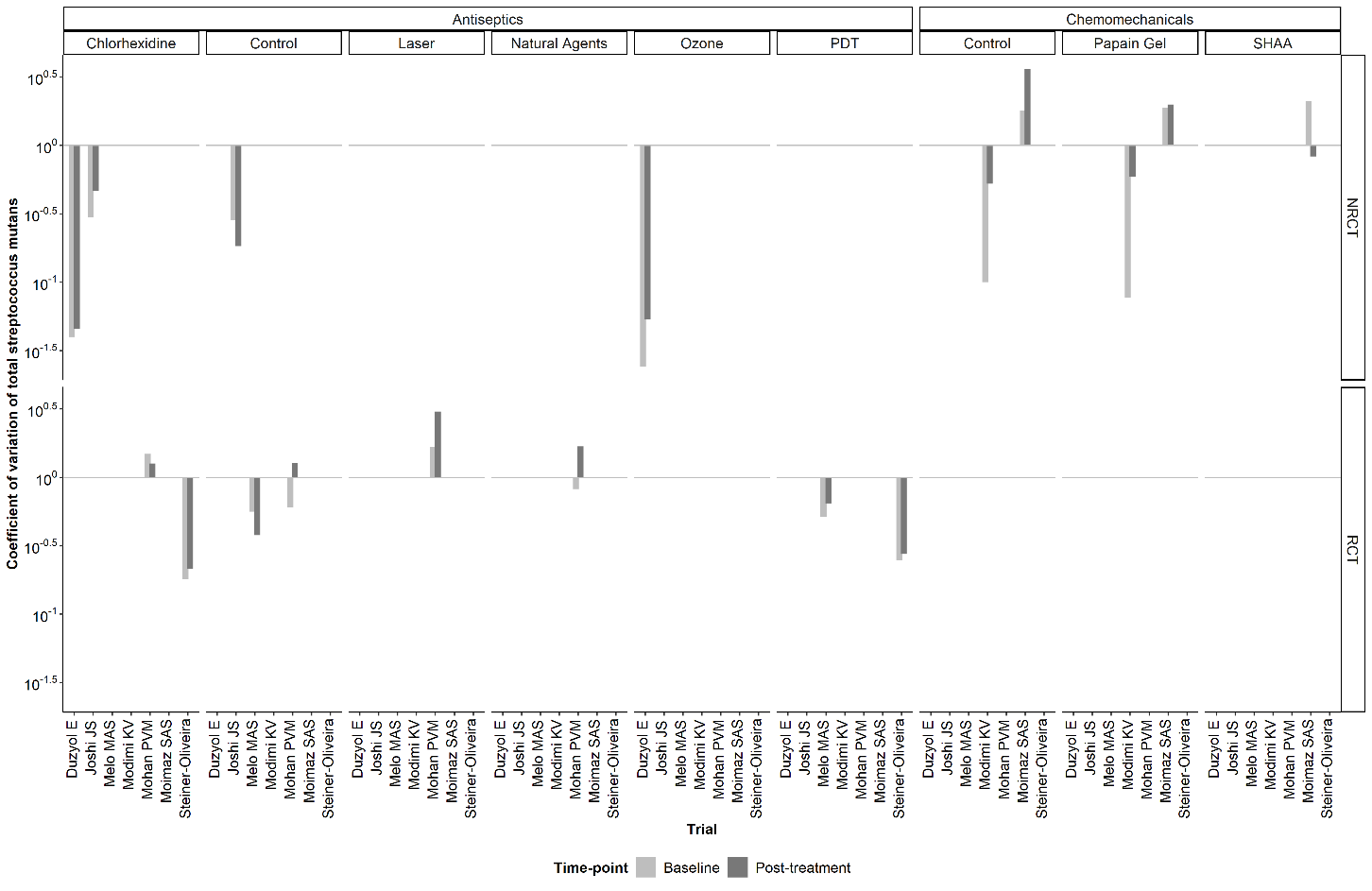


**S11 Figure.** A panel bar plots on the coefficient of variation of *Streptococcus mutans* (i.e., the ratio of the standard deviation to mean) of each intervention arm of every trial at baseline and post-intervention, separately for RCT and NRCT.

**Table D.** Ratio of ratio of means and 95% confidence interval of total *Lactobacillus.*

| **ID** | **Trial** | **Comparison** | **RoRoM (SE)** | **95% CI** |
| --- | --- | --- | --- | --- |
| 1 | Krunic J (42) | Chlorhexidine vs ozone | 0.49 (0.53) | 0.17 – 1.37 |
| 2 | Goyal PA (43) | Papain Gel vs Control | 1.36 (0.17) | 0.98 – 1.88 |
| 3 | Mohan PVM (44) | Chlorhexidine vs Control | 0.07 (7.27) | Not plausible^1^ |
| 3 | Mohan PVM (44) | Laser vs Chlorhexidine | 0.34 (23.32) | Not plausible |
| 3 | Mohan PVM (44) | Natural Agents vs Chlorhexidine | 0.57 (9.58) | Not plausible |
| 3 | Mohan PVM (44) | Laser vs Control | 0.02 (22.16) | Not plausible |
| 3 | Mohan PVM (44) | Natural Agents vs Control | 0.04 (6.24) | Not plausible |
| 3 | Mohan PVM (44) | Natural Agents vs Laser | 1.68 (23.02) | Not plausible |
| 4 | Melo MAS (45) | PDT vs Control | 0.83 (0.10) | 0.68 – 1.00 |
| 5 | Reddy MVC (50) | SHAA vs Papain Gel | 1.03 (0.13) | 0.80 – 1.33 |
| 6 | Joshi JS (51) | Chlorhexidine vs Control | 0.98 (0.04) | 0.91 – 1.05 |
| 7 | Modimi KV (54) | Papain Gel vs Control | 0.90 (0.09) | 0.75 – 1.08 |
| 8 | Moimaz SAS (55) | SHAA vs Control | 0.58 (1.38) | 0.04 – 8.57 |
| 8 | Moimaz SAS (55) | Papain Gel vs SHAA | 1.08 (3.99) | Not plausible |
| 8 | Moimaz SAS (55) | Papain Gel vs Control | 0.62 (4.19) | Not plausible |
| 9 | Duzyol E (56) | Chlorhexidine versus Ozone | 0.95 (0.01) | 0.93 – 0.97 |

CI: confidence interval; PDT: Photodynamic Therapy; RoRoM: ratio of ratio of means in change from baseline; SE: standard error; SHAA: Sodium Hypochlorite and Amino Acids.

^1^A considerable standard error results in implausibly wide 95% confidence intervals that are not interpretable.


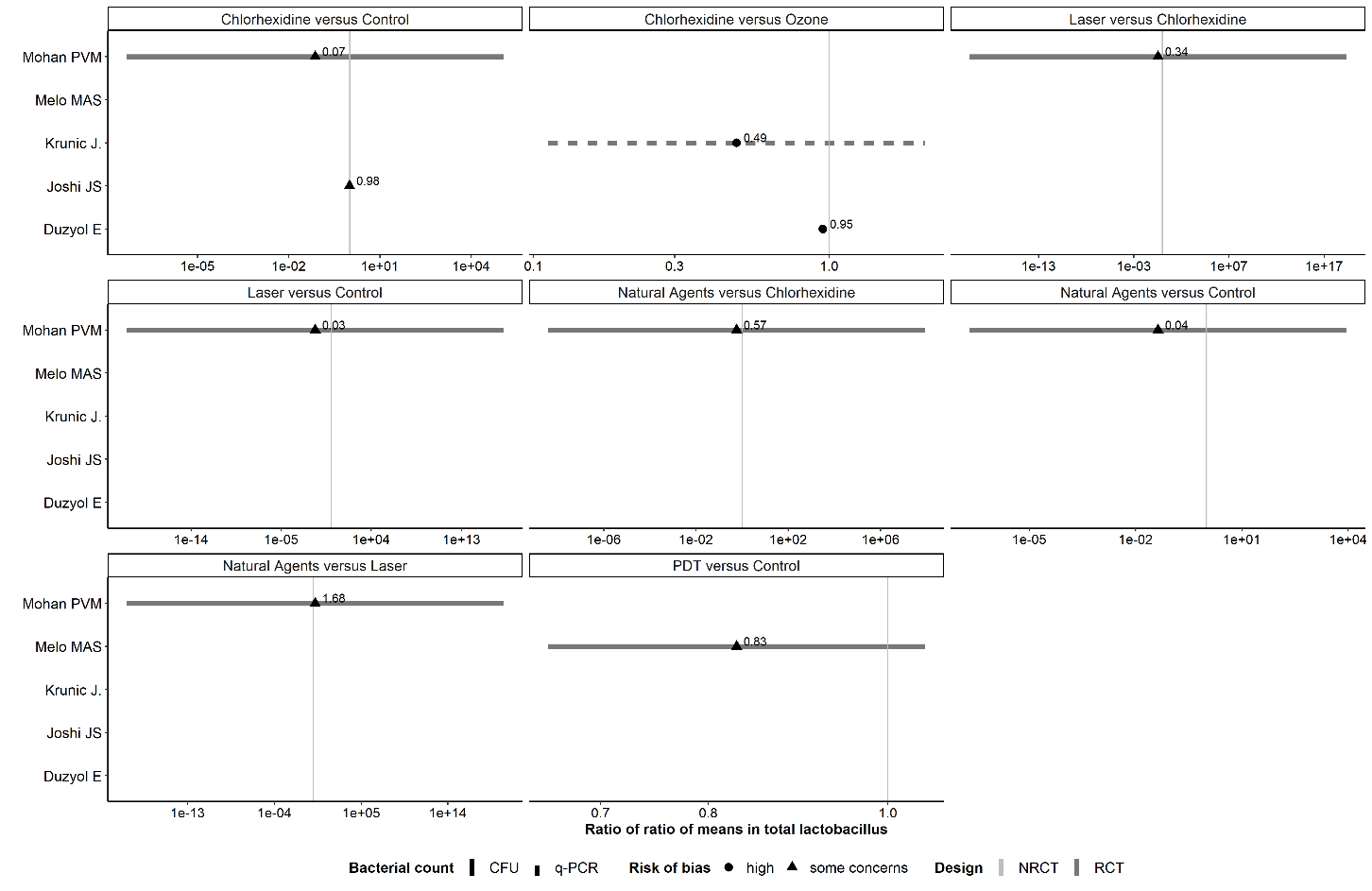


**S12 Figure.** A panel of forest plots on the within-trial ratio of means (RoM) in the change from baseline of total *Lactobacillus* for antiseptics. Use of correlation coefficient equal to 0.6 as a sensitivity analysis. The vertical grey line implies no difference in the compared interventions. RoM above one favours the second intervention in the comparison, and RoM below one favours the first intervention in the comparison. The trial-design (randomised versus non-randomised-controlled trial) is indicated with different line-colours, the risk of bias (high versus some concerns) is indicated with different point shape, and the method for the bacterial count (CFU versus q-PCR) is indicated with different line-type.


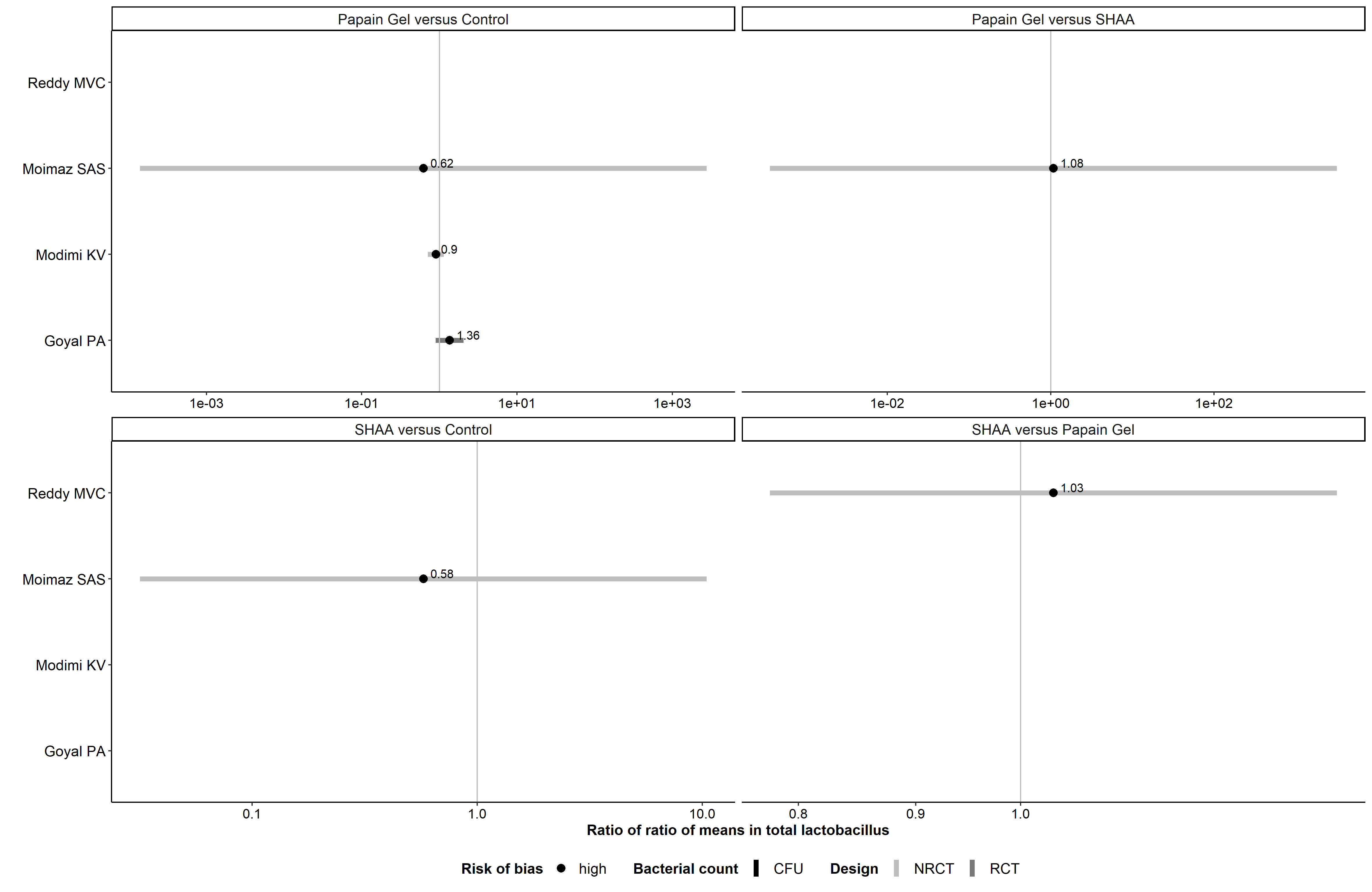


**S13 Figure.** A panel of forest plots on the within-trial ratio of means (RoM) in the change from baseline of total *Lactobacillus* for chemomechanical methods. Use of correlation coefficient equal to 0.6 as a sensitivity analysis. The vertical grey line implies no difference in the compared interventions. RoM above one favours the second intervention in the comparison, and RoM below one favours the first intervention in the comparison. The trial-design (randomised versus non-randomised-controlled trial) is indicated with different line-colours, the risk of bias (high versus some concerns) is indicated with different point shape, and the method for the bacterial count (CFU versus q-PCR) is indicated with different line-type.

**Table E.** Ratio of ratio of means and 95% confidence interval of total *Streptococcus mutans*

| **ID** | **Trial** | **Comparison** | **RoRoM (SE)** | **95% CI** |
| --- | --- | --- | --- | --- |
| 1 | (44) | Chlorhexidine vs Control | 0.08 (12.74) | Not plausible^1^ |
| 1 | (44) | Laser vs Chlorhexidine | 0.25 (28.69) | Not plausible |
| 1 | (44) | Natural Agents vs Chlorhexidine | 0.35 (21.39) | Not plausible |
| 1 | (44) | Laser vs Control | 0.02 (25.71) | Not plausible |
| 1 | (44) | Natural Agents vs Control | 0.03 (17.18) | Not plausible |
| 1 | (44) | Natural Agents vs Laser | 1.39 (30.92) | Not plausible |
| 2 | (45) | PDT vs Control | 0.66 (0.08) | *0.57 – 0.78*^2^ |
| 3 | (46) | Chlorhexidine vs PDT | 0.84 (0.05) | *0.76 – 0.93* |
| 4 | (51) | Chlorhexidine vs Control | 0.89 (0.05) | *0.81 – 0.98* |
| 5 | (32) | Papain Gel vs Control | 0.60 (0.13) | *0.47 – 0.78* |
| 6 | (54) | SHAA vs Control | 0.62 (0.82) | 0.12 – 3.05 |
| 6 | (55) | Papain Gel vs SHAA | 2.26 (0.82) | 0.46 – 11.17 |
| 6 | (55) | Papain Gel vs Control | 1.39 (0.45) | 0.57 – 3.36 |
| 7 | (56) | Chlorhexidine versus Ozone | 1.00 (0.01) | 0.98 – 1.02 |

CI: confidence interval; PDT: Photodynamic Therapy; RoRoM: ratio of ratio of means in change from baseline; SE: standard error; SHAA: Sodium Hypochlorite and Amino Acids.

^1^A considerable standard error results in implausibly wide 95% confidence intervals that are not interpretable.

^2^Results in italic indicate strong evidence in favour of the first (RoRoM < 1) or second intervention in the comparison (RoRoM > 1).


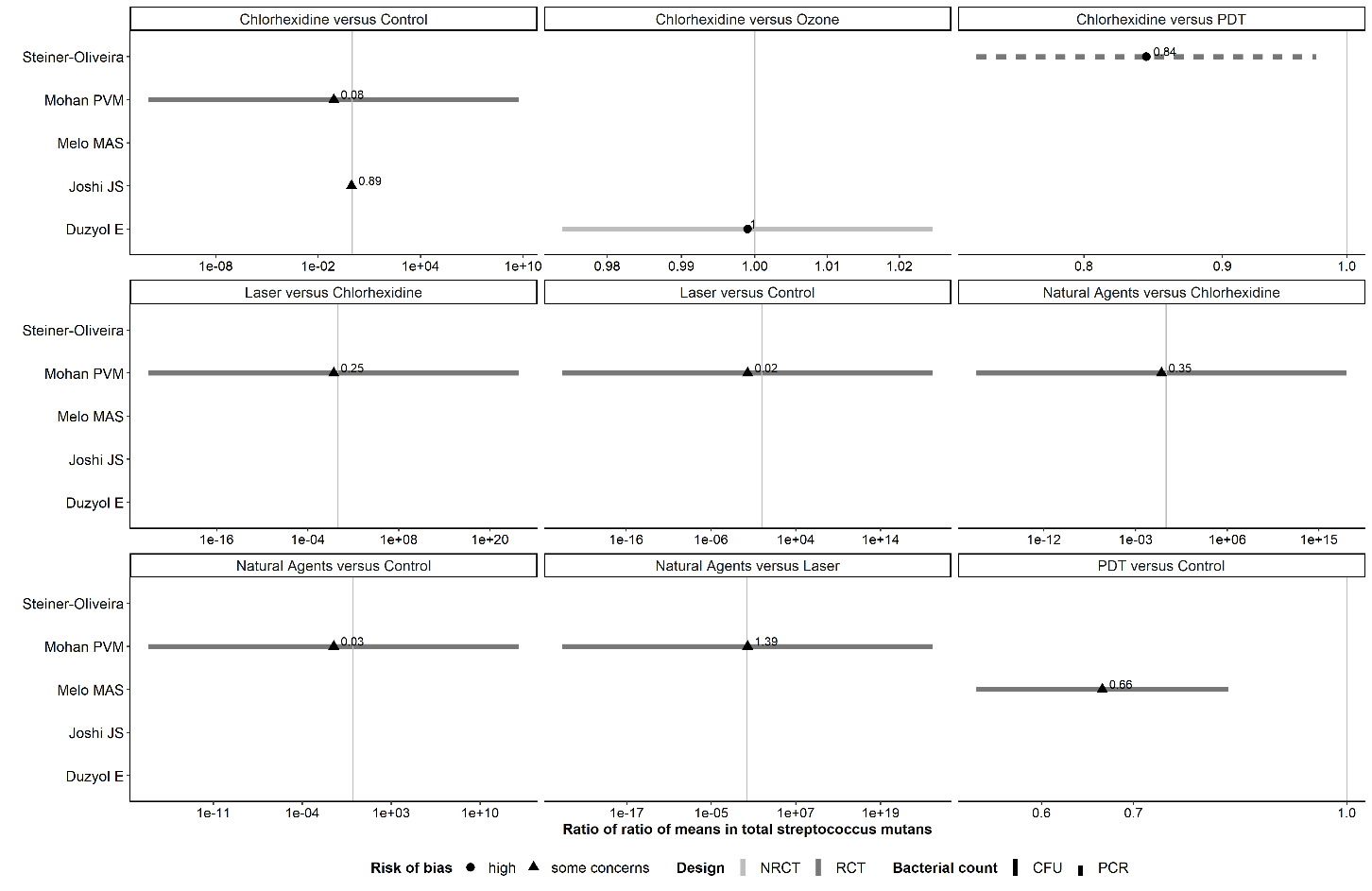


**S14 Figure.** A panel of forest plots on the within-trial ratio of means (RoM) in the change from baseline of total *Streptococcus mutans* for antiseptics. Use of correlation coefficient equal to 0.6 as a sensitivity analysis. The vertical grey line implies no difference in the compared interventions. RoM above one favours the second intervention in the comparison, and RoM below one favours the first intervention in the comparison. The trial-design (randomised versus non-randomised-controlled trial) is indicated with different line-colours, the risk of bias (high versus some concerns) is indicated with different point shape, and the method for the bacterial count (CFU versus q-PCR) is indicated with different line-type.

**
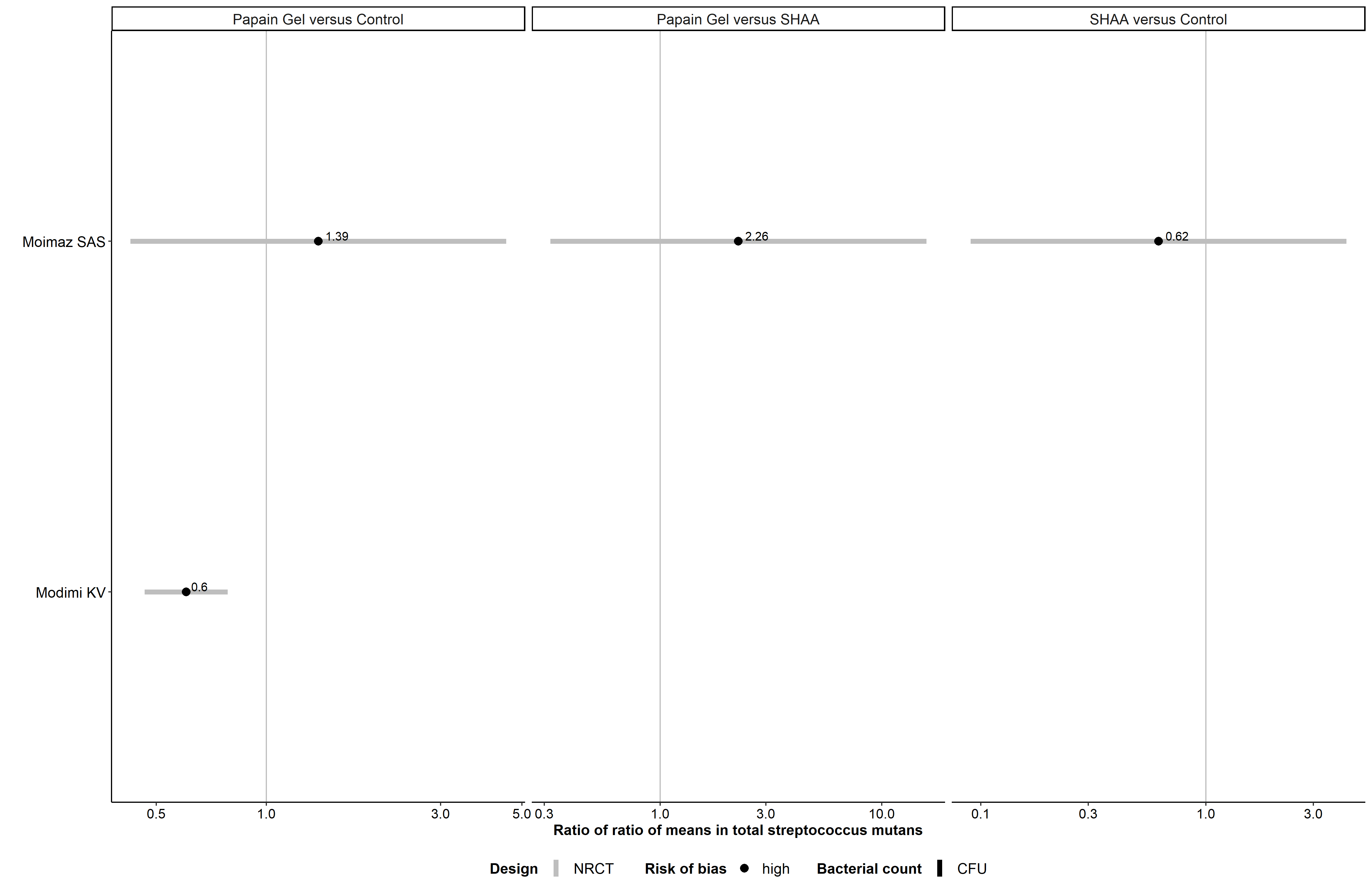
**

**S15 Figure.** A panel of forest plots on the within-trial ratio of means (RoM) in the change from baseline of total *Streptococcus mutans* for chemomechanical methods. Use of correlation coefficient equal to 0.6 as a sensitivity analysis. The vertical grey line implies no difference in the compared interventions. RoM above one favours the second intervention in the comparison, and RoM below one favours the first intervention in the comparison. The trial-design (randomised versus non-randomised-controlled trial) is indicated with different line-colours, the risk of bias (high versus some concerns) is indicated with different point shape, and the method for the bacterial count (CFU versus q-PCR) is indicated with different line-type.

***Secondary outcomes: Pain (Mean Pulse Rate/MIN) and Pain***


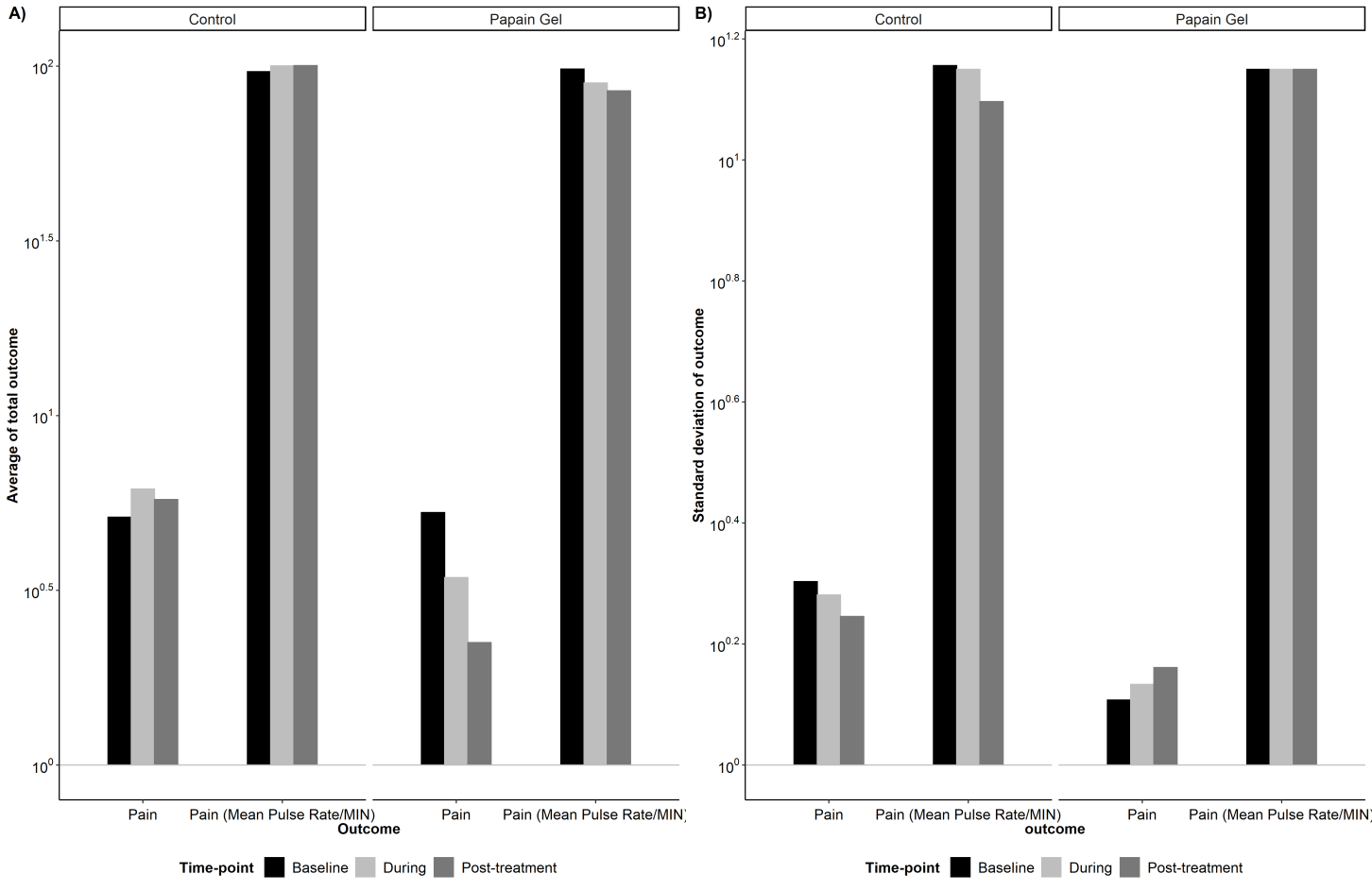
 **S16 Figure.** A panel of bar plots on A) the average of the studied outcome (i.e., Pain 'Mean Pulse Rate/MIN' or Pain) and B) the standard deviation of the studied outcome of each intervention arm of the trial 'Goyal PA' (43) at baseline, during and post-intervention. Goyal PA is an RCT which is associated with high risk of bias and used CFU to count bacterial.


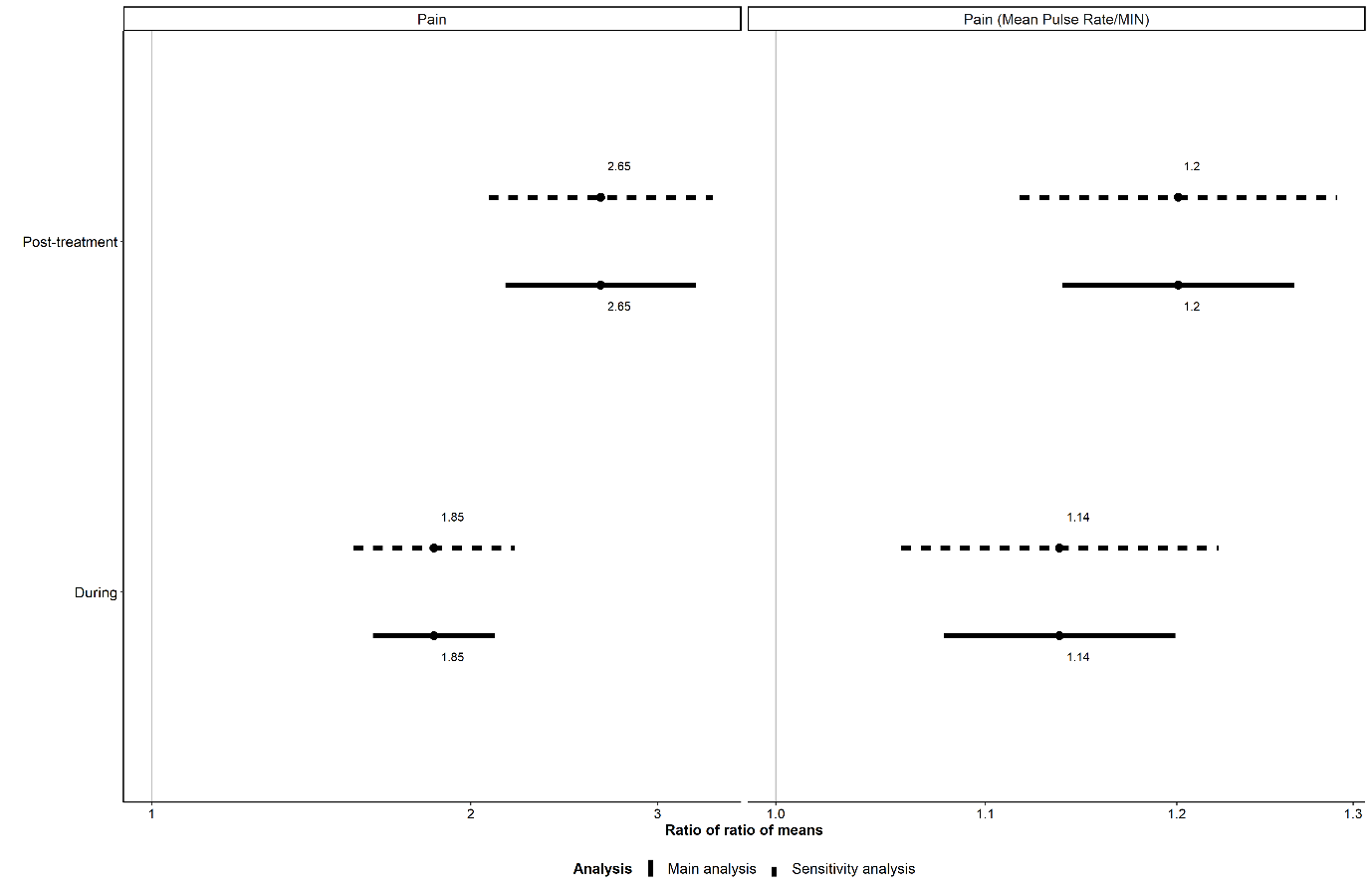


**S17 Figure.** A panel of forest plots on the within-trial ratio of ratio of means (RoRoM) between papain gel and control in the studied outcome (i.e., Pain 'Mean Pulse Rate/MIN' or Pain). Both the main analysis and sensitivity analysis have been performed using correlation coefficient equal to 0.8 and 0.6, respectively. The vertical grey line implies no difference in the compared interventions. RoRoM above one favors the second intervention in the comparison, and RoRoM below one favors the first intervention in the comparison. Results refer to the trial 'Goyal PA' (43), a RCT which is associated with high risk of bias and used CFU to count bacterial.

**Table F.** Ratio of ratio of means and 95% confidence interval of pain outcomes

| **ID** | **Trial** | **Comparison** | **RoRoM (SE)** | **95% CI** |
| --- | --- | --- | --- | --- |
| *Pain (Mean Pulse Rate/MIN)* | | | | |
| 1 | (43) | Control vs Papain Gel | 1.14^1^ (0.03) | *1.08 – 1.20*^3^ |
| 1 | (43) | Control vs Papain Gel | 1.20^2^ (0.03) | *1.14 – 1.27* |
| *Pain* | | | | |
| 1 | (43) | Control vs Papain Gel | 1.85^1^ (0.07) | *1.62 – 2.11* |
| 1 | (43) | Control vs Papain Gel | 2.65^2^ (0.11) | *2.16 – 3.26* |

CI: confidence interval; RoRoM: ratio of ratio of means; SE: standard error.

^1^Ratio of during treatment to baseline in the first versus the second intervention.

^2^Ratio of post-treatment to baseline in the first versus the second intervention

^3^Results in italic indicate strong evidence in favor of the first (RoRoM < 1) or second intervention in the comparison (RoRoM > 1).

**Table G.** Summary of finding (SoF) table showing the certainty of the evidence using the GRADE approach for antiseptic agents in total bacteria.

| **Certainty assessment** | | | | | | | **№ of patients** | | **Effect** | | **Certainty** |
| --- | --- | --- | --- | --- | --- | --- | --- | --- | --- | --- | --- |
| **№ of studies** | **Study design** | **Risk of bias** | **Inconsistency** | **Indirectness** | **Imprecision** | **Other considerations** | **antibacterial agents** | **comparison** | **Relative (95% CI)** | **Absolute (95% CI)** |  |
| **Total bacteria comparing chlorhexidine versus control** | | | | | | | | | | | |
| 1  Mohan PVM | randomised trials | serious^a^ | not serious | very serious^b^ | serious^c^ | none | 20 | 20 | - | RoM **0.23 higher** (0.03 higher to 1.83 higher) | ⨁◯◯◯ Very low |
| 1  Patri G | observational studies | serious^d^ | not serious | serious^e^ | serious^c^ | none | 10 | 10 | - | RoM **0.05 higher** (0.02 higher to 0.11 higher) | ⨁◯◯◯ Very low |
| 1  Joshi JS | observational studies | serious^f^ | not serious | very serious^g^ | serious^c^ | none | 30 | 30 | - | RoM **1.09 higher** (0.99 higher to 1.2 higher) | ⨁◯◯◯ Very low |
| **Total bacteria comparing chlorhexidine versus ozone** | | | | | | | | | | | |
| 1  Hauser-Gerspach I | observational studies | very serious^h^ | not serious | very serious^i^ | serious^c^ | none | 20 | 20 | - | RoM **0.98 higher** (0.97 higher to 1 higher) | ⨁◯◯◯ Very low |
| 1  Krunic J | randomised trials | very serious^j^ | not serious | very serious^k^ | serious^c^ | none | 24 | 24 | - | RoM **2.05 higher** (0.87 higher to 4.84 higher) | ⨁◯◯◯ Very low |
| **Total bacteria comparing chlorhexidine versus PDT** | | | | | | | | | | | |
| 1  Steiner-Oliveira | randomised trials | very serious^l^ | not serious | very serious^m^ | serious^c^ | none | 10 | 12 | - | RoM **1.14 higher** (1.08 higher to 1.21 higher) | ⨁◯◯◯ Very low |
| **Total bacteria comparing laser versus chlorhexidine** | | | | | | | | | | | |
| 1  Mohan PVM | randomised trials | serious^a^ | not serious | very serious^b^ | serious^c^ | none | 20 | 20 | - | RoM **0.2 higher** (0.001 higher to 78.3 higher) | ⨁◯◯◯ Very low |
| **Total bacteria comparing laser versus control** | | | | | | | | | | | |
| 1  Mohan PVM | randomised trials | serious^a^ | not serious | very serious^b^ | serious^c^ | none | 20 | 20 | - | RoM **0.04 higher** (0.0002 higher to 12.15 higher) | ⨁◯◯◯ Very low |
| **Total bacteria comparing natural agents versus chlorhexidine** | | | | | | | | | | | |
| 1  Patri G | observational studies | very serious^d^ | not serious | serious^e^ | serious^c^ | none | 10 | 10 | - | RoM **4.77 higher** (1.95 higher to 11.66 higher) | ⨁◯◯◯ Very low |
| 1  Mohan PVM | randomised trials | very serious^a^ | not serious | very serious^b^ | serious^c^ | none | 20 | 20 | - | RoM **0.61 higher** (0.04 higher to 10.35 higher) | ⨁◯◯◯ Very low |
| **Total bacteria comparing natural agents versus control** | | | | | | | | | | | |
| 1  Patri G | observational studies | very serious^d^ | not serious | serious^e^ | serious^c^ | none | 10 | 10 | - | RoM **0.22 higher** (0.2 higher to 0.25 higher) | ⨁◯◯◯ Very low |
| 1  Mohan PVM | randomised trials | serious^a^ | not serious | very serious^b^ | serious^c^ | none | 20 | 20 | - | RoM **0.14 higher** (0.02 higher to 0.93 higher) | ⨁◯◯◯ Very low |
| **Total bacteria comparing natural agents versus laser** | | | | | | | | | | | |
| 1  Mohan PVM | randomised trials | serious^a^ | not serious | very serious^b^ | serious^c^ | none | 20 | 20 | - | RoM **3.09 higher** (0 to 0 ) | ⨁◯◯◯ Very low |
| **Total bacteria comparing PDT versus control** | | | | | | | | | | | |
| 1  Melo MAS | randomised trials | Serious^n^ | not serious | very serious^o^ | serious^c^ | none | 45 | 45 | - | RoM **0.89 higher** (0.8 higher to 1 higher) | ⨁◯◯◯ Very low |

**CI:** confidence interval

#### Explanations

1. Risk of bias assessed with Rob 2.0 presented some concerns, mainly regarding the careers delivering the

intervention wasn't blind.

b. Applied only to class I, with rubber dam isolation and primary teeth.

c. There is imprecision once total sample summing treatment and control does not fit the optimal information size (OIS) of 400 patients.

d. Serious risk of bias regarding no information about outcome assessor and presence of confounding’s.

e. Not applied to relative isolation but applied to class I or II.

f. Presence of confounding’s domains

g. Not applied to permanent dentition and relative isolation.

h. Serious risk of bias mainly regarding the absence information about the outcome assessor.

i. Applied only to class I in deciduous teeth.

j. High risk of bias manly because there is a lack of information regarding randomization process.

k. Applied to only permanent teeth and with rubber dam isolation.

l. High risk of bias mainly because the allocation sequence was probably not concealed until interventions.

m. Applied to deciduous teeth with rubber dam isolation.

n. No Information about selecting reported outcomes, assessed with RoB 2.0.

o. Applied only to class I in permanent teeth, with rubber dam isolation.

**Table H.** Summary of finding (SoF) table showing the certainty of the evidence using the GRADE approach for antiseptic agents in *Lactobacillus.*

| **Certainty assessment** | | | | | | | **№ of patients** | | **Effect** | | **Certainty** |
| --- | --- | --- | --- | --- | --- | --- | --- | --- | --- | --- | --- |
| **№ of studies** | **Study design** | **Risk of bias** | **Inconsistency** | **Indirectness** | **Imprecision** | **Other considerations** | **antibacterial agents** | **comparison** | **Relative (95% CI)** | **Absolute (95% CI)** |  |
| ***Lactobacillus* comparing chlorhexidine versus control** | | | | | | | | | | | |
| 1  Mohan PVM | randomised trials | serious^a^ | not serious | very serious^b^ | serious^c^ | none | 20 | 20 | - | RoM **0.07 higher** (0 to 0 ) | ⨁◯◯◯ Very low |
| 1  Joshi JS | observational studies | serious^d^ | not serious | very serious^e^ | serious^c^ | none | 30 | 30 | - | RoM **0.98 higher** (0.91 higher to 1.05 higher) | ⨁◯◯◯ Very low |
| ***Lactobacillus* comparing chlorhexidine versus ozone** | | | | | | | | | | | |
| 1  Krunic J | randomised trials | very serious^f^ | not serious | very serious^g^ | serious^c^ | none | 24 | 24 | - | RoM **0.49 higher** (0.17 higher to 1.37 higher) | ⨁◯◯◯ Very low |
| 1  Duzyol E | observational studies | very serious^d^ | not serious | very serious^g^ | serious^c^ | none | 20 | 20 | - | RoM **0.95 higher** (0.93 higher to 0.97 higher) | ⨁◯◯◯ Very low |
| ***Lactobacillus* comparing laser versus chlorhexidine** | | | | | | | | | | | |
| 1  Mohan PVM | randomised trials | serious^a^ | not serious | very serious^b^ | serious^c^ | none | 20 | 20 | - | RoM **0.34 higher** (0 to 0 ) | ⨁◯◯◯ Very low |
| ***Lactobacillus* comparing laser versus control** | | | | | | | | | | | |
| 1  Mohan PVM | randomised trials | serious^a^ | not serious | very serious^b^ | serious^c^ | none | 20 | 20 | - | RoM **0.02 higher** (0 to 0 ) | ⨁◯◯◯ Very low |
| ***Lactobacillus* comparing natural agents versus chlorhexidine** | | | | | | | | | | | |
| 1  Mohan PVM | randomised trials | serious^a^ | not serious | very serious^b^ | serious^c^ | none | 20 | 20 | - | RoM **0.57 higher** (0 to 0 ) | ⨁◯◯◯ Very low |
| ***Lactobacillus* comparing natural agents versus control** | | | | | | | | | | | |
| 1  Mohan PVM | randomised trials | serious^a^ | not serious | very serious^b^ | serious^c^ | none | 20 | 20 | - | RoM **0.04 higher** (0 to 0 ) | ⨁◯◯◯ Very low |
| ***Lactobacillus* comparing natural agents versus laser** | | | | | | | | | | | |
| 1  Mohan PVM | randomised trials | serious^a^ | not serious | very serious^b^ | serious^c^ | none | 20 | 20 | - | RoM **1.68 higher** (0 to 0 ) | ⨁◯◯◯ Very low |
| ***Lactobacillus* comparing PDT versus control** | | | | | | | | | | | |
| 1  Melo MAS | randomised trials | serious^h^ | not serious | very serious^i^ | serious^c^ | none | 45 | 45 | - | RoM **0.83 higher** (0.68 higher to 1 higher) | ⨁◯◯◯ Very low |

**CI:** confidence interval

**Explanations**

1. Risk of bias assessed with Rob 2.0 presented some concerns, mainly regarding the careers delivering the intervention wasn't blind.
2. Applied only to class I, with rubber dam isolation and primary teeth.
3. There is imprecision once total sample summing treatment and control does not fit the optimal information size (OIS) of 400 patients.
4. Presence of confounding’s domains.
5. Not applied to permanent dentition and relative isolation.
6. High risk of bias manly because there is a lack of information regarding randomization process.
7. Applied to only permanent teeth and with rubber dam isolation.
8. No information about selecting reported outcomes, assessed with RoB 2.0.
9. Applied only to class I in permanent teeth, with rubber dam isolation.

**Table I.** Summary of finding (SoF) table showing the certainty of the evidence using the GRADE approach for antiseptic agents in *Streptococcus mutans.*

| **Certainty assessment** | | | | | | | **№ of patients** | | **Effect** | | **Certainty** |
| --- | --- | --- | --- | --- | --- | --- | --- | --- | --- | --- | --- |
| **№ of studies** | **Study design** | **Risk of bias** | **Inconsistency** | **Indirectness** | **Imprecision** | **Other considerations** | **antibacterial agents** | **comparison** | **Relative (95% CI)** | **Absolute (95% CI)** |  |
| ***Streptococcus mutans* comparing chlorhexidine versus control** | | | | | | | | | | | |
| 1  Mohan PVM | randomised trials | serious^a^ | not serious | very serious^b^ | serious^c^ | none | 20 | 20 | - | RoM **0.08 higher** (0 to 0 ) | ⨁◯◯◯ Very low |
| 1  Joshi JS | observational studies | serious^d^ | not serious | very serious^e^ | serious^c^ | none | 30 | 30 | - | RoM **0.89 higher** (0.81 higher to 0.98 higher) | ⨁◯◯◯ Very low |
| ***Streptococcus mutans* comparing chlorhexidine versus PDT** | | | | | | | | | | | |
| 1  Steiner-Oliveira | randomised trials | very serious^f^ | not serious | very serious^g^ | serious^c^ | none | 10 | 12 | - | RoM **0.84 higher** (0.76 higher to 0.93 higher) | ⨁◯◯◯ Very low |
| ***Streptococcus mutans* comparing laser versus chlorhexidine** | | | | | | | | | | | |
| 1  Mohan PVM | randomised trials | serious^a^ | not serious | very serious^b^ | serious^c^ | none | 20 | 20 | - | RoM **0.25 higher** (0 to 0 ) | ⨁◯◯◯ Very low |
| ***Streptococcus mutans* comparing laser versus control** | | | | | | | | | | | |
| 1  Mohan PVM | randomised trials | serious^a^ | not serious | very serious^b^ | serious^c^ | none | 20 | 20 | - | RoM **0.02 higher** (0 to 0 ) | ⨁◯◯◯ Very low |
| ***Streptococcus mutans* comparing natural agents versus chlorhexidine** | | | | | | | | | | | |
| 1  Mohan PVM | randomised trials | serious^a^ | not serious | very serious^b^ | serious^c^ | none | 20 | 20 | - | RoM **0.35 higher** (0 to 0 ) | ⨁◯◯◯ Very low |
| ***Streptococcus mutans* comparing natural agents versus control** | | | | | | | | | | | |
| 1  Mohan PVM | randomised trials | serious^a^ | not serious | very serious^b^ | serious^c^ | none | 20 | 20 | - | RoM **0.03 higher** (0 to 0 ) | ⨁◯◯◯ Very low |
| ***Streptococcus mutans* comparing natural agents versus laser** | | | | | | | | | | | |
| 1  Mohan PVM | randomised trials | serious^a^ | not serious | very serious^b^ | serious^c^ | none | 20 | 20 | - | RoM **1.39 higher** (0 to 0 ) | ⨁◯◯◯ Very low |
| ***Streptococcus mutans* comparing PDT versus control** | | | | | | | | | | | |
| 1  Melo MAS | randomised trials | Serious^h^ | not serious | very serious^i^ | serious^c^ | none | 45 | 45 | - | RoM **0.66 higher** (0.57 higher to 0.78 higher) | ⨁◯◯◯ Very low |
| ***Streptococcus mutans* comparing chlorhexidine versus ozone** | | | | | | | | | | | |
| 1  Duzyol E | observational studies | very serious^d^ | not serious | very serious^j^ | serious^c^ | none | 20 | 20 | - | RoM **1 higher** (0.98 higher to 1.02 higher) | ⨁◯◯◯ Very low |

**CI:** confidence interval

#### Explanations

1. Risk of bias assessed with Rob 2.0 presented some concerns, mainly regarding the careers delivering the intervention wasn't blind.
2. Applied only to class I, with rubber dam isolation and primary teeth.
3. There is imprecision once total sample summing treatment and control does not fit the optimal information size (OIS) of 400 patients.
4. Presence of confounding’s domains.
5. Not applied to permanent dentition and relative isolation.
6. High risk of bias mainly because the allocation sequence was probably not concealed until interventions.
7. Applied to deciduous teeth with rubber dam isolation.
8. No information about selecting reported outcomes, assessed with RoB 2.0.
9. Applied only to class I in permanent teeth, with rubber dam isolation.
10. Applied to permanent teeth, with rubber dam isolation.

**Table J.** Summary of finding (SoF) table showing the certainty of the evidence using the GRADE approach for chemomechanical methods in total bacteria.

| **Certainty assessment** | | | | | | | **№ of patients** | | **Effect** | | **Certainty** |
| --- | --- | --- | --- | --- | --- | --- | --- | --- | --- | --- | --- |
| **№ of studies** | **Study design** | **Risk of bias** | **Inconsistency** | **Indirectness** | **Imprecision** | **Other considerations** | **antibacterial agents** | **comparison** | **Relative (95% CI)** | **Absolute (95% CI)** |  |
| **Total bacteria comparing papain gel versus control** | | | | | | | | | | | |
| 1  Goyal PA | randomised trials | very serious^a^ | not serious | very serious^b^ | serious^c^ | publication bias strongly suspected^o^ | 25 | 25 | - | RoM **0.98 higher** (0.81 higher to 1.2 higher) | ⨁◯◯◯ Very low |
| 1  Ashwati K | observational studies | very serious^d^ | not serious | very serious^b^ | serious^c^ | publication bias strongly suspected^e^ | 25 | 25 | - | RoM **1.01 higher** (0.95 higher to 1.07 higher) | ⨁◯◯◯ Very low |
| **Total bacteria comparing SHAA versus control** | | | | | | | | | | | |
| 1  Ali AH | randomised trials | serious^f^ | not serious | serious^g^ | serious^c^ | none | 55 | 46 | - | RoM **0.13 higher** (0.06 higher to 0.26 higher) | ⨁◯◯◯ Very low |
| **Total bacteria comparing SHAA versus papain gel** | | | | | | | | | | | |
| 1  Patri G | observational studies | very serious^h^ | not serious | very serious^i^ | serious^c^ | none | 15 | 15 | - | RoM **1.03 higher** (0.88 higher to 1.21 higher) | ⨁◯◯◯ Very low |
| 1  Asal MA | randomised trials | very serious^d^ | not serious | very serious^i^ | serious^c^ | none | 30 | 30 | - | RoM **0.71 higher** (0.62 higher to 0.82 higher) | ⨁◯◯◯ Very low |

**CI:** confidence interval

#### Explanations

1. Risk of bias assessed with RoB 2.0. It's not clear who is the outcome assessor.
2. Applied to deciduous teeth with rubber dam isolation.
3. There is imprecision once total sample summing treatment and control does not fit the optimal information size (OIS) of 400 patients.
4. Serious risk of bias mainly regarding the absence information about the outcome assessor.
5. Material provided
6. No Information about selecting reported outcomes, assessed with RoB 2.0.
7. Applied only to permanent teeth.
8. Serious risk of bias regarding no information about outcome assessor and presence of confounding’s.
9. Applied only to class I, with rubber dam isolation and primary teeth.

**Table K.** Summary of finding (SoF) table showing the certainty of the evidence using the GRADE approach for chemo-mechanical methods in *Lactobacillus.*

| **Certainty assessment** | | | | | | | **№ of patients** | | **Effect** | | **Certainty** |
| --- | --- | --- | --- | --- | --- | --- | --- | --- | --- | --- | --- |
| **№ of studies** | **Study design** | **Risk of bias** | **Inconsistency** | **Indirectness** | **Imprecision** | **Other considerations** | **antibacterial agents** | **comparison** | **Relative (95% CI)** | **Absolute (95% CI)** |  |
| ***Lactobacillus* comparing papain gel versus control** | | | | | | | | | | | |
| 1  Moimaz MAS | observational studies | very serious^a^ | not serious | very serious^b^ | serious^c^ | none | 16 | 32 | - | RoM **0.62 higher** (0 to 0 ) | ⨁◯◯◯ Very low |
| 1  Modimi KV | observational studies | very serious^a^ | not serious | very serious^d^ | serious^c^ | none | 30 | 30 | - | RoM **0.9 higher** (0.75 higher to 1.08 higher) | ⨁◯◯◯ Very low |
| 1  Goyal PA | randomised trials | very serious^a^ | not serious | very serious^e^ | serious^c^ | publication bias strongly suspected^f^ | 25 | 25 | - | RoM **1.36 higher** (0.98 higher to 1.88 higher) | ⨁◯◯◯ Very low |
| ***Lactobacillus* comparing papain gel versus SHAA** | | | | | | | | | | | |
| 1  Moimaz SAS | observational studies | very serious^a^ | not serious | very serious^b^ | serious^c^ | none | 16 | 32 | - | RoM **1.08 higher** (0 to 0 ) | ⨁◯◯◯ Very low |
| ***Lactobacillus* comparing SHAA versus control** | | | | | | | | | | | |
| 1  Moimaz SAS | observational studies | very serious^a^ | not serious | very serious^b^ | serious^c^ | none | 16 | 32 | - | RoM **0.58 higher** (0.04 higher to 8.57 higher) | ⨁◯◯◯ Very low |
| ***Lactobacillus* comparing SHAA versus papain gel** | | | | | | | | | | | |
| 1  Reddy MVC | observational studies | very serious^a^ | not serious | very serious^g^ | serious^c^ | none | 15 | 15 | - | RoM **1.03 higher** (0.8 higher to 1.33 higher) | ⨁◯◯◯ Very low |

**CI:** confidence interval

#### Explanations

1. Absence information about the outcome assessor.
2. Applied only to class I in primary teeth, with relative isolation.
3. There is imprecision once total sample summing treatment and control does not fit the optimal information size (OIS) of 400 patients.
4. Applied to both dentitions, but only with rubber dam isolation.
5. Applied to deciduous teeth with rubber dam isolation.
6. Company provided the material for research
7. Applied only to class I, with rubber dam isolation and primary teeth.

**Table L.** Summary of finding (SoF) table showing the certainty of the evidence using the GRADE approach for chemomechanical methods in *Streptococcus mutans.*

| **Certainty assessment** | | | | | | | **№ of patients** | | **Effect** | | **Certainty** |
| --- | --- | --- | --- | --- | --- | --- | --- | --- | --- | --- | --- |
| **№ of studies** | **Study design** | **Risk of bias** | **Inconsistency** | **Indirectness** | **Imprecision** | **Other considerations** | **antibacterial agents** | **comparison** | **Relative (95% CI)** | **Absolute (95% CI)** |  |
| ***Streptococcus mutans* comparing papain gel versus control** | | | | | | | | | | | |
| 1  Moimaz SAS | observational studies | very serious^a^ | not serious | very serious^b^ | serious^c^ | none | 16 | 32 | - | RoM **1.39 higher** (0.57 higher to 3.36 higher) | ⨁◯◯◯ Very low |
| 1  Modimi KV | observational studies | very serious^a^ | not serious | very serious^d^ | serious^c^ | none | 30 | 30 | - | RoM **0.6 higher** (0.47 higher to 0.78 higher) | ⨁◯◯◯ Very low |
| ***Streptococcus mutans* comparing papain gel versus SHAA** | | | | | | | | | | | |
| 1  Moimaz SAS | observational studies | very serious^a^ | not serious | very serious^b^ | serious^c^ | none | 16 | 16 | - | RoM **2.26 higher** (0.46 higher to 11.17 higher) | ⨁◯◯◯ Very low |
| ***Streptococcus mutans* comparing SHAA versus control** | | | | | | | | | | | |
| 1  Moimaz SAS | observational studies | very serious^a^ | not serious | very serious^b^ | serious^c^ | none | 16 | 32 | - | RoM **0.62 higher** (0.57 higher to 3.36 higher) | ⨁◯◯◯ Very low |

**CI:** confidence interval

#### Explanations

1. Absent information about the outcome assessor.
2. Applied only to class I in primary teeth, with relative isolation.
3. There is imprecision once total sample summing treatment and control does not fit the optimal information size (OIS) of 400 patients.
4. Applied to both dentitions, but only with rubber dam isolation.

**Table M.** Summary of finding (SoF) table showing the certainty of the evidence using the GRADE approach for chemomechanical agents in *pain.*

| **Certainty assessment** | | | | | | | **№ of patients** | | **Effect** | **Certainty** |
| --- | --- | --- | --- | --- | --- | --- | --- | --- | --- | --- |
| **№ of studies** | **Study design** | **Risk of bias** | **Inconsistency** | **Indirectness** | **Imprecision** | **Other considerations** | **antibacterial agents** | **comparison** | **Absolute (95% CI)** |  |
| **Pain (Mean Pulse Rate/ Min) comparing papain gel versus control during treatment** | | | | | | | | | | |
| 1  Goyal PA | randomized trials | very serious ^a^ | not serious | serious ^b^ | serious ^c^ | publication bias strongly suspected ^d^ | 25 | 25 | RoRoM **1.14 higher** (1.08 higher to 1.2 higher) | ⨁◯◯◯ VERY LOW |
| **Pain (Mean Pulse Rate/ Min) comparing papain gel versus control post treatment** | | | | | | | | | | |
| 1  Goyal PA | randomized trials | very serious ^a^ | not serious | serious ^b^ | serious ^c^ | publication bias strongly suspected ^d^ | 25 | 25 | RoRoM **1.2 higher** (1.14 higher to 1.27 higher) | ⨁◯◯◯ VERY LOW |
| **Pain comparing papain gel versus control during treatment** | | | | | | | | | | |
| 1  Goyal PA | randomized trials | very serious ^a^ | not serious | serious ^b^ | serious ^c^ | publication bias strongly suspected ^d^ | 25 | 25 | RoRoM **1.85 higher** (1.62 higher to 2.11 higher) | ⨁◯◯◯ VERY LOW |
| **Pain comparing papain gel versus control post treatment** | | | | | | | | | | |
| 1  Goyal PA | randomized trials | very serious ^a^ | not serious | serious ^b^ | serious ^c^ | publication bias strongly suspected ^d^ | 25 | 25 | RoRoM **2.65 higher** (2.16 higher to 3.26 higher) | ⨁◯◯◯ VERY LOW |

**CI:** Confidence interval

#### Explanations

1. Absence information about the outcome assessor.

b. Applied only for primary teeth.

c. There is imprecision once total sample summing treatment and control does not fit the optimal information size (OIS) of 400 patients.

d. Company provided the material for research.

**Table N.** Prisma Checklist.

| **Section and Topic** | **Item #** | **Checklist item** | **Location where item is reported (# page)** |
| --- | --- | --- | --- |
| **TITLE** | | |  |
| Title | 1 | Identify the report as a systematic review. | #1 |
| **ABSTRACT** | | |  |
| Abstract | 2 | See the PRISMA 2020 for Abstracts checklist. | #2 |
| **INTRODUCTION** | | |  |
| Rationale | 3 | Describe the rationale for the review in the context of existing knowledge. | #3-4 |
| Objectives | 4 | Provide an explicit statement of the objective(s) or question(s) the review addresses. | #4 |
| **METHODS** | | |  |
| Eligibility criteria | 5 | Specify the inclusion and exclusion criteria for the review and how studies were grouped for the syntheses. | #4 |
| Information sources | 6 | Specify all databases, registers, websites, organisations, reference lists and other sources searched or consulted to identify studies. Specify the date when each source was last searched or consulted. | #6 |
| Search strategy | 7 | Present the full search strategies for all databases, registers and websites, including any filters and limits used. | Appendix Table 1, #1 |
| Selection process | 8 | Specify the methods used to decide whether a study met the inclusion criteria of the review, including how many reviewers screened each record and each report retrieved, whether they worked independently, and if applicable, details of automation tools used in the process. | #6 |
| Data collection process | 9 | Specify the methods used to collect data from reports, including how many reviewers collected data from each report, whether they worked independently, any processes for obtaining or confirming data from study investigators, and if applicable, details of automation tools used in the process. | #7 |
| Data items | 10a | List and define all outcomes for which data were sought. Specify whether all results that were compatible with each outcome domain in each study were sought (e.g. for all measures, time points, analyses), and if not, the methods used to decide which results to collect. | #7-8 |
|  | 10b | List and define all other variables for which data were sought (e.g. participant and intervention characteristics, funding sources). Describe any assumptions made about any missing or unclear information. | #7 |
| Study risk of bias assessment | 11 | Specify the methods used to assess risk of bias in the included studies, including details of the tool(s) used, how many reviewers assessed each study and whether they worked independently, and if applicable, details of automation tools used in the process. | #7 |
| Effect measures | 12 | Specify for each outcome the effect measure(s) (e.g. risk ratio, mean difference) used in the synthesis or presentation of results. | #7, appendix #12 |
| Synthesis methods | 13a | Describe the processes used to decide which studies were eligible for each synthesis (e.g. tabulating the study intervention characteristics and comparing against the planned groups for each synthesis (item #5)). | #4 |
|  | 13b | Describe any methods required to prepare the data for presentation or synthesis, such as handling of missing summary statistics, or data conversions. | appendix #14 |
|  | 13c | Describe any methods used to tabulate or visually display results of individual studies and syntheses. | #8 |
|  | 13d | Describe any methods used to synthesize results and provide a rationale for the choice(s). If meta-analysis was performed, describe the model(s), method(s) to identify the presence and extent of statistical heterogeneity, and software package(s) used. | #8-9 |
|  | 13e | Describe any methods used to explore possible causes of heterogeneity among study results (e.g. subgroup analysis, meta-regression). | Appendix #14 |
|  | 13f | Describe any sensitivity analyses conducted to assess robustness of the synthesized results. | Appendix #14 |
| Reporting bias assessment | 14 | Describe any methods used to assess risk of bias due to missing results in a synthesis (arising from reporting biases). | #7 |
| Certainty assessment | 15 | Describe any methods used to assess certainty (or confidence) in the body of evidence for an outcome. | #9 |
| **RESULTS** | | |  |
| Study selection | 16a | Describe the results of the search and selection process, from the number of records identified in the search to the number of studies included in the review, ideally using a flow diagram. | #9 |
|  | 16b | Cite studies that might appear to meet the inclusion criteria, but which were excluded, and explain why they were excluded. | Appendix Table B, #11 |
| Study characteristics | 17 | Cite each included study and present its characteristics. | #10 |
| Risk of bias in studies | 18 | Present assessments of risk of bias for each included study. | #13 |
| Results of individual studies | 19 | For all outcomes, present, for each study: (a) summary statistics for each group (where appropriate) and (b) an effect estimate and its precision (e.g. confidence/credible interval), ideally using structured tables or plots. | #14 |
| Results of syntheses | 20a | For each synthesis, briefly summarise the characteristics and risk of bias among contributing studies. | #9 |
|  | 20b | Present results of all statistical syntheses conducted. If meta-analysis was done, present for each the summary estimate and its precision (e.g. confidence/credible interval) and measures of statistical heterogeneity. If comparing groups, describe the direction of the effect. | #14 |
|  | 20c | Present results of all investigations of possible causes of heterogeneity among study results. | #26 |
|  | 20d | Present results of all sensitivity analyses conducted to assess the robustness of the synthesized results. | Appendix #21 |
| Reporting biases | 21 | Present assessments of risk of bias due to missing results (arising from reporting biases) for each synthesis assessed. | #13 |
| Certainty of evidence | 22 | Present assessments of certainty (or confidence) in the body of evidence for each outcome assessed. | Appendix #40 |
| **DISCUSSION** | | |  |
| Discussion | 23a | Provide a general interpretation of the results in the context of other evidence. | #24 |
|  | 23b | Discuss any limitations of the evidence included in the review. | #27 |
|  | 23c | Discuss any limitations of the review processes used. | #27 |
|  | 23d | Discuss implications of the results for practice, policy, and future research. | #28 |
| **OTHER INFORMATION** | | |  |
| Registration and protocol | 24a | Provide registration information for the review, including register name and registration number, or state that the review was not registered. | #4 |
|  | 24b | Indicate where the review protocol can be accessed, or state that a protocol was not prepared. | #4 |
|  | 24c | Describe and explain any amendments to information provided at registration or in the protocol. | #4 |
| Support | 25 | Describe sources of financial or non-financial support for the review, and the role of the funders or sponsors in the review. | #28 |
| Competing interests | 26 | Declare any competing interests of review authors. | #28 |
| Availability of data, code and other materials | 27 | Report which of the following are publicly available and where they can be found: template data collection forms; data extracted from included studies; data used for all analyses; analytic code; any other materials used in the review. | Supplementary File 1 |

**References Supplementary Material**

1. Ersin NK, Uzel A, Aykut A, Candan U, Eronat C. Inhibition of cultivable bacteria by chlorhexidine treatment of dentin lesions treated with the ART technique. Caries Res [Internet]. 2006;40(2 CC-Oral Health):172‐177. Available from: <https://www.cochranelibrary.com/central/doi/10.1002/central/CN-00555470/full>**.**
2. Comparison of two methods for caries removal among 5-9-year-old children. 2018; [accessed 2020 Jan 06]. [http://www.who.int/trialsearch/Trial2.aspx?TrialID=CTRI/2018/04/01 3080](http://www.who.int/trialsearch/Trial2.aspx?TrialID=CTRI/2018/04/01%203080).
3. Costa-Santos L, Silva-Júnior ZS, Sfalcin RA, da Mota ACC, Horliana ACRT Motta LJ, Mesquita-Ferrari RA, Fernandes KPS, Prates RA, Silva DFT, Deana A, Bussadori SK. 2019. The effect of antimicrobial photodynamic therapy on infected dentin in primary teeth A randomized controlled clinical trial protocol. Medicine. 98(15): e15110. Effect of a chlorhexidine-modified filling to treat root decay on cariogenic bacteria in elderly participants: A feasibility study. 2016; [accessed 2020 Jan 06]. https://www.anzctr.org.au/Trial/Registration/TrialReview.aspx?id=37 1494.
4. Effect of Low-intensity Laser and Photodynamic Therapy on Infected Dentin of Permanent Teeth With MIH. 2019; [accessed 2020 Jan 06]. https://clinicaltrials.gov/show/NCT03826810.
5. Evaluation of the Antibacterial Effect of Laser Diode and Zinc Oxide Nano Particles in Cavity Disinfection. 2018; [accessed 2020 Jan 06]. <https://clinicaltrials.gov/show/NCT03478150>.
6. Microbiological Assessment After Chemo-Mechanical Caries Removal Using Papain-based Enzyme Versus Conventional Rotary Tools in Occlusal Carious Lesions. 2018; [accessed 2020 Jan 06]. https://clinicaltrials.gov/show/NCT03408626.
7. The Microbiological Assessment of Deep Carious Lesions After Stepwise Excavation and Diode Laser Cavity Disinfection. 2017; [accessed 2020 Jan 06]. <https://clinicaltrials.gov/show/NCT03298165>.
8. Baysan A, Beighton D. Assessment of the ozone-mediated killing of bacteria in infected dentine associated with non-cavitated occlusal carious lesions. Caries Res [Internet]. 2007;41(5 CC-Complementary Medicine CC-Oral Health):337‐341. Available from: <https://www.cochranelibrary.com/central/doi/10.1002/central/CN-00611535/full>.
9. de Castilho ARF, Duque C, Kreling PF, Pereira JA, de aula AB, Sinhoreti MAC, et al. Doxycycline-containing glass ionomer cement for arresting residual caries: An in vitro study and a pilot trial. J Appl Oral Sci. 2018;26:1–8.
10. Di Paolo N, Bocci V, Gaggiotti E. Ozone therapy. Int J Artif Organs [Internet]. 2004;27(3):168–75. Available from: https://libaccess.mcmaster.ca/login?url=http://ovidsp.ovid.com/ovidweb.cgi?T=JS&CSC=Y&NEWS=N&PAGE=fulltext&D=emed8&AN=38594350 <http://sfx.scholarsportal.info/mcmaster?sid=OVID:embase&id=pmid:15112882&id=doi:&issn=0391-3988&isbn=&volume=27&issue=3&spage=16>.
11. Kochhar GK, Srivastava N, Pandit IK, Gugnani N, Gupta M. An evaluation of different caries removal techniques in primary teeth: a comparitive clinical study. J Clin Pediatr Dent [Internet]. 2011;36(1):5‐9. Available from: <https://www.cochranelibrary.com/central/doi/10.1002/central/CN-00832204/full>.
12. Kuhn E, Reis A, Chibinski AC, Wambier DS. The influence of the lining material on the repair of the infected dentin in young permanent molars after restoration: A randomized clinical trial. J Conserv Dent. 2016;19(6):516–21.
13. Rajakumar S, Mungara J, Joseph E, Philip J, Shilpa Priya MP. Evaluation of three different caries removal techniques in children: a comparative clinical study. J Clin Pediatr Dent [Internet]. 2013;38(1):23–6. Available from: <https://libaccess.mcmaster.ca/login?url=http://ovidsp.ovid.com/ovidweb.cgi?T=JS&CSC=Y&NEWS=N&PAGE=fulltext&D=med10&AN=24579278>.
14. Safwat O, Elkateb M, Dowidar K, Salam HA, El Meligy O. Microbiological Evaluation of Ozone on Dentinal Lesions in Young Permanent Molars using the Stepwise Excavation. J Clin Pediatr Dent [Internet]. 2018;42(1 CC-Oral Health):11‐20. Available from: <https://www.cochranelibrary.com/central/doi/10.1002/central/CN-01454123/full>.
15. Baysan A, Lynch E. Effect of ozone on the oral microbiota and clinical severity of primary root caries. Am J Dent. 2004;17(1):56–60.
16. RS K, Pradeep S. Chemomechanical agents used in caries excavation. Res J Pharm Technol. 2016;9(10):1765.
17. An in Vitro Evaluation of Antimicrobial Photodynamic Therapy on Deciduous Carious Dentin. 2015; [accessed 2020 Jan 06]. <https://clinicaltrials.gov/show/NCT02479958>.
18. Antibacterial Effect of Nano Silver Fluoride vs Chlorhexidine on Occlusal Carious Molars Treated with Partial Caries Removal Technique.
    <https://clinicaltrials.gov/show/NCT03186261>.
19. A clinical trial to study the efficacy of chemo mechanical caries removal. 2013; [accessed 2020 Jan 06]. http://www.who.int/trialsearch/Trial2.aspx?TrialID=CTRI/2013/05/00 3637.
20. Effect of Photodynamic Therapy with Low-level Laser on Infected Dentin in Primary Teeth: a Controlled Clinical Trial. 2013; [accessed 2020 Jan 06]. <https://clinicaltrials.gov/show/NCT02734420>.
21. Influence of Ozone Application in Stepwise Excavation Procedure of Primary Molars with Deep Carious Lesion. 2018; [accessed 2020 Jan 06]. <https://clinicaltrials.gov/show/NCT03737201>.
22. Callaway A, Kostrzewa M, Willershausen B, Schmidt F, Thiede B, Kupper H, Kneist S. 2013. Identification of lactobacilli from deep carious lesions by means of species-specific PCR and MALDI-TOF mass spectrometry. Clin Lab. 59(11-12): 1373-1379.
23. Chibinski ACR, Wambier L, Reis A, Wambier DS. 2016. Clinical, mineral and ultrastructural changes in carious dentin of primary molars after restoration. Int Dent J. 66(3): 150-157.
24. Dommisch H, Peus. K, Kneist S, Krause F, Braun A, Hedderich J, Jepsen S, Eberhard J. 2008. Fluorescence-controlled Er : YAG laser for caries removal in permanent teeth: a randomized clinical trial. Eur J Oral Sci. 116(2): 170-176.
25. Frencken JE, Imazato S, Toi C, Mulder J, Mickenautsch S, Takahashi Y, Ebisu S. 2007. Antibacterial effect of chlorhexidine- containing glass ionomer cement in vivo: a pilot study. Caries Res. 41(2): 102‐107.
26. Kabil NS, Badran AS, Wassel MO. 2017. Effect of the addition of chlorhexidine and miswak extract on the clinical performance and antibacterial properties of conventional glass ionomer: an in vivo study. Int J of Paediatr Dent. 27(5): 380‐387.
27. Krause F, Braun A, Lotz G, Kneist S, Jepsen S, Eberhard J. 2008. Evaluation of selective caries removal in deciduous teeth by a fluorescence feedback-controlled Er:YAG laser in vivo. Clin Oral Investig. 12(3): 209-215.
28. Sadasiva K, Kumar K, Rayar S, Shamini S, Unnikrishnan M, Kandaswamy D. 2019. Evaluation of the efficacy of visual, tactile method, caries detector dye, and laser fluorescence in removal of dental caries and confirmation by culture and polymerase chain reaction: An in vivo study. J Pharma Bioallied Sci. 11(6): S146-S150.
29. Schmidt F, Fiege T, Hustoft HK, Kneist S, Thiede B. 2009. Shotgun mass mapping of Lactobacillus species and subspecies from caries related isolates by MALDI-MS. Proteomics. 9(7): 1994-2003.
30. Valério RA, Borsatto MC, Serra MC, Polizeli SAF, Nemezio MA, Galo R, Aires CP, dos Santos AC, Corona SAM. 2016. Caries removal in deciduous teeth using an Er:YAG laser: a randomized split-mouth clinical trial. Clin Oral Investig. 20(1): 65-73.
31. Wicht MJ, Haak R, Kneist S, Noack MJ. 2005. A triclosan-containing compomer reduces Lactobacillus spp. predominant in advanced carious lesions. Dent Mater. 21(9): 831-836.
32. Wicht MJ, Haak R, Lummert D, Noack MJ. 2003. Treatment of root caries lesions with chlorhexidine-containing varnishes and dentin sealants. Am J Dent. 16 Spec No: 25A‐30A.
33. Wicht MJ, Haak R, Schutt-Gerowitt H, Kneist S, Noack MJ. 2004. Suppression of caries-related microorganisms in dentine lesions after short-term chlorhexidine or antibiotic treatment. Caries Res. 38(5): 436- 441.
34. Longo JPF, Leal SC, Simioni AR, De Fatima Menezes Almeida-Santos M, Tedesco AC, Azevedo RB. Photodynamic therapy disinfection of carious tissue mediated by aluminum-chloride-phthalocyanine entrapped in cationic liposomes: An in vitro and clinical study. Lasers Med Sci [Internet]. 2012;27(3):575–84. Available from: https://libaccess.mcmaster.ca/login?url=http://ovidsp.ovid.com/ovidweb.cgi?T=JS&CSC=Y&NEWS=N&PAGE=fulltext&D=emed13&AN=51552924 <http://sfx.scholarsportal.info/mcmaster?sid=OVID:embase&id=pmid:21809069&id=doi:10.1007%2Fs10103-011-0962-6&issn=0268-8921&isbn>
35. Araújo PV, Correia-Silva J de F, Gomez RS, Massara M de L de A, Cortes ME, Poletto LT de A. Antimicrobial effect of photodynamic therapy in carious lesions in vivo, using culture and real-time PCR methods. Photodiagnosis Photodyn Ther [Internet]. 2015;12(3):401–7. Available from: <http://dx.doi.org/10.1016/j.pdpdt.2015.06.003>
36. Ornellas PO, Antunes LS, Motta PC, Mendonca C, Povoa H, Fontes K, et al. Antimicrobial Photodynamic Therapy as an Adjunct for Clinical Partial Removal of Deciduous Carious Tissue: A Minimally Invasive Approach. Photochem Photobiol [Internet]. 2018;94(6):1240–8. Available from: https://libaccess.mcmaster.ca/login?url=http://ovidsp.ovid.com/ovidweb.cgi?T=JS&CSC=Y&NEWS=N&PAGE=fulltext&D=emexa&AN=624882348 <http://sfx.scholarsportal.info/mcmaster?sid=OVID:embase&id=pmid:29923618&id=doi:10.1111%2Fphp.12966&issn=1751-1097&isbn=&volume>
37. 37. Friedrich JO, Adhikari NKJ, Beyene J. The ratio of means method as an alternative to mean differences for analyzing continuous outcome variables in meta-analysis: A simulation study. BMC Med Res Methodol. 2008;8(June).
38. Higgins JP, Li T, Deeks JJ. Choosing effect measures and computing estimates of effect. In: Cochrane Handbook for Systematic Reviews of Interventions. 2nd ed. 2019.
39. Wickham H. ggplot2: Elegant Graphics for Data Analysis. 1st ed. Springer; 2009. 212 p.
40. Lin L, Zhang J, Hodges JS, Chu H. Performing arm-based network meta-analysis in R with the pcnetmeta package. J Stat Softw. 2017;80(5).
41. Mavridis D, White IR, Higgins JPT, Cipriani A, Salanti G. Allowing for uncertainty due to missing continuous outcome data in pairwise and network meta-analysis. Stat Med. 2014;34(5):721–41.
42. Krunic J, Stojanovic N, Dukic L, Roganovic J, Popovic B, Simic I, et al. Clinical antibacterial effectiveness and biocompatibility of gaseous ozone after incomplete caries removal. Clin Oral Investig [Internet]. 2019;23(2):785–92. Available from: https://libaccess.mcmaster.ca/login?url=http://ovidsp.ovid.com/ovidweb.cgi?T=JS&CSC=Y&NEWS=N&PAGE=fulltext&D=emexa&AN=626110666 <http://sfx.scholarsportal.info/mcmaster?sid=OVID:embase&id=pmid:29858659&id=doi:10.1007%2Fs00784-018-2495-x&issn=1436-3771&isbn>
43. Goyal PA, Kumari R, Kannan VP, Madhu S. Efficacy and tolerance of papain gel with conventional drilling method: a clinico-microbiological study. J Clin Pediatr Dent [Internet]. 2015;39(2 CC-Oral Health):109‐112. Available from: <https://www.cochranelibrary.com/central/doi/10.1002/central/CN-01086804/full>
44. Uday Mohan PVM, Uloopi KS, Vinay C, Rao RC. In vivo comparison of cavity disinfection efficacy with APF gel, Propolis, Diode Laser, and 2% chlorhexidine in primary teeth. Contemp Clin Dent. 2016;7(1):45–50.
45. Melo MA, Rolim JP, Passos VF, Lima RA, Zanin IC, Codes BM, et al. Photodynamic antimicrobial chemotherapy and ultraconservative caries removal linked for management of deep caries lesions. Photodiagnosis Photodyn Ther [Internet]. 2015;12(4 CC-Oral Health):581‐586. Available from: <https://www.cochranelibrary.com/central/doi/10.1002/central/CN-01133866/full>
46. Steiner-Oliveira C, Aranha ACC, Ribeiro AC, Mayer MP, Eduardo CP. In vivo randomized antimicrobial effect of the photodynamic therapy on deciduous carious dentin. Med Oral Patol Oral Cir Bucal [Internet]. 2012;17:S220. Available from: <https://www.cochranelibrary.com/central/doi/10.1002/central/CN-01029650/full>
47. Ali AH, Koller G, Foschi F, Andiappan M, Bruce KD, Banerjee A, et al. Self-Limiting versus Conventional Caries Removal: A Randomized Clinical Trial. J Dent Res [Internet]. 2018;97(11):1207–13. Available from: https://libaccess.mcmaster.ca/login?url=http://ovidsp.ovid.com/ovidweb.cgi?T=JS&CSC=Y&NEWS=N&PAGE=fulltext&D=emexa&AN=629159710 <http://sfx.scholarsportal.info/mcmaster?sid=OVID:embase&id=pmid:29738286&id=doi:10.1177%2F0022034518769255&issn=1544-0591&isbn=>
48. Patri G, Sahu A. Role of herbal agents - Tea tree oil and aloe vera as cavity disinfectant adjuncts in minimally invasive dentistry-an in vivo comparative study. J Clin Diagnostic Res [Internet]. 2017;11(7):DC05–9. Available from: https://libaccess.mcmaster.ca/login?url=http://ovidsp.ovid.com/ovidweb.cgi?T=JS&CSC=Y&NEWS=N&PAGE=fulltext&D=emed18&AN=617118997 <http://sfx.scholarsportal.info/mcmaster?sid=OVID:embase&id=pmid:&id=doi:10.7860%2FJCDR%2F2017%2F27598.10147&issn=2249-782X&isb>
49. Hauser-gerspach I, Pfäffli-savtchenko V, Dähnhardt JE, Meyer J, Lussi A. Comparison of the immediate effects of gaseous ozone and chlorhexidine gel on bacteria in cavitated carious lesions in children in vivo. 2009;287–91.
50. Reddy MV, Sai Shankar A, Pentakota V, Kolli H, Ganta H, Katari P. Efficacy of antimicrobial property of two commercially available chemomechanical caries removal agents (Carisolv and Papacarie): An ex vivo study. J Int Soc Prev Community Dent. 2015;5(3):183.
51. Joshi JS, Roshan NM, Sakeenabi B, Poornima P, Nagaveni NB, Subbareddy V V. Inhibition of Residual Cariogenic Bacteria in Atraumatic Restorative Treatment by Chlorhexidine: Disinfection or Incorporation. Pediatr Dent [Internet]. 2017;39(4):308–12. Available from: https://libaccess.mcmaster.ca/login?url=http://ovidsp.ovid.com/ovidweb.cgi?T=JS&CSC=Y&NEWS=N&PAGE=fulltext&D=emed18&AN=623786994 <http://sfx.scholarsportal.info/mcmaster?sid=OVID:embase&id=pmid:29122072&id=doi:&issn=1942-5473&isbn=&volume=39&issue=4&spage=>
52. Aswathi K, Rani S, Athimuthu A, Prasanna P, Patil P, Deepali K. Comparison of efficacy of caries removal using polymer bur and chemomechanical caries removal agent: A clinical and microbiological assessment - An in vivo study. J Indian Soc Pedod Prev Dent. 2017;35(1):6–13.
53. Asal MA, Abdellatif AM, Hammouda HE. Clinical and microbiological assessment of carisolv and polymer bur for selective caries removal in primary molars. Int J Clin Pediatr Dent. 2021;14(3):357–63.
54. Modimi KV, Siddaiah SB, Chikkanarasaiah N, Rucha V, Abubakar SB, Dinraj K, et al. Microbiological Assessment of Carious Dentine using Chemomechanical Caries Removal and Conventional Hand Excavation in Primary and Permanent Teeth: A Clinical Study. J Int Oral Heal. 2016;8(7):760–6.
55. Moimaz SAS, Okamura AQC, Lima DC, Saliba TA, Saliba NA. Clinical and Microbiological Analysis of Mechanical and Chemomechanical Methods of Caries Removal in Deciduous Teeth. Oral Health Prev Dent [Internet]. 2019;17(3 CC-Oral Health):283‐288. Available from: <https://www.cochranelibrary.com/central/doi/10.1002/central/CN-01959797/full>
56. Düzyol E, Gürbüz T, Barış Ö. Antimicrobial Efficacy of Ozone Therapy on Cariogenic Bacteria. Meandros Med Dent J. 2021;22(1):1–7.
